# Altered states of consciousness caused by a mindfulness-based programme up to a year later: Results from a randomised controlled trial

**DOI:** 10.1371/journal.pone.0305928

**Published:** 2024-07-17

**Authors:** Julieta Galante, Jesús Montero-Marín, Maris Vainre, Géraldine Dufour, Javier García-Campayo, Peter B. Jones

**Affiliations:** 1 Department of Psychiatry, University of Cambridge, Cambridge, United Kingdom. (second affiliation: Contemplative Studies Centre, Melbourne School of Psychological Sciences, The University of Melbourne, Melbourne, Australia); 2 Department of Psychiatry, University of Oxford, Oxford, United Kingdom (second affiliation: Teaching, Research & Innovation Unit, Parc Sanitari Sant Joan de Déu, Sant Boi de Llobregat, Spain; third affiliation: Consortium for Biomedical Research in Epidemiology & Public Health (CIBER Epidemiology and Public Health - CIBERESP), Madrid, Spain); 3 National Institute for Health Research Collaboration for Leadership in Applied Health Research and Care East of England, Cambridge, United Kingdom (current affiliation: MRC Cognition and Brain Sciences Unit, University of Cambridge, Cambridge, United Kingdom and Institute of Psychology, University of Tartu, Tartu, Estonia); 4 University Counselling Service, University of Cambridge, Cambridge, United Kingdom (current affiliation: Psychotherapist, Therapeutic Consultations, Cambridge, United Kingdom; and School of Health and Social Care, University of Essex, Colchester, United Kingdom); 5 Department of Medicine, Psychiatry and Dermatology, University of Zaragoza, Zaragoza, Spain; 6 Department of Psychiatry, University of Cambridge, Cambridge, United Kingdom (second affiliation: National Institute for Health Research Applied Research Collaboration East of England, Cambridge, United Kingdom); Aarhus University: Aarhus Universitet, DENMARK

## Abstract

**Background:**

Mindfulness-based programmes (MBPs) have shown beneficial effects on mental health. There is emerging evidence that MBPs may also be associated with marked deviations in the subjective experience of waking consciousness. We aimed to explore whether MBPs can have a causal role in different types of such states.

**Methods:**

We conducted a pragmatic randomised controlled trial (ACTRN12615001160527). University of Cambridge students without severe mental illness were randomised to an 8-week MBP plus mental health support as usual (SAU), or to SAU alone. We adapted the Altered States of Consciousness Rating Scale (OAV, 0–100-point range) to assess spontaneous experiences in daily life, and included it as a post-hoc secondary outcome at the end of the one-year follow-up questionnaire. Two-part model analyses compared trial arms, and estimated dose-response effects of formal (meditation) and informal (daily activities) mindfulness practice during the year. Sensitivity analyses correcting for multiple comparisons were conducted.

**Results:**

We randomised 670 participants; 205 (33%) completed the OAV. In comparison with SAU, MBP participants experienced unity more frequently and intensively (two-part marginal effect (ME) = 6.26 OAV scale points, 95% confidence interval (CI) = 2.24, 10.27, p = 0.006, Cohen’s d = 0.33) and disembodiment more frequently (ME = 4.84, 95% CI = 0.86, 8.83, p = 0.019, Cohen’s d = 0.26). Formal practice predicted spiritual, blissful and unity experiences, insightfulness, disembodiment, and changed meanings. Informal practice predicted unity and blissful experiences. Trial arm comparisons and informal practice effects lost significance after corrections for multiple comparisons, but formal practice dose-response effects remained significant.

**Conclusions:**

Results provide a novel suggestion of causal links between mindfulness practice and specific altered states of consciousness. To optimise their impact, practitioners and teachers need to anticipate and handle them appropriately. Future studies need to confirm findings and assess mechanisms and clinical implications.

## Introduction

Mindfulness-based programmes (MBPs) are becoming very popular at a global scale. According to recent surveys, 15% of adults in the United Kingdom and 20% in Australia have learnt some form of mindfulness, while 5% of United States adults did it in 2017 alone [1, 2]. At least 79% of the medical schools in the United States offer some form of mindfulness training [3], as do over 600 companies globally (Jacobs, 2020). Accordingly, mindfulness research has grown rapidly since the beginning of the present century [4, 5].

MBPs often define mindfulness training, their key ingredient, as developing the skill of being attentive to the present moment (thoughts, physical sensations, and the surrounding environment), with an mindset of kindness and curiosity [6]. MBPs have shown beneficial mental and physical health effects in clinical and non-clinical contexts [7–11]. There are indications that MBPs may also elicit effects of diverse sensory nature and emotional tone. In a recent randomised controlled trial (RCT), it was demonstrated that individuals who underwent five sessions of mindfulness training reported more experiences of expanded self-boundaries and extending their spatial awareness beyond the physical body, compared to a control group engaged in active listening. [12].

Meditator surveys report a wide range of experiences in relation to meditative practices including mindfulness [13–15]. Experiences range from intense emotion, to altered perception and derealisation, which could be pleasant or unpleasant, and occasionally generate clinically relevant functional impairment. These empirical findings may not be surprising, since the meditation types practiced in MBPs involve practices that are said to deconstruct sensory aspects of the self [16]. Moreover, similar states are acknowledged by the traditions MBPs took their core practices from [15, 17–21]. However, these other effects of MBPs are much less researched than those directly related to health benefits [22]. It is important that we know more about the frequency, nature, implications and risk factors of the full range of MBP effects for facilitators and recipients to anticipate and handle them correctly [23].

Meditative experiences involving alterations of the senses, self, and body boundaries could be studied within the framework of altered states of consciousness (ASCs). An ASC represents a short-lasting “marked deviation in the subjective experience or psychological functioning of a normal individual from their usual waking consciousness” [24]. This deviation can encompass just one dimension of conscious experience, but it typically affects several aspects of it to varying degrees. ASCs can range from flow states to dissociative or psychedelic states, including different phenomena such as ego dissolution, disembodiment experiences, sensory alterations, impaired cognition, intense emotions, or changed meanings [25]. ASC can be induced by the application of pharmacological methods based on psychoactive substances, or by non-pharmacological methods, such as sensory deprivation, sensory overload, hypnosis, breathing techniques, or meditation practices [26–28]. Several psychometric scales have been developed to quantify the different aspects of ASCs [29–32]. There is a dearth of research investigating the effects of ASCs on physical and mental health, but existing data, mostly of a cross-sectional or qualitative nature, suggests that their effects on mental health and wellbeing depend heavily on the nature of the ASC, the context of the event, how much the ASC is expected, how it is dealt with, any external support available, and the meaning that the ASC has to the individual experiencing it [33–35]. Furthermore, not everyone ascribes the same emotional valence (pleasant or unpleasant) to the same ASCs [31, 32]. However, items commonly endorsed as unpleasant, such as experiencing disturbing images or extreme agitation, are associated with increased psychological distress [31], and may lead to functional impairment, which would constitute an adverse effect if caused by an intervention such as MBPs [36]. In any case, longitudinal research is needed to understand the possible causal pathways between meditation and ASC on the one hand, and ASC and health on the other hand.

In the context of the Mindful Student Study [37], a large RCT assessing the effectiveness of an MBP for promoting university students’ resilience to stress, as a secondary project we set out to explore the existence and nature of diverse sensory effects to generate evidence from longitudinal research in this emerging area. We operationalised these effects as ASC, and adapted an ASC scale to measure them. Specifically, we aimed to study: (a) whether having done the MBP would predict the presence and intensity of ASCs up to a year later, (b) associations between the amount of formal and informal mindfulness meditation practiced during the year and ASCs, (c) whether baseline sociodemographic factors would predict ASCs, and (d) whether ASCs would predict psychological distress measured at the end of the 1-year follow-up.

This was an exploratory sub-study because at the time there was no evidence to inform solid a-priori formal directional hypotheses. However, based on the roots and proposed mechanisms of MBPs, the scant research available, and anecdotal evidence, we anticipated that MBP participants might experience ASCs that alter one or more dimensions of the sense of self (e.g. self-other boundaries, embodiment, agency). Conversely, we did not expect that taking part in an MBP would predict, on average, the occurrence of highly unpleasant and impairing ASC such as terrors or paralysis. We anticipated that those who reported being more spiritual might experience more ASCs. Regarding the effects of ASCs on psychological distress, we thought that generally pleasant ASC might predict lower distress and vice-versa.

## Materials and methods

Below we will describe the main trial features and the measures and methods relevant to the work presented herein. For more details about procedures, measures and time points, please refer to the trial protocol [37], its main results [38], its one-year follow-up results [39], and other related publications [40, 41].

### Study design

The Mindful Student Study was an RCT confirming the effectiveness of a preventative group-based face-to-face MBP tailored to university students called Mindfulness Skills for Students (MSS). The main results showed that MSS in addition to mental health support as usual (SAU) reduced students’ psychological distress during the examination period (i.e., three to six months after randomisation) compared with access to mental health SAU alone. A reduction in distress while revising for exams (a few months after completing the MSS course) was considered an indicator of students’ resilience to stress. Participants were followed up for a year post randomization, revealing continued benefit from MSS in comparison with SAU alone. An extra set of questions was added to the one-year follow-up questionnaire for an exploratory post-hoc RCT analysis to compare ASCs between trial arms; the results of which are reported herein.

Regarding adverse events, as reported in the main trial publication, 20 MSS and 25 SAU participants triggered the adverse event protocol by exceeding pre-validated cut-off scores for psychological distress [38]. In addition, a participant withdrew from the MSS course, perceiving it as unhelpful and highlighting unwanted issues.

The trial received approval from Cambridge Psychology Research Ethics Committee on 25/08/2015 (PRE.2015.060), and the amendment to measure ASCs received approval on 09/09/2016. The trial protocol was submitted to the Australian New Zealand Clinical Trials Registry on 31/08/2015, before the study began (ACTRN12615001160527).

### Procedure

All the students at the University of Cambridge were invited to join this pragmatic RCT with two parallel arms and a one-to-one (1:1) allocation ratio. After being informed about the characteristics and study aims, volunteers provided written informed consent, completed baseline questionnaires, and were randomised via remote survey software (Qualtrics, concealed from researchers) using computer-generated random numbers (simple randomisation). The trial was co-produced with stakeholders and had an independent data monitoring and ethics committee.

Self-reported selection criteria were: (a) current undergraduate or postgraduate students at the University of Cambridge; (b) who believed they could attend at least seven sessions of the course. (c) not currently suffering from severe periods of anxiety or depression; (d) not experiencing severe mental illness such as hypomania or psychotic episodes; (e) no recent bereavement or major loss; (f) not experiencing any other serious mental or physical health problem that would affect their ability to engage with the course. Two cohorts of students were recruited and randomised in October 2015 (Cohort 1: N = 342), and January 2016 (Cohort 2: N = 274).

The MSS course was based on the manual described in the book ‘Mindfulness: A Practical Guide to Finding Peace in a Frantic World’ [42], and adapted for university students. An experienced and certified teacher taught the course in groups of up to 30 students (free at the point of delivery). The eight, weekly sessions lasted 75–90 minutes. Students were encouraged to also practice at home up to 25 minutes per day, during and after the course (follow-up period). Home practice included guided formal meditations (from here on: “formal practice’”) and other practices such as a mindful walking or mindful eating (from here on: “informal practice”).

### Data collection

Outcomes were collected using online questionnaires (web-based Qualtrics software) accessed by participants via a unique link. The ASC measure was a post-hoc measure (i.e. it was not in the initial trial protocol) included as an optional survey at the end of the one-year follow-up questionnaire. After collecting psychological distress, wellbeing and mindfulness practice data, participants were thanked with the offer of a gift card, and were asked “Do you have 5 more minutes to answer some questions about altered and different states of consciousness? We would really welcome your answers even if you haven’t experienced any altered states”, and if they said yes, they would be presented with the ASC measure. There was no extra payment for completing this measure.

#### ASC measure

To measure ASCs, we adapted the Studerus et al. version of the Altered States of Consciousness Rating Scale (known as OAV, an abbreviation that stands for the German names of the three original dimensions of the scale) [43, 44]. The OAV has been used for decades internationally in a broad range of research fields, mostly but not only in studies assessing the effects of psychoactive drugs. The scale underwent successive translations and modifications. In 2010, a thorough psychometric evaluation of the scale pooling data from 43 studies led to a revised multidimensional 42-item tool with 11 dimensions of ASC which have better psychometric properties than their predecessors [44]. The Studerus OAV dimensions are listed, along with their number of items and example items, as well as internal consistency values, in Table 1.

**Table 1:** Studerus OAV ASC dimensions, number of items, examples, and internal consistency values [44].

Dimension	Nº items	Examples	α
Experience of unity	*5*	*Everything seemed to come together as one*	0.88
Spiritual experience	*3*	*I had the feeling of being connected to a higher power*	0.77
Blissful state	*3*	*I experienced a profound peace in myself*	0.82
Insightfulness	*3*	*I gained clarity into connections that puzzled me before*	0.73
Disembodiment	*3*	*It seemed to me as though I did not have a body anymore*	0.82
Impaired control & cognition	*7*	*I had difficulty making even the smallest decision*	0.85
Anxiety	*6*	*I was afraid without being able to say exactly why*	0.89
Complex imagery	*3*	*My imagination was extremely vivid*	0.80
Elementary imagery	*3*	*I saw light or flashes of light with closed eyes*	0.84
Audiovisual synaesthesia	*3*	*The shapes of things seemed to change by sounds*	0.91
Changed meaning of percepts	*3*	*Things around me had a new strange meaning for me*	0.79

α: internal consistency values using Cronbach’s alpha

The Studerus OAV general instructions ask respondents to retrospectively rate their experience from the moment of psychoactive drug intake to the respective measuring time point. Each OAV item contains a statement that describes an ASC in the past tense (e.g., ‘‘It seemed to me that my environment and I were one”). Respondents are requested to describe the extent to which they experienced a similar state by placing marks on horizontal visual analogue scales anchored in the extremes (“No, not more than usual” on the left (rated as 0), and “Yes, very much more than usual” on the right (rated as 100)). We adapted the general instructions in the Studerus OAV so that respondents would include in their assessment any unusual experiences they may have had in the previous year. We also specifically requested them not to count any experiences happening under the influence of alcohol or drugs. The adapted OAV was piloted using a convenience sample of meditators and non-meditators (N = 54) to explore the clarity of the language and the sensitivity of the scale. Scoring high was more common among meditators, although several non-meditators, religious and non-religious, also reported ASCs. Both pleasant and unpleasant ASCs were endorsed by all groups and reporting several ASCs to varying degrees was common. Results suggested that the adapted ASC scale is sensitive to ASCs occurring in a range of contexts not related to drug or alcohol consumption. In fact, other studies have assessed non-pharmacologically induced ASC using the OAV [45–47]. The adapted OAV was also presented to our University of Cambridge student reference group for feedback on the suitability of the adapted scale for the student population. As a result, we replaced some words what sounded old-fashioned or confusing to UK English speakers (e.g. marionette with puppet and unusual with altered). This led to the final version of the adapted ASC scale for use in the trial (full scale can be found in the Appendix).

Some psychoactive drugs can induce hallucinogen-persisting perception disorders, popularly known as “flashbacks”, namely isolated ASCs days or weeks after the effects have ceased [48]. To discard the option that the ASCs reported were of this nature, after administering the adapted OAV, we asked participants whether any of the experiences reported had happened during meditation (they could select those that did), or due to “flashbacks” as a consequence of prior psychoactive drug use.

#### Other measures

Psychological distress was measured before randomization and after one year follow-up. We measured psychological distress using the Clinical Outcomes in Routine Evaluation Outcome Measure (CORE-OM). This 34-item scale has been widely used with UK university students [49]. The total mean score (range 0–4) is obtained by dividing the total score by the number of completed items; higher scores indicate more distress [50].

We monitored participants’ home-based mindfulness meditation practice throughout the follow-up period. Within the MSS arm, practice was self-reported via questions asked at post-intervention, exam term and one year follow-up time points (e.g. “Have you been practising mindfulness formally (meditation practice) since you finished your mindfulness course?”). They had to indicate how much they had practiced in an average week. We also collected meditation data from SAU participants. At each time point they were asked whether they had practised meditation elsewhere (e.g. “About how many hours have you spent meditating in total since March, when we last sent you a questionnaire?”) and the type of meditation they practiced. They were asked to indicate a range of hours. The mindfulness meditation data required pre-processing before analysis. In order to estimate a figure representing total hours of practice for each period of measurement we took the middle values of the ranges. For the MSS participants we multiplied the weekly value by the number of weeks in the period, and for the post-intervention time point, we also added half an hour of meditation for each course session attended. Finally, we added up the estimated hours of practice for each period, thus covering the full one-year follow-up.

Informal mindfulness practice was measured by asking to the intervention group participants for informal practice that they carried out throughout the follow-up period (e.g. “During the mindfulness course did you practice mindfulness informally at home (e.g. mindful living, mindful walks, mindful pauses, mindful attitudes)?”), including post-intervention, exam term, and one-year follow-up surveys. Response options ranged between 0 (“never”) and 4 (“very often”). A total score, calculated as the mean of the three questions, was used.

At baseline, we asked about the hours of meditation practice experience participants had previous to the trial. We also asked participants whether they considered themselves religious or spiritual (“Yes, very much”, “Yes, somewhat”, “I do not know”, and “No, I didn’t”). Finally, we included sociodemographic questions such as age, sex (“Woman”, “Man”), ethnicity (“White”, “Other”), disability (“Yes”, “No”), school (“Arts/Humanities”, “Biological Sciences”, “Clinical Medicine”, “Social Sciences”, “Physical Sciences”, “Technology”), degree level (“Undergraduate”, “Master”, “MPhil”, “PhD”), year (1^st^, 2^nd^, 3^rd^, 4^th^, 5^th^), nationality (“UK/EU”, “International”).

### Statistical analysis

Participant characteristics at pre-intervention were summarised using means and standard deviations (SDs) for continuous variables, and frequencies (percentages) for categorical variables. These characteristics were compared between the trial arms using the corresponding t-test or chi-squared test (or Fisher exact test), depending on their distribution.

Our outcome ASC variables were right-skewed and had many zero values (see S1-S11 Figs in S1 Appendix). Therefore, a two-part model analysis was conducted to explore the relationship between trial arm allocation and the presence and intensity of different dimensions of ASC. The two-part model, widely used for mixed discrete-continuous outcomes with a mass of zero values [51], comprised two steps. First, we employed a logit approach to calculate the probability of observing a zero vs. non-zero outcomes (absence vs. presence of ASCs). In the second part (values greater than zero), a Generalized Linear Model (GLM) with a log link and gamma distribution was utilised. This approach accommodates non-Gaussian distributions and provides an effective way to handle non-normal error distributions, as well as any heteroskedastic asymmetries and variances. To enhance interpretation, robust (sandwich) standard errors were used to calculate the corresponding marginal effect. A joint statistical test of significance for both parts of the two-part model employed a simultaneous covariance matrix of the sandwich estimator to correct the Wald test formula [52]. The following potential confounding variables were included: ASCs that happened as a result of the use of psychoactive drugs (i.e., flashbacks), as well as age, gender, and cohort (i.e., covariates that were pre-specified in the trial protocol [37]). All analyses followed the intention-to-treat (ITT) principle, whereby participants were analysed within the trial arm they had been randomised to, independently of whether they had adhered to the intervention. Additionally, we explored the potential association of formal and informal mindfulness meditation practice, separately, with the presence and intensity of ASCs, following the same analytical approach. For the formal practice analysis, total hours included three time-point measurements for both intervention and control group participants, while the informal practice analysis only involved the intervention group, since we only asked this group about their informal mindfulness practice.

We calculated the percentages of ASCs per OAV dimension reported to occur during meditation, as well as the percentages of ASCs that arose due to flashbacks, categorized by allocation group. Other explorations assessed the predictive value of baseline sociodemographic factors (including age, sex, previous meditation experience, disability, nationality, ethnicity, spirituality, and baseline psychological distress) on the presence and intensity of ASCs using the aforementioned two-part model, for the whole sample. Finally, we explored whether the presence of ASCs (zero vs. non-zero values) during the study period was a significant predictor of psychological distress at 1-year follow-up, for the whole sample, using a multivariable regression analysis.

Analyses were conducted using SPSS v25 and Stata v18 statistical software. We applied an overall (two-sided) alpha level of 0.05. Nevertheless, and despite the exploratory nature of the study, sensitivity analyses including corrections for multiple testing were performed for the probabilities in the joint models [53].

## Results

**Fig 1:**
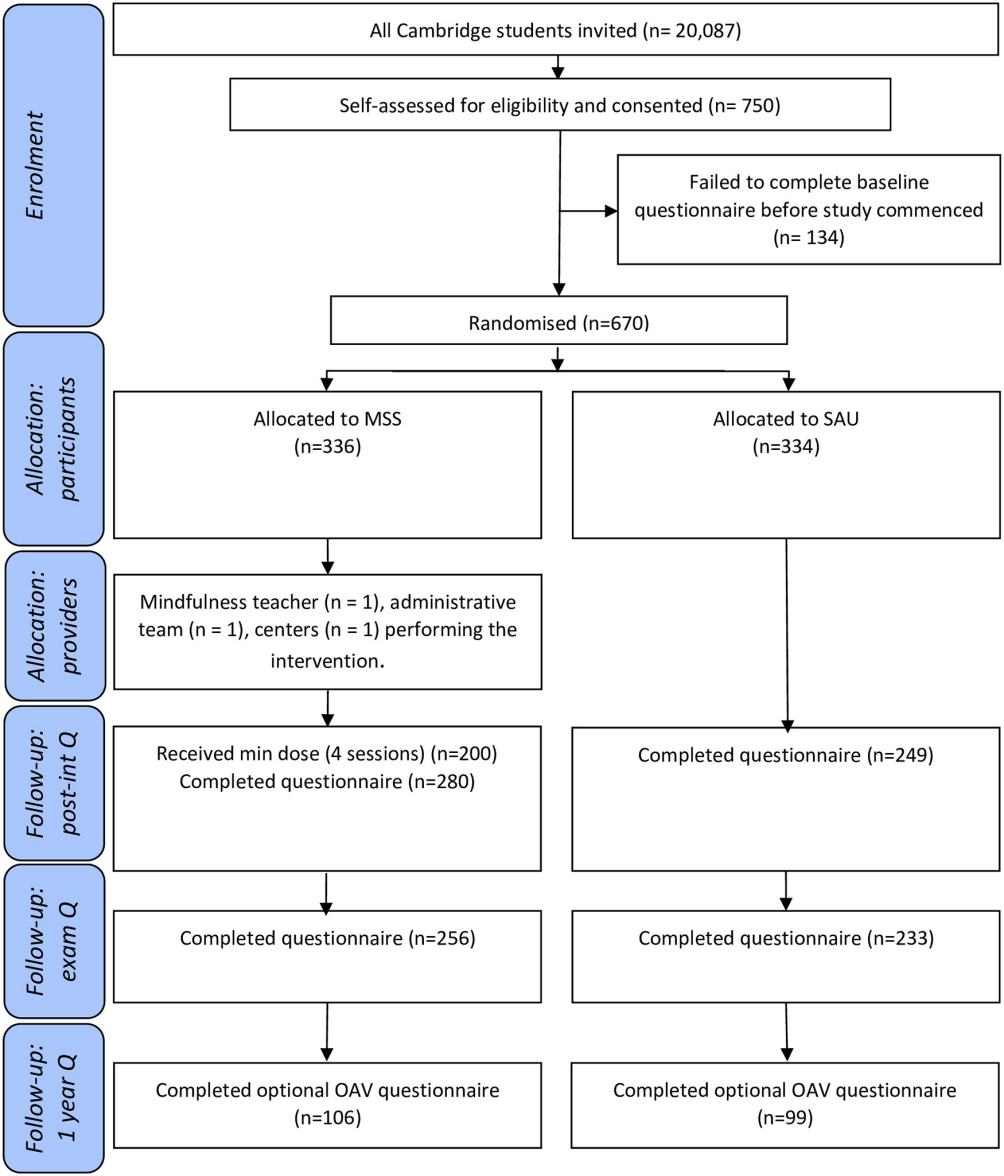
CONSORT 2010 flow diagram. No reasons were given for non-completion of the questionnaires.

In total, 670 participants were randomised between 28th September 2015 and 1st January 2016 to receive MSS (N = 336), or SAU (N = 334) [39, 40]. Of these, 106 MSS participants (32%) and 99 SAU participants (30%) completed the optional adapted OAV questionnaire, in addition to the sociodemographic, psychological distress, and practice questions, between 26 September 2016 and 11 October 2016 for Cohort 1, and between 10 January 2017 and 23 January 2017 for Cohort 2 (Fig 1). Participants’ characteristics of the study sample can be seen in Table 2. Being in the final year of studies and not having a white ethnicity reduced the likelihood of completing the OAV among all those randomised (S1 Table in S1 Appendix). Among those who completed the main part of the one-year follow-up questionnaire, ethnicity also predicted completion of the OAV (S2 Table in S1 Appendix).

**Table 2:** Baseline characteristics of the participants.

Variables/samples		Total sample (n = 205)		MSS (n = 106)		SAU (n = 99)		p
	*Range*	*M*	*SD*	*M*	*SD*	*M*	*SD*	
Age (years)	18‒53	23.80	5.81	24.51	6.51	23.04	4.89	0.073
Prior meditation experience (hours)	0‒100	5.91	17.35	6.95	18.07	4.79	16.55	0.375
Baseline distress	0.09‒2.85	0.95	0.51	0.99	0.49	0.92	0.53	0.343
	*Category*	*n*	*%*	*n*	*%*	*n*	*%*	
Gender	woman	139	67.8	68	64.2	71	71.7	0.247
	man	66	32.2	38	35.8	28	28.3	
Disability	yes	29	14.2	12	11.3	17	17.3	0.218
	no	175	85.8	94	88.7	81	82.7	
Spirituality	Do not know	18	9.2	10	10.1	8	8.2	0.764
	No	116	59.2	55	55.6	61	62.9	
	Yes, somewhat	48	24.5	28	28.3	20	20.6	
	Yes, very much	12	6.1	5	5.1	7	7.2	
Ethnicity	White	154	75.1	79	74.5	75	75.8	0.694
	Other	42	20.4	23	21.7	19	19.2	
School	Arts/Humanities	35	17.1	16	15.1	19	19.2	0.588
	Biological Sciences	54	26.3	25	23.6	29	29.3	
	Clinical Medicine	17	8.3	8	7.5	9	9.1	
	Social Sciences	53	25.9	32	30.2	21	21.2	
	Physical Sciences	23	11.2	14	13.2	9	9.1	
	Technology	23	11.2	11	10.4	12	12.1	
Degree level	Undergraduate	108	52.7	48	45.3	60	60.6	0.159
	Master	11	5.4	7	6.6	4	4.0	
	MPhil	20	9.8	13	12.3	7	7.1	
	PhD	66	32.2	38	35.8	28	28.3	
Year	1^st^	103	50.2	58	54.7	45	45.5	0.314
	2^nd^	41	20.0	16	15.1	25	25.3	
	3^rd^	41	20.0	23	21.7	18	18.2	
	4^th^	17	8.3	7	6.6	10	10.1	
	5^th^	3	1.5	2	1.9	1	1.0	
Nationality	UK/EU	166	81.0	87	82.1	79	79.8	0.786
	International	38	18.5	19	17.9	19	19.2	
Cohort	1^st^	111	54.1	58	54.7	53	53.5	0.889
	2^nd^	94	45.9	48	45.3	46	46.5	

MSS: mindfulness skills for students; SAU: support as usual; M: mean; SD: standard deviation; n: frequencies; %: percentages; p: p-value associated with the corresponding contrast test.

S1-S11 Figs in S1 Appendix present a graphical representation of the distribution of the OAV variables for the total sample and by trial arm allocation. Table 3 shows descriptive data for the OAV dimensions in each part of the two-part model, categorised by trial arm. As depicted in Table 4, the marginal (i.e., incremental) effect on the experience of unity for MSS compared with SAU was 6.26 OAV scale points (95% CI = 2.24, 10.27), and the joint test of significance was statistically significant (χ2 (df) = 10.14 (2); p = .006). This corresponds to a Cohen’s d effect size of 0.33, typically interpreted as small-to-medium [54]. Similarly, the marginal effect on disembodiment for MSS compared with SAU was 4.84 OAV scale points (95% CI = 0.86, 8.83), and the joint test of significance was statistically significant (χ2 (df) = 7.92 (2); p = .019). This corresponds to a Cohen’s d effect size of 0.26 (small). However, these relationships were no longer significant after correcting for multiple comparisons.

**Table 3:** Descriptive data for experiencing Altered States of Consciousness.

ASC dimensions	value	MSS			SAU		
		n	%	M (SD)	n	%	
Experience of Unity	zero	39	42.4		53	57.6	
	>zero	61	58.1	18.03 (19.28)	44	41.9	14.04 (17.45)
Spiritual Experience	zero	49	49.0		54	55.7	
	>zero	51	51.0	22.82 (20.16)	43	44.3	19.44 (21.30)
Blissful State	zero	25	25.0		32	33.0	
	>zero	75	75.0	27.17 (26.78)	65	67.0	21.77 (20.74)
Insightfulness	zero	43	43.0		47	48.5	
	>zero	57	57.0	19.42 (20.67)	50	51.5	19.35 (18.90)
Disembodiment	zero	58	58.0		72	74.2	
	>zero	42	42.0	20.11 (22.83)	25	25.8	14.62 (14.52)
Impaired Control and Cognition	zero	39	39.0		33	34.0	
	>zero	61	61.0	14.73 (15.88)	64	66.0	15.53 (17.01)
Anxiety	zero	46	46.0		35	36.1	
	>zero	54	54.0	15.54 (19.30)	62	63.9	15.72 (18.22)
Complex Imagery	zero	59	59.0		59	60.8	
	>zero	41	41.0	18.71 (19.86)	38	39.2	21.13 (19.92)
Elementary Imagery	zero	56	56.0		61	62.9	
	>zero	44	44.0	21.93 (21.96)	36	37.1	15.86 (20.55)
Audio-Visual Synaesthesia	zero	76	76.0		86	88.7	
	>zero	24	24.0	11.08 (12.83)	11	11.3	12.28 (19.50)
Changed Meaning of Percepts	zero	48	48.0		60	61.9	
	>zero	52	52.0	18.44 (20.80)	37	38.1	16.81 (16.74)

ASC: altered states of consciousness. MSS: mindfulness skills for students. SAU: support as usual. M: mean; SD: standard deviation; n: frequencies; %: percentages

**Table 4:** Joint model (Zero and Non-Zero Parts) for experiencing Altered States of Consciousness (ASC) by trial arm.

ASC dimension	B	SE	95% CI	χ^2^ (df)	p
Experience of Unity	6.26	2.05	2.24, 10.27	10.14 (2)	.006^†^
Spiritual Experience	3.23	2.41	-1.50, 7.96	1.77 (2)	.412
Blissful State	6.09	3.41	-0.59, 12.77	3.28 (2)	.194
Insightfulness	1.93	2.34	-2.65, 6.51	0.88 (2)	.645
Disembodiment	4.84	2.03	0.86, 8.83	7.92 (2)	.019^†^
Impaired Control and Cognition	-1.60	2.08	-5.67, 2.48	0.78 (2)	.676
Anxiety	-2.08	1.98	-5.96, 1.81	1.87 (2)	.393
Complex Imagery	-0.41	2.34	-4.99, 4.17	0.31 (2)	.857
Elementary Imagery	5.15	2.37	0.50, 9.80	5.20 (2)	.074
Audio-Visual Synaesthesia	0.93	1.18	-1.39, 3.25	5.07 (2)	.079
Changed Meaning of Percepts	3.05	2.26	-1.39, 7.48	3.20 (2)	.202

B = marginal effects for the Allocation Group (MSS vs. SAU) predictor. SE: (Robust) Standard Error. 95% CI: 95% Confidence Interval for the marginal effects. p: p-value associated with the joint statistical test of significance including the parameters of the two-part model (logit in the first part (zero vs. >zero), and GLM with a log link and gamma distribution in the second part (>zero)). Models included the following predictors: allocation group (MSS vs. SAU), ASC as a consequence of flashbacks, as well as age, sex, and cohort (design variables).

^†^This relationship was no longer significant when the Benjamini-Hochberg correction was applied to correct for multiple comparisons.

Results comparing trial arms for each part of the two-part model separately can be seen in S3 and S4 Tables in S1 Appendix. The odds ratios (ORs) of frequencies between arms suggest that being offered the MSS course doubled the odds of having ASCs of unity (OR = 2.01; 95% CI = 1.09, 3.67; p = .024), disembodiment (OR = 2.29; 95% CI = 1.23, 4.26; p = .009), and audio-visual synaesthesia (OR = 2.41; 95% CI 1.10, 5.27; p = .028). ASC experiences of unity were significantly more intense in the MSS group than in the SAU group (MSS: M (SD) = 18.03 (19.28); SAU: M (SD) = 14.04 (17.45); B = 0.51 (95% CI = 0.04, 0.97); p = .032). However, these differences were no longer significant when the correction for multiple comparisons was applied. S12-S22 Figs in S1 Appendix show a graphical representation of the relationships between the observed and predicted values for each part of the two-part models.

The average total amount of formal mindfulness meditation practice during the entire study period in the total sample was M (SD) = 17.7 (25.3) hours, with M (SD) = 30.3 (26.9) hours in the MSS group and M (SD) = 4.6 (17.1) hours in the SAU group (refer to S23 Fig in S1 Appendix for a graphical representation). Most participants reported practicing mindfulness informally ’sometimes’ or ’often’ (see S24 Fig in S1 Appendix).

Next, we explored the relationships between the total hours of formal mindfulness practice and the presence and intensity of ASC experiences, irrespective of trial arm. As indicated in Table 5, the marginal (i.e., incremental) effect on the experience of unity for formal mindfulness practice was 0.17 OAV scale points for each extra hour of meditation (95% CI = 0.08, 0.26), and the joint test of significance was statistically significant (χ2 (df) = 20.94 (2); p < .001). Similarly, the marginal effect on spiritual experience for formal mindfulness practice was 0.15 (95% CI = 0.05, 0.25), and the joint test of significance was statistically significant (χ2 (df) = 15.12 (2); p < .001). The marginal effect on the blissful state for formal mindfulness practice was 0.30 (95% CI = 0.16, 0.45), and the joint test of significance was statistically significant (χ2 (df) = 23.03 (2); p < .001). Additionally, the marginal effect on insightfulness for formal mindfulness practice was 0.12 (95% CI = 0.03, 0.21), with a statistically significant joint test (χ2 (df) = 8.14 (2); p = .017). The marginal effect on disembodiment for formal mindfulness practice was 0.11 (95% CI = 0.05, 0.17), and the joint test of significance was statistically significant (χ2 (df) = 16.19 (2); p < .001). Finally, the marginal effect on the changed meaning of percepts for formal mindfulness practice was 0.11 (95% CI = 0.02, 0.19), with a statistically significant joint test (χ2 (df) = 7.62 (2); p = .022). All these relationships remained significant after correcting for multiple comparisons. Detailed results for each part of the two-part model separately are provided in S5 and S6 Tables in S1 Appendix. S25-S35 Figs in S1 Appendix offer a graphical representation of the relationships between the observed and predicted values for each part of the two-part model.

**Table 5:** Joint Model (Zero and Non-Zero Parts) for experiencing Altered States of Consciousness (ASC) with formal mindfulness practice as a predictor.

ASC dimension	B	SE	95% CI	χ^2^ (df)	p
Experience of Unity	0.17	0.05	0.08, 0.26	20.94 (2)	<.001
Spiritual Experience	0.15	0.05	0.05, 0.25	15.12 (2)	<.001
Blissful State	0.30	0.07	0.16, 0.45	23.03 (2)	<.001
Insightfulness	0.12	0.05	0.03, 0.21	8.14 (2)	.017
Disembodiment	0.11	0.03	0.05, 0.17	16.19 (2)	<.001
Impaired Control and Cognition	-0.07	0.04	-0.14, -0.01	5.51 (2)	.064
Anxiety	-0.06	0.05	-0.15, 0.03	2.11 (2)	.347
Complex Imagery	0.04	0.05	-0.06, 0.13	1.33 (2)	.514
Elementary Imagery	0.05	0.04	-0.03, 0.14	1.73 (2)	.422
Audio-Visual Synaesthesia	0.06	0.05	-0.04, 0.16	4.10 (2)	.129
Changed Meaning of Percepts	0.11	0.04	0.02, 0.19	7.62 (2)	.022

B = marginal effects for the formal mindfulness practice predictor. SE: (Robust) Standard Error. 95% CI: 95% Confidence Interval for the marginal effects. p: p-value associated with the joint statistical test of significance including the parameters of the two-part model (logit in the first part (zero vs. >zero), and GLM with a log link and gamma distribution in the second part (>zero)). Models included the following predictors: formal mindfulness practice, ASC as a consequence of flashbacks, as well as age, sex, and cohort (design variables). All the significant relationships were significant when the Benjamini-Hochberg correction was applied to correct for multiple comparisons.

As demonstrated in S7 Table in S1 Appendix, in the MSS trial arm, the marginal (incremental) effect on the experience of unity for informal mindfulness practice was 5.45 (95% CI = 0.56, 10.35), and the joint test of significance was statistically significant (χ2 (df) = 6.17 (2); p = .045). Additionally, the marginal effect on the blissful state for informal mindfulness practice was 8.87 (95% CI = 0.56, 10.35), and the joint test of significance was statistically significant (χ2 (df) = 6.90 (2); p = .032). However, these relationships were no longer significant after correcting for multiple comparisons. Detailed results for each part of the two-part model separately are presented in S8 and 9 Tables in S1 Appendix.

Due to an error during data collection, only participants who reported having meditated in the prior six months saw the question about whether ASCs happened during meditation. Therefore, we based the description of the proportion of ASCs that occurred during meditation on this sub-sample (N = 73). As indicated in S10 Table in S1 Appendix, 42.5% reported unity experiences during meditation, 46.6% blissful states, 24.7% insightfulness experiences, 28.8% disembodiment experiences, and 20.6% changed meaning of percepts. Flashback-related ASCs were rare (ranging from 0.5% of the total sample for complex imagery to 4.1% of the total sample for the experience of unity). There was no evidence that mindfulness training modifies the risk of experiencing flashback-related ASCs among drug users (S11 Table in S1 Appendix).

S12 Table in S1 Appendix presents an exploration of the potential predictive value of baseline sociodemographic factors, including baseline psychological distress, on the presence and intensity of ASCs. Greater psychological distress prior to the trial was significantly associated with higher experiences of disembodiment (marginal effect: 6.58; 95% CI = 1.15, 12.00; joint test of significance: χ2 (df) = 12.39 (2); p = .002), impaired control and cognition (marginal effect: 6.91; 95% CI = 1.84, 11.99; joint test of significance: χ2 (df) = 8.57 (2); p = .014), anxiety (marginal effect: 9.71; 95% CI = 4.40, 15.02; joint test of significance: χ2 (df) = 17.07 (2); p < .001), complex imagery (marginal effect: 5.54; 95% CI = 0.56, 10.53; joint test of significance: χ2 (df) = 6.15 (2); p = .046) and elementary imagery (marginal effect: 5.66; 95% CI = 0.24, 11.55; joint test of significance: χ2 (df) = 6.69 (2); p = .035) during the study period. Furthermore, the more spiritual participants considered themselves at the time they signed up for the study, the higher the spiritual ASC experience during the study (marginal effect: 4.75; 95% CI = 1.25, 8.24; joint test of significance: χ2 (df) = 7.85 (2); p = .020). Interestingly, previous meditation experience was associated with higher experiences of unity (marginal effect: 0.16; 95% CI = 0.02, 0.30; joint test of significance: χ2 (df) = 6.68 (2); p = .035) and blissful state (marginal effect: 0.30; 95% CI = 0.08, 0.52; joint test of significance: χ2 (df) = 7.27 (2); p = .026), as well as lower experiences of complex imagery (marginal effect: -0.16; 95% CI = -0.30, -0.02; joint test of significance: χ2 (df) = 7.32 (2); p = .025). Nevertheless, only psychological distress prior to the trial remained as a significant predictor of disembodiment and anxiety experiences after correcting for multiple comparisons.

S13 Table in S1 Appendix shows the potential predictive value of the presence of ASCs during the study period on the psychological distress outcome at 1-year follow-up. Only the ASC of anxiety was a significant predictor of psychological distress.

## Discussion

The large RCT within which the present exploratory sub-study was conducted found that MBPs can promote, on average, university students’ resilience to stress. In this context, our sub-study results suggest that low levels of mindfulness practice typical of MBPs may cause some ASCs, with our most robust finding being that of a dose-response relationship such that more mindfulness meditation predicts more ASCs. Although differences between trial arms were sensitive to multiple testing correction, we have detected these effects despite using ITT analyses, which include everyone randomised regardless of how many mindfulness course sessions they attended or how much they have meditated at home (many participants in the intervention group were not meditating at all during the follow-up). Moreover, the conservative use of two-part models and robust standard errors to correctly represent our highly asymmetrical distributions may have resulted in suboptimal statistical power [55]. While subject to residual confounding, meaning that causality claims are harder to make, the dose-response relationships that we found were robust to multiple testing correction.

Taken together, our results indicate the possibility of a causal role of mindfulness training in unity and disembodiment experiences. For unity in particular, the average effect size was small to medium, consisting of both higher frequency (double) and intensity among those in the MSS group, and a dose-response pattern with formal and informal practice. For disembodiment, there was a small average effect size, consisting of a more-than-doubled frequency in the MSS group and a dose-response pattern with formal practice. Moreover, a substantial proportion of these two types of experiences (43% and 29% respectively) were reported to happen during meditation. In unity experiences there is a sense that borders dissolve and everything, sometimes including the sense of time, is perceived in an integrated way. Disembodiment experiences may imply an element of separation from the body, but they often consist of a floating sensation or a dissolution of body boundaries, which may facilitate strong unity experiences. Our results align with a recent study where participants, following five sessions of mindfulness training, more commonly described experiencing a sense of relaxed self-boundaries and broadening their spatial awareness beyond the physical body, in contrast to a control group engaged in active listening [12]. Other studies also support mindfulness practice effects on self and time perception [56, 57]. If meditation types practiced in MBPs involve self-deconstructive practices, as in the case of mindfulness-based cognitive therapy (MBCT) [58], from which the MBCT-FP programme used in the present study has been developed [42], this makes sense [16]. There may be differences between MBPs, for example less emphasis on self-deconstruction and more on skills-training, that could change their impact on ASCs.

We found dose-response evidence for a link between mindfulness practice and bliss. Formal practice may also lead to spiritual experiences, insightfulness, disembodiment, and changed meaning of percepts. Correlations between meditation and spiritual experiences have been found using the Mysticism Scale, which includes bliss, insightfulness and new meaning experiences [57]. However, these correlations may be explained by increased practice among those with pre-existing tendencies to experience ASCs. We found weak evidence for synaesthesia; a previous investigation of MBP adverse effects found no evidence of increased synaesthesia [36].

There is scant research on the possible mechanisms that could explain the increased incidence of ASCs with mindfulness practice. For example, although mindfulness meditation has been frequently theorised to impact self-related processes, there is a dearth of prospective longitudinal research in the more embodied dimensions of selfhood [22]. Disembodiment effects may be mediated by decentering processes [59]. A small RCT found increases in absorption after five weeks of mindfulness meditation [60]. Absorption, a disposition to become absorbed in mental imagery, is typically measured using the Tellegen Absorption Scale, which include subscales that measure ASCs, such as synaesthesia, and “expanded awareness” [61]. Our results could also be explained by mindfulness practitioners perceiving experiences that happen anyway in the general population under the light of meditation practice. Mindfulness meditation may make these experiences more salient, and perhaps even meaningful, such that memories of those experiences were more readily available when asked about them. Also, some of the OAV scale items could be interpreted metaphorically, and mindfulness course participants may interpret these less metaphorically than control group participants. For example, ‘feeling like a puppet’ may be literally feeling you are not in control of your limbs, or just feeling someone is verbally manipulating you so that you make the decisions they want you to make.

Although our trial suggests a causal role of mindfulness practice in the incidence of ASCs up to a year later, it has several limitations that reduce the reliability of our results. Given that this was an optional questionnaire, there was a high proportion of missing data, although it is a positive aspect that completion rates were similar across trial arms. The primary outcome of this trial was analysed using multiple imputation assuming missing at random data, and it was robust to a complete case sensitivity analysis [38]. We note, however, that there is much more missing data in the ASC outcomes, and as a result of this, utilising imputation techniques for ASC data would render the modelling very complex, unstable, and thus unsuitable for an exploratory study. We nevertheless recognize the possibility that data are missing not at random and therefore affecting the generalisability our results. Furthermore, we only collected OAV data once, at the end of the follow up, so recall bias could be at play, with older and less intense experiences consequently under-reported. This bias may affect trial arms equally, therefore not impacting between-arm comparisons, but it may have affected our estimates of associations between ASCs and psychological distress. Other limitations are that the adaptation of the OAV scale for use in this trial was not psychometrically validated, and that the ASCs outcome and analyses were not pre-specified in the trial protocol. We have performed multiple comparisons assessing the different dimensions of ASCs, which increases the chance of spurious statistical significance. Nevertheless, we carried out sensitivity analyses adjusting for multiple comparisons. Since this is an exploratory analysis in an area that has not been researched before, we present the original results together with the adjustments, and note that additional dedicated studies are needed to confirm findings [62].

Although the OAV scale has been helpful in this trial and could be used in future replication studies, it could be improved for its use in non-psychedelic studies in order to capture the range and intensity of emergent ASC more accurately. There could also be ASC scale developments specific to mindfulness meditators. However, one of the convenient features of the OAV as used in this study is that the scale does not mention meditation as a cause of ASCs, so it can be used among non-meditators too for comparisons. Another advantage of the OAV is that it enquires about a wide range of experiences, rather than limiting them to those explored by problematic “mystical experience” scales [32]. Notwithstanding, we are undertaking further psychometric work to focus on the range, intensity and valence of experiences most commonly found among meditators and devise their factorial dimensions [31]. Further psychometric work is also needed to model the item response of the OAV scale, instead of treating all items equally.

If findings are confirmed, there will be a need to understand ASCs’ impact, patterns and implications [17]. The ASCs that seemed to happen more and more intensely with mindfulness meditation do not have intrinsically unpleasant characteristics [63]. On the contrary, some such as bliss are to be highly pleasant. However, some ASCs such as disembodiment or altered sense of self can be pleasant [64], or startling, even alarming [15, 65]. They are also involved in unpleasant experiences that university students reported about their mindfulness course participation in a recent qualitative study: *“as though I need to find a way to maintain my sense of self; as if I am not actually present; as if I am watching myself; hyper aware; as though I am checking out; like zoning out; as if I am losing spontaneity and second guessing myself; increased self-talk; numbness; as if I am on mute; as if I have left my body; that my body is freaking out*” [66]. The fact that greater baseline distress in our sample predicted more disembodiment and anxiety supports the suggestion that for people who are not well, disembodiment experiences may be problematic, although there is a possibility that the intervention may have influenced this relationship. Disturbances in the sense of self such as disembodiment can be associated with psychopathology, for example dissociation and depersonalisation [67].

It is known that experiences can be interpreted as pleasant or unpleasant depending on the context, so it is key that they are anticipated and managed well [33, 68, 69]. There may well be cultural differences that influence ASC intensity and how they impact individuals’ wellbeing, for example, different relationships with mystical experiences. This needs to be investigated working in collaboration with disciplines such as anthropology, sociology, history and philosophy. Furthermore, the training in acceptance of experience that mindfulness courses include may also change they ways in which ASCs are appraised. A trial has found non-statistically significant increases in hallucination-like experiences, but reductions in anxiety caused by them, among university students randomised to mindfulness compared with a video-forum intervention [70]. Future studies could assess whether MBPs moderate associations between ASC and mental health within a single statistical model that takes temporally ordered data into account. There may be multiple complexities around the impact of mindfulness-induced ASCs on mental health and wellbeing. It may be that some people stop meditating regularly due to encountering experiences which they do not know how to manage. Should the increased occurrence of ASCs among those who do an eight week mindfulness course be confirmed, prospective practitioners will need to be adequately informed about this possibility, and further research is warranted to guide mindfulness teachers and practitioners on how to anticipate and manage ASCs.

## Supporting information

S1 Appendix Supplemental materials. (PDF)

S1 Checklist Reporting checklist for randomised trial. (DOCX)

S1 Protocol (PDF)

## References

[1] Simonsson O, Fisher S, Martin M. Awareness and Experience of Mindfulness in Britain. Sociological Research Online. 2020;26(4):833–52. doi: 10.1177/1360780420980761

[2] Dib J, Comer, J., Wootten, A., Buhagiar, K. State of Mind 2021 Report. Melbourne: Smiling Mind; 2021.

[3] Barnes N, Hattan P, Black DS, Schuman-Olivier Z. An Examination of Mindfulness-Based Programs in US Medical Schools. Mindfulness. 2017;8(2): 489–94. doi: 10.1007/s12671-016-0623-8

[4] Van Dam NT, van Vugt MK, Vago DR, Schmalzl L, Saron CD, Olendzki A, et al. Mind the Hype: A Critical Evaluation and Prescriptive Agenda for Research on Mindfulness and Meditation. Perspectives on psychological science: a journal of the Association for Psychological Science. 2018;13(1):36–61. Epub 2017/10/11. doi: 10.1177/1745691617709589 . 29016274 PMC5758421

[5] Pagnini F, Philips D. Being mindful about mindfulness. The Lancet Psychiatry. 2015;2(4):288–9. doi: 10.1016/S2215-0366(15)00041-3 26360065

[6] Kabat-Zinn J. Full Catastrophe Living, Revised Edition: How to cope with stress, pain and illness using mindfulness meditation. 2 ed. London: Piatkus; 2013.

[7] Goyal M, Singh S, Sibinga EMS, Gould NF, Rowland-Seymour A, Sharma R, et al. Meditation programs for psychological stress and well-being: a systematic review and meta-analysis. JAMA internal medicine. 2014;174(3):357–68. doi: 10.1001/jamainternmed.2013.13018 24395196 PMC4142584

[8] Dawson AF, Brown WW, Anderson J, Datta B, Donald JN, Hong K, et al. Mindfulness-based Interventions for University Students: A Systematic Review and Meta-analysis of Randomized Controlled Trials. Applied Psychology: Health and Well-Being. 2020;12(2):384–410. 31743957 10.1111/aphw.12188

[9] Michael de Vibe AB, Sabina Fattah, Dyrdal Gunvor M, Halland Even, Tanner-Smith Emily E. Mindfulness-based stress reduction (MBSR) for improving health, quality of life and social functioning in adults: a systematic review and meta-analysis. Campbell Systematic Reviews. 2017;13:1–264. doi: 10.4073/csr.2017.11

[10] Goldberg SB, Tucker RP, Greene PA, Davidson RJ, Wampold BE, Kearney DJ, et al. Mindfulness-based interventions for psychiatric disorders: A systematic review and meta-analysis. Clinical psychology review. 2018;59:52–60. Epub 2017/11/12. doi: 10.1016/j.cpr.2017.10.011 29126747 PMC5741505

[11] Galante J, Friedrich C, Dawson AF, Modrego-Alarcón M, Gebbing P, Delgado-Suárez I, et al. Mindfulness-based programmes for mental health promotion in adults in non-clinical settings: A systematic review and meta-analysis of randomised controlled trials. PLOS Medicine. 2021;18(1):e1003481. Epub 2021/01/12. doi: 10.1371/journal.pmed.1003481 33428616 PMC7799763

[12] Hanley AW, Dambrun M, Garland EL. Effects of Mindfulness Meditation on Self-Transcendent States: Perceived Body Boundaries and Spatial Frames of Reference. Mindfulness. 2020;11(5):1194–203. doi: 10.1007/s12671-020-01330-9 33747250 PMC7968136

[13] Cebolla A, Demarzo M, Martins P, Soler J, Garcia-Campayo J. Unwanted effects: Is there a negative side of meditation? A multicentre survey. PloS one. 2017;12(9):e0183137. Epub 2017/09/06. doi: 10.1371/journal.pone.0183137 28873417 PMC5584749

[14] Schlosser M, Sparby T, Voros S, Jones R, Marchant NL. Unpleasant meditation-related experiences in regular meditators: Prevalence, predictors, and conceptual considerations. PloS one. 2019;14(5):e0216643. Epub 2019/05/10. doi: 10.1371/journal.pone.0216643 . 31071152 PMC6508707

[15] Lindahl JR, Britton WB. ‘I Have This Feeling of Not Really Being Here’ Buddhist Meditation and Changes in Sense of Self. 2019.

[16] Dahl CJ, Lutz A, Davidson RJ. Reconstructing and deconstructing the self: cognitive mechanisms in meditation practice. Trends in cognitive sciences. 2015;19(9):515–23. Epub 2015/08/02. doi: 10.1016/j.tics.2015.07.001 26231761 PMC4595910

[17] Galante J, Grabovac A, Wright M, Ingram DM, Van Dam NT, Sanguinetti JL, et al. A framework for the empirical investigation of mindfulness meditative development. Mindfulness. 2023;14:1054–67.

[18] Upatissa. The path of freedom (Vimuttimagga). Kandy; Sri Lanka: Buddhist Publication Society; 1995.

[19] Bodhi B. Comprehensive Manual of Abhidhamma: Pariyatti Publishing; 2012.

[20] Buddhaghosa A. The Path of Purification: Visuddhimagga New edition. ed: Buddhist Pubns Society; 1990.

[21] Sedlmeier P. Meditation and altered states of consciousness. Journal of Consciousness Studies. 2018;25(11–12):73–101.

[22] Britton WB, Desbordes G, Acabchuk R, Peters S, Lindahl JR, Canby NK, et al. From Self-Esteem to Selflessness: An Evidence (Gap) Map of Self-Related Processes as Mechanisms of Mindfulness-Based Interventions. Frontiers in psychology. 2021;12:730972. Epub 2021/12/10. doi: 10.3389/fpsyg.2021.730972 34880805 PMC8645694

[23] Lindahl JR, Britton WB, Cooper DJ, Kirmayer LJ. Challenging and Adverse Meditation Experiences: Toward a Person-Centered Approach. In: Farias M, Brazier D, Lalljee M, editors. The Oxford Handbook of Meditation: Oxford University Press; 2020.

[24] Dittrich A. The standardized psychometric assessment of altered states of consciousness (ASCs) in humans. Pharmacopsychiatry. 1998;31(80–84). doi: 10.1055/s-2007-979351 9754838

[25] Schmidt TT, Majić T. Empirische Untersuchung veränderter Bewusstseinszuständ. Handbuch Psychoaktive Substanzen: Springer; 2016.

[26] Hirschfeld T, Schmidt TT. Dose-response relationships of psilocybin-induced subjective experiences in humans. J Psychopharmacol. 2021;35(4):384–97. doi: 10.1177/0269881121992676 . 33663259 PMC8058832

[27] Bartossek MT, Kemmerer J, Schmidt TT. Altered states phenomena induced by visual flicker light stimulation. PloS one. 2021;16(7):e0253779. Epub 2021/03/06. doi: 10.1371/journal.pone.0253779 34197510 PMC8248711

[28] Corlett PR, Frith CD, Fletcher PC. From drugs to deprivation: a Bayesian framework for understanding models of psychosis. Psychopharmacology (Berl). 2009;206(4):515–30. Epub 2021/07/02. doi: 10.1007/s00213-009-1561-0 19475401 PMC2755113

[29] Dworatzyk K, Jansen T, Schmidt TT. Phenomenological assessment of psychedelics induced experiences: Translation and validation of the German Challenging Experience Questionnaire (CEQ) and Ego-Dissolution Inventory (EDI). PloS one. 2022;17(3):e0264927. Epub 2009/05/29. doi: 10.1371/journal.pone.0264927 35294453 PMC8926265

[30] Schmidt TT, Berkemeyer H. The Altered States Database: Psychometric Data of Altered States of Consciousness. Frontiers in psychology. 2018;9:1028. Epub 2018/07/18. doi: 10.3389/fpsyg.2018.01028 30013493 PMC6036510

[31] Van Dam NT, Targett J, Burger A, Davies JN, Galante J. Development and Validation of the Inventory of Unusual Experiences (IUE) for the Assessment of Meditation-related Experiences. Psyarxiv Preprint (https://osfio/preprints/psyarxiv/3ht95). 2023.

[32] Taves A. Mystical and Other Alterations in Sense of Self: An Expanded Framework for Studying Nonordinary Experiences. Perspectives on psychological science: a journal of the Association for Psychological Science. 2020;15(3):669–90. Epub 2020/02/14. doi: 10.1177/1745691619895047 . 32053465

[33] Lindahl JR, Palitsky R, Cooper DJ, Britton WB. The roles and impacts of worldviews in the context of meditation-related challenges. Transcultural psychiatry. 2022:13634615221128679. doi: 10.1177/13634615221128679 36476189 PMC11292974

[34] Lindahl JR, Cooper DJ, Fisher NE, Kirmayer LJ, Britton WB. Progress or Pathology? Differential Diagnosis and Intervention Criteria for Meditation-Related Challenges: Perspectives From Buddhist Meditation Teachers and Practitioners. Frontiers in psychology. 2020;11. doi: 10.3389/fpsyg.2020.01905 32849115 PMC7403193

[35] Cooper DJ, Lindahl JR, Palitsky R, Britton WB. “Like a Vibration Cascading through the Body”: Energy-Like Somatic Experiences Reported by Western Buddhist Meditators. Religions. 2021;12(12). doi: 10.3390/rel12121042

[36] Britton WB, Lindahl JR, Cooper DJ, Canby NK, Palitsky R. Defining and Measuring Meditation-Related Adverse Effects in Mindfulness-Based Programs. Clinical Psychological Science. 2021;9(6):1185–204. Epub 2022/02/18. doi: 10.1177/2167702621996340 35174010 PMC8845498

[37] Galante J, Dufour G, Benton A, Howarth E, Vainre M, Croudace TJ, et al. Protocol for the Mindful Student Study: a randomised controlled trial of the provision of a mindfulness intervention to support university students’ well-being and resilience to stress. BMJ open [Internet]. 2016; 6. Available from: doi: 10.1136/bmjopen-2016-012300 28186934 PMC5129000

[38] Galante J, Dufour G, Vainre M, Wagner AP, Stochl J, Benton A, et al. A mindfulness-based intervention to increase resilience to stress in university students (the Mindful Student Study): a pragmatic randomised controlled trial. The Lancet Public Health. 2018;3(2):e72–e81. Epub 2018/02/10. doi: 10.1016/S2468-2667(17)30231-1 29422189 PMC5813792

[39] Galante J, Stochl J, Dufour G, Vainre M, Wagner AP, Jones PB. Effectiveness of providing university students with a mindfulness-based intervention to increase resilience to stress: one-year follow-up of a pragmatic randomised controlled trial. Journal of Epidemiology and Community Health. 2020;75(2):151–60. Epub 2020/09/12. doi: 10.1136/jech-2020-214390 32913130 PMC7116569

[40] Turner L, Galante J, Vainre M, Stochl J, Dufour G, Jones P. Immune dysregulation among students exposed to exam stress and its mitigation by mindfulness training: findings from an exploratory randomised trial. Scientific reports. 2020;10(1):1–11. doi: 10.17863/CAM.49516 32242145 PMC7118166

[41] Bóo SJM, Childs‐Fegredo J, Cooney S, Datta B, Dufour G, Jones PB, et al. A follow‐up study to a randomised control trial to investigate the perceived impact of mindfulness on academic performance in university students. Couns Psychother Res. 2019;20(2):286–301. doi: 10.1002/capr.12282

[42] Williams M, Penman D. Mindfulness: a practical guide to finding peace in a frantic world. London: Hachette UK; 2011.

[43] Bodmer I, Dittrich A, Lamparter D. Aussergewöhnliche Bewusstseinszustände—Ihre gemeinsame Struktur und Messung [Altered states of consciousness—Their common structure and assessment]. In: Hofmann A, Leuner H, editors. Welten des Bewusstseins: Experimentelle Psychologie, Neurobiologie und Chemie Bd 3. Berlin, Germany: VWB; 1994.

[44] Studerus E, Gamma A, Vollenweider FX. Psychometric evaluation of the altered states of consciousness rating scale (OAV). PloS one. 2010;5(8):e12412. Epub 2010/09/09. doi: 10.1371/journal.pone.0012412 20824211 PMC2930851

[45] Gouzoulis-Mayfrank E, Habermeyer E, Hermle L, Steinmeyer A, Kunert H, Sass H, et al. Hallucinogenic drug induced states resemble acute endogenous psychoses: results of an empirical study Mind machines: a controlled study on the effects of electromagnetic and optic-acoustic stimulation on general well-being, electrodermal activity, and exceptional psychological experiences. Eur Psychiatry. 1998;13(8):399–406. Epub 1998/12/01 1998/12/16. 19698655 10.1016/S0924-9338(99)80686-5

[46] Walach H, Käseberg E. Mind machines: a controlled study on the effects of electromagnetic and optic-acoustic stimulation on general well-being, electrodermal activity, and exceptional psychological experiences. Behavioral medicine (Washington, DC). 1998;24(3):107–14. Epub 1998/12/16. doi: 10.1080/08964289809596388 . 9850804

[47] Kjellgren A, Sundequist U, Sundholm U, Norlander T, Archer T. ALTERED CONSCIOUSNESS IN FLOTATION-REST AND CHAMBER-REST: EXPERIENCE OF EXPERIMENTAL PAIN AND SUBJECTIVE STRESS. Social Behavior and Personality: an international journal. 2004;32(2):103–15. doi: 10.2224/sbp.2004.32.2.103

[48] Muller F, Kraus E, Holze F, Becker A, Ley L, Schmid Y, et al. Flashback phenomena after administration of LSD and psilocybin in controlled studies with healthy participants. Psychopharmacology (Berl). 2022. Epub 2022/01/26. doi: 10.1007/s00213-022-06066-z . 35076721 PMC9166883

[49] Connell J, Barkham M, Mellor-Clark J. The effectiveness of UK student counselling services: an analysis using the CORE System. British Journal of Guidance & Counselling. 2008;36(1):1–18. doi: 10.1080/03069880701715655

[50] Core System Group. CORE system user manual15/09/2015]; (15/09/2015). http://www.coreims.co.uk/index.html.

[51] Mihaylova B, Briggs A, O’Hagan A, Thompson SG. Review of statistical methods for analysing healthcare resources and costs. Health Econ. 2011;20(8):897–916. doi: 10.1002/hec.1653 . 20799344 PMC3470917

[52] Belotti F, Deb P, Manning WG, Norton EC. Twopm: Two-Part Models. The Stata Journal. 2015;15(1):3–20. doi: 10.1177/1536867X1501500102

[53] Yoav B, Daniel Y. The control of the false discovery rate in multiple testing under dependency. The Annals of Statistics. 2001;29(4):1165–88. doi: 10.1214/aos/1013699998

[54] Cohen J. Statistical power analysis for the behavioral sciences. 2nd ed. Hillsdale, NJ: Lawrence Erlbaum Associates; 1988.

[55] Lachenbruch PA. Comparisons of two-part models with competitors. Statistics in medicine. 2001;20(8):1215–34. doi: 10.1002/sim.790 11304737

[56] Sucala M, David D. Mindful about Time in a Fast Forward World. The Effects of Mindfulness Exercise on Time Perception. Transylvanian Journal of Psychology. 2013.

[57] Berkovich-Ohana A, Glicksohn J. Meditation, Absorption, Transcendent Experience, and Affect: Tying It All Together Via the Consciousness State Space (CSS) Model. Mindfulness. 2017;8(1):68–77. doi: 10.1007/s12671-015-0481-9

[58] Segal ZV, Williams JMG, Teasdale JD. Mindfulness-Based Cognitive Therapy for Depression. 2nd edition ed. New York: Guilford Publications; 2013.

[59] Hanley AW, Dorjee D, Garland EL. Mindfulness training encourages self-transcendent states via decentering. Psychology of Consciousness: Theory, Research, and Practice. 2020:No-Pagination Specified-No Pagination Specified.

[60] Bowden D, Gaudry C, An SC, Gruzelier J. A comparative randomised controlled trial of the effects of brain wave vibration training, iyengar yoga, and mindfulness on mood, well-being, and salivary cortisol. Evidence-based complementary and alternative medicine: eCAM. 2012;2012:234713. Epub 2012/01/05. doi: 10.1155/2012/234713 22216054 PMC3246835

[61] Ian W II. The modified Tellegen absorption scale: A clearer window on the structure and meaning of absorption. The American journal of clinical hypnosis. 2006;48(4):317.

[62] Althouse AD. Adjust for Multiple Comparisons? It’s Not That Simple. Ann Thorac Surg. 2016;101(5):1644–5. Epub 2016/04/24. doi: 10.1016/j.athoracsur.2015.11.024 . 27106412

[63] Baer R, Crane C, Montero-Marin J, Phillips A, Taylor L, Tickell A, et al. Frequency of Self-reported Unpleasant Events and Harm in a Mindfulness-Based Program in Two General Population Samples. Mindfulness. 2020. doi: 10.1007/s12671-020-01547-8 33747251 PMC7920887

[64] Dambrun M. When the dissolution of perceived body boundaries elicits happiness: The effect of selflessness induced by a body scan meditation. Consciousness and cognition. 2016;46:89–98. Epub 2016/10/21. doi: 10.1016/j.concog.2016.09.013 . 27684609

[65] Lindahl JR, Fisher NE, Cooper DJ, Rosen RK, Britton WB. The varieties of contemplative experience: A mixed-methods study of meditation-related challenges in Western Buddhists. PloS one. 2017;12(5):e0176239. Epub 2017/05/26. doi: 10.1371/journal.pone.0176239 28542181 PMC5443484

[66] Burrows L. Safeguarding Mindfulness Meditation for Vulnerable College Students. Mindfulness [Internet]. 2016; 7:[284–5 pp.].

[67] Britton WB. Can mindfulness be too much of a good thing? The value of a middle way. Current opinion in psychology. 2019;28:159–65. Epub 2019/02/02. doi: 10.1016/j.copsyc.2018.12.011 . 30708288 PMC6612475

[68] Castillo RJ. Depersonalization and Meditation. Psychiatry. 1990;53(2):158–68. doi: 10.1080/00332747.1990.11024497 2191357

[69] Baer R, Crane C, Miller E, Kuyken W. Doing no harm in mindfulness-based programs: Conceptual issues and empirical findings. Clinical psychology review. 2019. Epub 2019/01/15. doi: 10.1016/j.cpr.2019.01.001 . 30638824 PMC6575147

[70] Langer ÁI, Cangas AJ, Gallego J. Mindfulness-Based Intervention on Distressing Hallucination-Like Experiences in a Nonclinical Sample. Behaviour Change. 2012;27(03):176–83. doi: 10.1375/bech.27.3.176

