# Supplementary material for: Altered states of consciousness caused by a mindfulness-based programme up to a year later: Results from a randomised controlled trial
Source: PLoS One. 2024 Jul 17;19(7):e0305928. doi: 10.1371/journal.pone.0305928 (PMC11253948; doi:10.1371/journal.pone.0305928)

Altered states of consciousness caused by a mindfulness-based programme up to a year later: results from a randomised controlled trial

Julieta Galante*, Jesus Montero-Marin*, Maris Vainre, Géraldine Dufour, Javier García-Campayo, Peter B Jones

* Joint first authors

S1 Appendix: Supplemental Materials

Contents

Adapted OAV used in this study 2
Supplemental Tables 6
Supplemental Figures 20

Adapted OAV used in this study

Different states of consciousness

Below you will find a number of statements. Next to each statement is a line with the endpoints "No, not more than usual" and "Yes, much more than usual". The line represents something like a thermometer that will be used to assess changes from your normal waking consciousness (i.e. how you feel on an everyday basis).

Please rate to what extent the statements reflect any unusual experiences you may have had **in the last YEAR**, by moving the slider next to the statements. Note that your normal waking consciousness corresponds to a mark at the very left end of the scale, i.e.: "No, not more than usual". The further to the right you move the slide, the more unusual the experience.

Do **NOT** count any experiences you had under the influence of **alcohol or drugs**. Please complete the scale even if you haven't had any altered or unusual experiences. Your response will still be really useful to us.

	No, not more than usual	Yes, much more than usual
I had the feeling of being connected to a higher power		
I felt like a puppet (my actions being controlled by someone else)		
I felt boundless joy		
I saw patterns in complete darkness or with closed eyes		
Everything seemed to come together as one		
Noises seemed to influence what I saw		
I saw colours before me in total darkness or with closed eyes		
The shapes of things seemed to change by sounds and noises		
It seemed to me as though I did not have a body anymore		

I had difficulty making even the smallest decision

I was afraid that the state I was in would last forever

I saw light or flashes of light in total darkness or with closed eyes

It seemed to me that my environment and I were one

I had difficulty in distinguishing important from unimportant things

I saw scenes rolling by in total darkness or with my eyes closed

I experienced a touch of eternity

Conflicts and contradictions seemed to dissolve

I was afraid without being able to say exactly why

I experienced everything as terrifyingly distorted

I experienced my surroundings as strange and weird

Everyday things gained a special meaning

I felt as though I were paralysed

I felt very profound

I experienced past, present and future as a oneness

I felt threatened

I had the feeling of being outside of my body

I felt as though I were floating

I felt isolated from everything and everyone

I was not able to complete a thought, my thought repeatedly became disconnected

I gained clarity into connections that puzzled me before

I could see pictures from my past or fantasies extremely clearly

The colours of things seemed to be changed by sounds and noises

I had very original thoughts

I had the feeling that I no longer had a will of my own

I experienced a kind of awe

My imagination was extremely vivid

I experienced a profound peace in myself

I had the feeling something horrible would happen

I experienced an all-embracing love

My experience had religious aspects

Things around me had a new strange meaning for me

Objects around me engaged me emotionally much more than usual

Other aspects

Is there any other aspect about an unusual state you have experienced in the last year that you would like to tell us about?

Meditation

If you have been practising meditation in the last year, did any of the unusual states you have experienced take place while you were meditating? Select all that apply:

- ☐ I haven't meditated in the last year
- ☐ I have meditated in the last year, but none of these states happened while I was meditating
- ☐ Prefer not to answer
- ☐ [All the states selected by the participant in the questionnaire will be displayed here as options]

Flashback

Did any of the unusual states you have experienced occur during a "flashback" as a result of psychoactive drug use? Select all that apply:

- ☐ None apply
- ☐ Prefer not to answer
- ☐ [All the states selected by the participant in the questionnaire will be displayed here as options]

Supplemental Tables

Supplemental Table 1. Regression model to assess baseline variables predicting OAV completion among randomised participants.

	Odds Ratios	Standard Error	P value
Arm - Intervention	1.04	0.19	0.84
Cohort 1	1.21	0.19	0.32
Age	1.04	0.02	0.1
Gender – Men	0.68	0.21	0.06
Ethnicity – White	2.25	0.27	0.003
Nationality* – International	0.95	0.31	0.88
Disability – Yes	1.15	0.29	0.62
Degree – Undergraduate	0.98	0.25	0.93
Last Year	0.48	0.21	0.0005
Previous meditation**	1.02	0.31	0.94
Baseline CORE-OM	0.57	0.29	0.05
Baseline WEMWBS	0.97	0.02	0.06

* Inferred from whether participants paid their study fees according to UK/European Union, or overseas rates.
** Spent in total more than 10 hours meditating in the past, or completed an eight-week mindfulness course.
Abbreviations: CORE-OM: Clinical Outcomes in Routine Evaluation Outcome Measure; WEMWBS: Warwick-Edinburgh Mental Wellbeing Scale.

Supplemental Table 2. Regression model to assess baseline variables predicting OAV completion among those who completed the main part of the one-year follow-up questionnaire.

	Odds Ratios	Standard Error	P value
Arm - Intervention	1.05	0.25	0.85
Cohort 1	1.35	0.25	0.23
Age	1.08	0.04	0.04
Gender – Men	0.85	0.26	0.55
Ethnicity – White	2.25	0.31	0.01
Nationality* – International	0.51	0.35	0.06
Disability – Yes	1.2	0.38	0.63
Degree – Undergraduate	1.26	0.34	0.49
Last Year	0.92	0.27	0.78
Previous meditation**	1.05	0.4	0.9
Baseline CORE-OM	0.5	0.39	0.08
Baseline WEMWBS	0.96	0.02	0.1

* Inferred from whether participants paid their study fees according to UK/European Union, or overseas rates.
** Spent in total more than 10 hours meditating in the past, or completed an eight-week mindfulness course.
Abbreviations: CORE-OM: Clinical Outcomes in Routine Evaluation Outcome Measure; WEMWBS: Warwick-Edinburgh Mental Wellbeing Scale.

Supplemental Table 3. Logit Probability Model for Experiencing Altered States of Consciousness (ASC) by trial arm.

ASC dimensions		MSS		SAU				
	**Category**	**n**	**%**	**n**	**%**	**OR**	**95% CI**	**p**
Experience of Unity	zero	39	42.4	53	57.6			
	>zero	61	58.1	44	41.9	2.01	1.09, 3.67	0.024
Spiritual Experience	zero	49	49.0	54	55.7			
	>zero	51	51.0	43	44.3	1.31	0.73, 2.36	0.368
Blissful State	zero	25	25.0	32	33.0			
	>zero	75	75.0	65	67.0	1.35	0.70, 2.59	0.360
Insightfulness	zero	43	43.0	47	48.5			
	>zero	57	57.0	50	51.5	1.28	0.70, 2.32	0.417
Disembodiment	zero	58	58.0	72	74.2			
	>zero	42	42.0	25	25.8	2.29	1.23, 4.26	0.009
Impaired Control and Cognition	zero	39	39.0	33	34.0			
	>zero	61	61.0	64	66.0	0.79	0.43, 1.47	0.452
Anxiety	zero	46	46.0	35	36.1			
	>zero	54	54.0	62	63.9	0.68	0.37, 1.24	0.200
Complex Imagery	zero	59	59.0	59	60.8			
	>zero	41	41.0	38	39.2	1.09	0.61, 1.95	0.758
Elementary Imagery	zero	56	56.0	61	62.9			
	>zero	44	44.0	36	37.1	1.75	0.97, 3.18	0.063
Audio-Visual Synesthesia	zero	76	76.0	86	88.7			
	>zero	24	24.0	11	11.3	2.41	1.10, 5.27	0.028
Changed Meaning of Percepts	zero	48	48.0	60	61.9			
	>zero	52	52.0	37	38.1	1.70	0.94, 3.10	0.084

MSS: mindfulness skills for students. SAU: support as usual. n: raw frequencies; raw %: percentages. OR: odds ratio of experiencing a value >0; 95% CI: 95% confidence interval. The model controlled for the ASC experienced during flashbacks, as well as age, gender and cohort (design variables). p: p-value associated with the Wald test using robust standard errors.

Supplemental Table 4. GLM Probability Model for Experiencing Altered States of Consciousness (ASC) by trial arm.

	**MSS**	**SAU**			
**ASC dimension**	**M (SD)**	**M (SD)**	**Coefficient**	**95% CI**	**p**
Experience of Unity<br>n (MSS, SAU) = 61, 44	18.03 (19.28)	14.04 (17.45)	0.51	(0.04, 0.97)	.032
Spiritual Experience<br>n (MSS, SAU) = 51, 43	22.82 (20.16)	19.44 (21.30)	0.19	(-0.21, 0.58)	.352
Blissful State<br>n (MSS, SAU) = 75, 67	27.17 (26.78)	21.77 (20.74)	0.27	(-0.07, 0.60)	.122
Insightfulness<br>n (MSS, SAU) = 57, 50	19.42 (20.67)	19.35 (18.90)	0.09	(-0.31, 0.48)	.675
Disembodiment<br>n (MSS, SAU) = 42, 26	20.11 (22.83)	14.62 (14.52)	0.26	(-0.25, 0.77)	.313
Impaired Control and Cognition<br>n (MSS, SAU) = 62, 68	14.73 (15.88)	15.53 (17.01)	-0.09	(-0.48, 0.29)	.641
Anxiety<br>n (MSS, SAU) = 54, 65	15.54 (19.30)	15.72 (18.22)	-0.09	(-0.49, 0.31)	.664
Complex Imagery<br>n (MSS, SAU) = 40, 39	18.71 (19.86)	21.13 (19.92)	-0.11	(-0.56, 0.35)	.645
Elementary Imagery<br>n (MSS, SAU) = 46, 36	21.93 (21.96)	15.86 (20.55)	0.35	(-0.17, 0.86)	.185
Audio-Visual Synesthesia<br>n (MSS, SAU) = 25, 12	11.08 (12.83)	12.28 (19.50)	-0.29	(-1.27, 0.70)	.569
Changed Meaning of Percepts<br>n (MSS, SAU) = 54, 39	18.44 (20.80)	16.81 (16.74)	0.11	(-0.34, 0.55)	.639

MSS: mindfulness skills for students. SAU: support as usual. M (SD): raw mean (standard deviation). Coefficient: unstandardized coefficient for the Generalized Linear Model (GLM) with the log link and gamma distribution part of the two-part model. 95% CI: 95% confidence interval. p: p-value associated with the Wald test contrast using robust standard errors.

Supplemental Table 5. Logit Probability Model for Experiencing Altered States of Consciousness (ASCs) with (formal) Mindfulness Practice as a Predictor.

ASC dimensions	Category	OR	95% CI	p
Experience of Unity	zero			
	>zero	1.02	1.01, 1.04	0.007
Spiritual Experience	zero			
	>zero	1.01	0.99, 1.02	0.397
Blissful State	zero			
	>zero	1.02	1.01, 1.04	0.007
Insightfulness	zero			
	>zero	1.01	1.00, 1.03	0.047
Disembodiment	zero			
	>zero	1.02	1.01, 1.04	<.001
Impaired Control and Cognition	zero			
	>zero	0.99	0.98, 1.00	0.045
Anxiety	zero			
	>zero	0.98	0.97, 1.00	0.019
Complex Imagery	zero			
	>zero	1.01	0.99, 1.02	0.252
Elementary Imagery	zero			
	>zero	1.01	0.99, 1.02	0.407
Audio-Visual Synesthesia	zero			
	>zero	1.00	0.99, 1.02	0.549
Changed Meaning of Percepts	zero			
	>zero	1.01	0.99, 1.02	0.169

OR: odds ratio of experiencing a value >0; 95% CI: 95% confidence interval; p: p-value associated with the Wald test using robust standard errors. The model controlled for the ASCs experienced during flashbacks, as well as age, gender and cohort (design variables).

Supplemental Table 6. GLM Probability Model for Experiencing Altered States of Consciousness (ASCs) with (formal) Mindfulness Practice as a Predictor.

ASC dimension	Coefficient	95% CI	p
Experience of Unity	0.01	(0.01, 0.02)	.001
Spiritual Experience	0.01	(0.01, 0.02)	<.001
Blissful State	0.01	(0.01, 0.02)	<.001
Insightfulness	0.01	(0.0001, 0.01)	.048
Disembodiment	0.01	(-0.001, 0.01)	.122
Impaired Control and Cognition	-0.004	(-0.01, 0.002)	.155
Anxiety	0.0002	(-0.007, 0.007)	.960
Complex Imagery	0.001	(-0.01, 0.01)	.909
Elementary Imagery	0.004	(-0.004, 0.01)	.343
Audio-Visual Synesthesia	0.02	(-0.0002, 0.04)	.052
Changed Meaning of Percepts	0.01	(0.002, 0.02)	.017

Coefficient: unstandardized coefficient for the Generalized Linear Model (GLM) with the log link and gamma distribution part of the two-part model. 95% CI: 95% confidence interval. p: p-value associated with the Wald test contrast using robust standard errors. The model controlled for the ASCs experienced during flashbacks, as well as age, gender and cohort (design variables).

Table 7. Joint Model (Zero and Non-Zero Parts) for Experiencing Altered States of Consciousness (ASC) with (informal) Mindfulness Practice as a Predictor.

ASC dimension	B	SE	95% CI	χ^2 (df)	p
Experience of Unity	5.45	2.50	0.56, 10.35	6.17 (2)	.045
Spiritual Experience	3.32	2.05	-0.69, 7.33	3.31 (2)	.191
Blissful State	8.87	3.40	2.22, 15.53	6.90 (2)	.032
Insightfulness	4.53	2.42	-0.21, 9.28	4.08 (2)	.130
Disembodiment	2.78	2.05	-0.24, 6.79	1.96 (2)	.375
Impaired Control and Cognition	-2.63	1.61	-5.79, 0.53	4.04 (2)	.133
Anxiety	-2.56	1.66	-5.81, 0.70	3.69 (2)	.158
Complex Imagery	0.65	1.87	-3.00, 4.31	0.12 (2)	.940
Elementary Imagery	-0.03	2.32	-4.58, 4.53	0.35 (2)	.840
Audio-Visual Synesthesia	-0.96	1.13	-3.18, 1.26	1.13 (2)	.568
Changed Meaning of Percepts	4.16	2.33	-0.41, 8.73	4.78 (2)	.092

B: marginal effects. SE: (Robust) Standard Error. 95% CI: 95% Confidence Interval for the marginal effects. p: p-value associated with the joint statistical test of significance including the parameters of the two-part model (logit in the first part (zero vs. >zero), and GLM with a log link and gamma distribution in the second part (>zero)). Models included the following predictors: informal mindfulness practice, ASC as a consequence of flashbacks, as well as age, sex, and cohort (design variables). The significant relationships were not longer significant when the Benjamini-Hochberg correction was applied to correct for multiple comparisons.

Supplemental Table 8. Logit Probability Model for Experiencing Altered States of Consciousness (ASCs) with (informal) Mindfulness Practice as a Predictor.

ASC dimensions	Category	OR	95% CI	p
Experience of Unity	zero			
	>zero	1.78	1.00, 3.20	0.049
Spiritual Experience	zero			
	>zero	1.54	0.92, 2.56	0.097
Blissful State	zero			
	>zero	1.84	0.94, 3.61	0.076
Insightfulness	zero			
	>zero	1.46	0.87, 2.46	0.148
Disembodiment	zero			
	>zero	1.23	0.76, 2.01	0.387
Impaired Control and Cognition	zero			
	>zero	0.91	0.55, 1.52	0.739
Anxiety	zero			
	>zero	0.68	0.41, 1.12	0.126
Complex Imagery	zero			
	>zero	1.06	0.65, 1.73	0.805
Elementary Imagery	zero			
	>zero	1.14	0.69, 1.86	0.620
Audio-Visual Synesthesia	zero			
	>zero	0.97	0.54, 1.75	0.917
Changed Meaning of Percepts	zero			
	>zero	1.54	0.93, 2.54	0.093

OR: odds ratio of experiencing a value >0; 95% CI: 95% confidence interval; p: p-value associated with the Wald test using robust standard errors. The model controlled for the ASCs experienced during flashbacks, as well as age, gender and cohort (design variables).

Supplemental Table 9. GLM Probability Model for Experiencing Altered States of Consciousness (ASCs) with (informal) Mindfulness Practice as a Predictor.

ASC dimension	Coefficient	95% CI	p
Experience of Unity	0.29	(-0.07, 0.65)	.113
Spiritual Experience	0.08	(-0.20, 0.35)	.589
Blissful State	0.28	(-0.002, 0.56)	.052
Insightfulness	0.27	(-0.06, 0.60)	.106
Disembodiment	0.22	(-0.23, 0.67)	.331
Impaired Control and Cognition	-0.31	(-0.61, -0.001)	.049
Anxiety	-0.39	(-0.52, 0.18)	.333
Complex Imagery	0.05	(-0.34, 0.45)	.786
Elementary Imagery	-0.06	(-0.43, 0.30)	.729
Audio-Visual Synesthesia	-0.30	(-0.85, 0.25)	.288
Changed Meaning of Percepts	0.24	(-0.11, 0.60)	.183

Coefficient: unstandardized coefficient for the Generalized Linear Model (GLM) with the log link and gamma distribution part of the two-part model. 95% CI: 95% confidence interval. p: p-value associated with the Wald test contrast using robust standard errors. The model controlled for the ASCs experienced during flashbacks, as well as age, gender and cohort (design variables).

Supplemental Table 10. Altered states of consciousness that were reported to happen during meditation

ASCs	N=73	
	Freq.	%
Experience of Unity	31	42.5
Spiritual Experience	14	19.2
Blissful State	34	46.6
Insightfulness	18	24.7
Disembodiment	21	28.8
Impaired Control and Cognition	11	15.1
Anxiety	8	11.0
Complex Imagery	11	15.1
Elementary Imagery	13	17.8
Audio-Visual Synesthesia	3	4.1
Changed Meaning of Percepts	15	20.6

ASCs: altered states of consciousness. Due to an error during data collection, only those participants who reported having meditated in the prior six months saw the question about whether ASCs happened during meditation, so we base the analysis of the proportion of ASCs that happened during meditation on this sub-sample (N=73; MSS=59; SAU=14). MSS: mindfulness skills for students. SAU: support as usual. Freq.: frequencies; %: percentages.

Supplemental Table 11. ASC experiences that were reported to happen due to flasbacks

		MSS		SAU		Total	
**ASC dimensions**	**value**	**n**	**%**	**n**	**%**	**n**	**%**
Experience of Unity	no	98	98.0	91	93.8	189	95.9
	yes	2	2.0	6	6.2	8	4.1
Spiritual Experience	no	97	97.0	95	97.9	192	97.5
	yes	3	3.0	2	2.1	5	2.5
Blissful State	no	97	97.0	94	96.9	191	97.0
	yes	3	3.0	3	3.1	6	3.0
Insightfulness	no	99	99.0	93	95.9	192	97.5
	yes	1	1.0	4	4.1	5	2.5
Disembodiment	no	100	100	95	97.9	195	99.0
	yes	0	0.0	2	2.1	2	1.0
Impaired Control and Cognition	no	98	98.0	94	96.9	192	97.5
	yes	2	2.0	3	3.1	5	2.5
Anxiety	no	98	98.0	93	95.9	191	97.0
	yes	2	2.0	4	4.1	6	3.0
Complex Imagery	no	100	100	96	99.0	196	99.5
	yes	0	0.0	1	1.0	1	0.5
Elementary Imagery	no	100	100	95	97.9	195	99.0
	yes	0	0.0	2	2.1	2	1.0
Audio-Visual Synesthesia	no	100	100	95	97.9	195	99.0
	yes	0	0.0	2	2.1	2	1.0
Changed Meaning of Percepts	no	98	98.0	95	97.9	193	98.0
	yes	2	2.0	2	2.1	4	2.0

ASC: altered states of consciousness. MSS: mindfulness skills for students. SAU: support as usual. n: frequencies; %: percentages.

Supplemental Table 12. Joint Model (Zero and Non-Zero Parts) for Experiencing Altered States of Consciousness (ASC) with baseline sociodemographic characteristics as potential predictors

**ASC dimension**	**B**	**SE**	**95% CI**	**χ^2 (df)**	**p**
Experience of Unity					
Age (years)	-0.16	0.19	-0.52, 0.21	4.64 (2)	.098
Sex (male)	-2.08	2.28	-6.54, 2.39	3.02 (2)	.221
Previous meditation (hours)	0.16	0.07	0.02, 0.30	6.68 (2)	.035†
Disability (yes)	-0.20	3.10	-6.27, 5.87	0.04 (2)	.982
Nationality (international)	4.68	4.23	-3.61, 12.97	1.70 (2)	.427
Ethnicity (other than white)	6.00	4.61	-3.03, 15.02	2.48 (2)	.290
Spirituality	1.52	1.80	-2.01, 5.06	1.00 (2)	.606
Baseline distress	4.92	2.61	-0.19, 10.04	4.54 (2)	.103
Spiritual Experience					
Age (years)	-0.20	0.21	-0.62, 0.22	3.25 (2)	.197
Sex (male)	-1.02	2.74	-6.38, 4.35	0.53 (2)	.768
Previous meditation (hours)	0.10	0.08	-0.06, 0.26	1.49 (2)	.475
Disability (yes)	2.56	3.62	-4.54, 9.66	6.07 (2)	.048†
Nationality (international)	-2.16	2.98	-7.99, 3.68	0.50 (2)	.778
Ethnicity (other than white)	6.01	4.29	-2.39, 14.41	2.81 (2)	.246
Spirituality	4.75	1.78	1.25, 8.24	7.85 (2)	.020†
Baseline distress	2.07	2.20	-2.24, 6.38	0.90 (2)	.637
Blissful State					
Age (years)	-0.31	0.30	-0.89, 0.26	2.61 (2)	.271
Sex (male)	-2.78	3.39	-9.42, 3.87	1.25 (2)	.537
Previous meditation (hours)	0.30	0.11	0.08, 0.52	7.27 (2)	.026†
Disability (yes)	3.49	5.20	-6.70, 13.69	0.50 (2)	.777
Nationality (international)	2.42	5.82	-8.99, 13.82	2.56 (2)	.278
Ethnicity (other than white)	7.48	5.49	-3.28, 18.24	4.79 (2)	.091
Spirituality	4.40	2.29	-0.10, 8.90	4.08 (2)	.130
Baseline distress	5.25	2.91	-0.45, 10.96	4.46 (2)	.107
Insightfulness					
Age (years)	-0.14	0.22	-0.57, 0.28	2.31 (2)	.315
Sex (male)	0.39	2.56	-4.63, 5.40	3.11 (2)	.211
Previous meditation (hours)	-0.04	0.07	-0.19, 0.10	5.92 (2)	.052
Disability (yes)	1.32	3.34	-5.23, 7.87	1.15 (2)	.564
Nationality (international)	3.62	4.31	-4.83, 12.08	8.64 (2)	.013†
Ethnicity (other than white)	8.06	4.65	-1.05, 17.18	5.41 (2)	.067
Spirituality	-0.64	2.03	-4.61, 3.33	1.38 (2)	.502
Baseline distress	2.23	2.28	-2.24, 6.70	1.14 (2)	.565
Disembodiment					
Age (years)	-0.26	0.18	-0.60, 0.08	11.02 (2)	.004
Sex (male)	-0.22	2.32	-4.77, 4.34	0.10 (2)	.949
Previous meditation (hours)	0.02	0.05	-0.08, 0.11	0.14 (2)	.932
Disability (yes)	-0.15	3.63	-7.28, 6.97	1.82 (2)	.402
Nationality (international)	2.71	3.14	-3.45, 8.86	1.34 (2)	.513
Ethnicity (other than white)	2.74	3.60	-4.32, 9.79	3.43 (2)	.180
Spirituality	-0.54	1.77	-4.00, 2.92	4.66 (2)	.097
Baseline distress	6.58	2.77	1.15, 12.00	12.39 (2)	.002
Impaired Control and Cognition					
Age (years)	-0.12	0.25	-0.61, 0.36	3.16 (2)	.206
Sex (male)	-1.17	2.19	-5.46, 3.12	0.29 (2)	.864
Previous meditation (hours)	0.04	0.05	-0.06, 0.14	0.69 (2)	.708
Disability (yes)	-0.26	3.03	-6.20, 5.68	0.69 (2)	.708
Nationality (international)	0.53	3.23	-5.80, 6.87	0.43 (2)	.806
Ethnicity (other than white)	-3.30	2.64	-8.47, 1.87	2.97 (2)	.227
Spirituality	2.07	1.73	-1.31, 5.46	1.52 (2)	.468
Baseline distress	6.91	2.59	1.84, 11.99	8.57 (2)	.014†
Anxiety					
Age (years)	-0.08	0.27	-0.60, 0.45	5.11 (2)	.078
Sex (male)	-1.81	2.33	-6.38, 2.76	1.28 (2)	.527
Previous meditation (hours)	-0.002	0.06	-0.11, 0.11	1.21 (2)	.547
Disability (yes)	5.44	3.81	-2.02, 12.91	2.72 (2)	.257
Nationality (international)	1.68	3.98	-6.13, 9.48	0.21 (2)	.902
Ethnicity (other than white)	-3.93	3.09	-9.98, 2.12	1.43 (2)	.489
Spirituality	3.33	1.89	-0.38, 7.04	3.61 (2)	.165
Baseline distress	9.71	2.71	4.40, 15.02	17.07 (2)	<.001

Complex Imagery					
Age (years)	-0.18	0.24	-0.65, 0.29	0.58 (2)	.749
Sex (male)	-0.09	2.41	-4.82, 4.63	0.12 (2)	.940
Previous meditation (hours)	-0.16	0.07	-0.30, -0.02	7.32 (2)	.025†
Disability (yes)	0.90	3.28	-5.53, 7.33	0.09 (2)	.954
Nationality (international)	-0.76	3.34	-7.31, 5.80	0.24 (2)	.885
Ethnicity (other than white)	-2.88	2.75	-8.28, 2.51	1.05 (2)	.593
Spirituality	2.40	1.76	-1.05, 5.85	4.38 (2)	.112
Baseline distress	5.54	2.54	0.56, 10.53	6.15 (2)	.046†
Elementary Imagery					
Age (years)	-0.11	0.26	-0.62, 0.39	1.89 (2)	.389
Sex (male)	0.61	2.46	-4.22, 5.43	2.23 (2)	.328
Previous meditation (hours)	0.05	0.05	-0.05, 0.15	2.49 (2)	.287
Disability (yes)	2.32	3.63	-4.80, 9.43	1.88 (2)	.391
Nationality (international)	3.01	4.80	-6.40, 12.43	8.91 (2)	.012†
Ethnicity (other than white)	-4.00	2.50	-8.89, 0.90	3.25 (2)	.197
Spirituality	0.75	1.86	-2.89, 4.39	0.27 (2)	.973
Baseline distress	5.66	3.01	0.24, 11.55	6.69 (2)	.035†
Audio-Visual Synesthesia					
Age (years)	0.09	0.06	-0.03, 0.20	4.28 (2)	.118
Sex (male)	1.62	1.03	-0.40, 3.64	3.77 (2)	.152
Previous meditation (hours)	-0.001	0.02	-0.05, 0.05	0.78 (2)	.677
Disability (yes)	-0.30	1.42	-3.08, 2.48	0.94 (2)	.626
Nationality (international)	-1.94	0.77	-3.45, -0.42	9.19 (2)	.010†
Ethnicity (other than white)	1.09	1.38	-1.61, 3.78	1.24 (2)	.537
Spirituality	0.94	0.91	-0.84, 2.71	4.72 (2)	.094
Baseline distress	0.37	0.95	-1.49, 2.23	4.10 (2)	.129
Changed Meaning of Percepts					
Age (years)	0.18	0.16	-0.14, 0.50	10.07 (2)	.007†
Sex (male)	-0.44	2.25	-4.85, 3.97	4.01 (2)	.135
Previous meditation (hours)	-0.01	0.06	-0.12, 0.10	1.82 (2)	.403
Disability (yes)	-0.87	3.02	-6.79, 5.04	0.08 (2)	.961
Nationality (international)	2.82	3.64	-4.32, 9.95	2.03 (2)	.362
Ethnicity (other than white)	2.14	3.55	-4.82, 9.11	0.65 (2)	.721
Spirituality	0.97	1.74	-2.44, 4.38	0.51 (2)	.774
Baseline distress	1.94	1.90	-1.78, 5.65	1.20 (2)	.548

B: marginal effects. SE: (Robust) Standard Error. 95% CI: 95% Confidence Interval. p: p-value associated with the joint statistical test of significance including the parameters of the two-part model (logit in the first part (zero vs. >zero), and GLM with a log link and gamma distribution in the second part (>zero)). †This relationship was no longer significant when the Benjamini-Hochberg correction was applied to correct for multiple comparisons.

Supplemental Table 13. Presence (vs zero values) of ASCs as a potential predictor of psychological distress.

	Coefficient	95% CI	p
**Psychological distress**			
Experience of Unity	-0.003	-0.21, 0.21	.981
Spiritual Experience	0.06	-0.13, 0.25	.544
Blissful State	-0.21	-0.43, 0.01	.063
Insightfulness	-0.09	-0.31, 0.12	.397
Disembodiment	0.01	-0.19, 0.20	.938
Impaired Control and Cognition	0.10	-0.10, 0.30	.330
Anxiety	0.32	0.12, 0.52	.002
Complex Imagery	-0.07	-0.26, 0.12	.470
Elementary Imagery	0.001	-0.18, 0.18	.994
Audio-Visual Synesthesia	0.19	-0.04, 0.42	.110
Changed Meaning of Percepts	0.02	-0.17, 0.21	.823

R^2 (adjusted) = 0.08 (F (df) = 2.69 (11); p = .003. Durbin-Watson = 1.88. Tolerance (range) = 0.50, 0.68. The relationship between anxiety and psychological distress at 1-year follow-up was still significant when the Benjamini-Hochberg correction was applied to adjust for multiple comparisons.

Supplemental Figures

Supplemental Figure S1: Experience of Unity

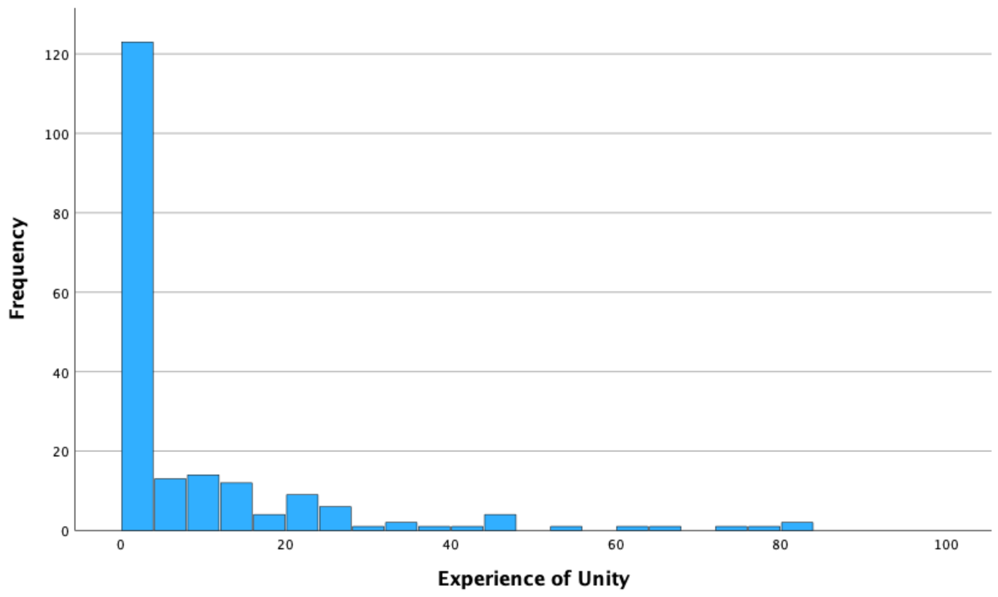

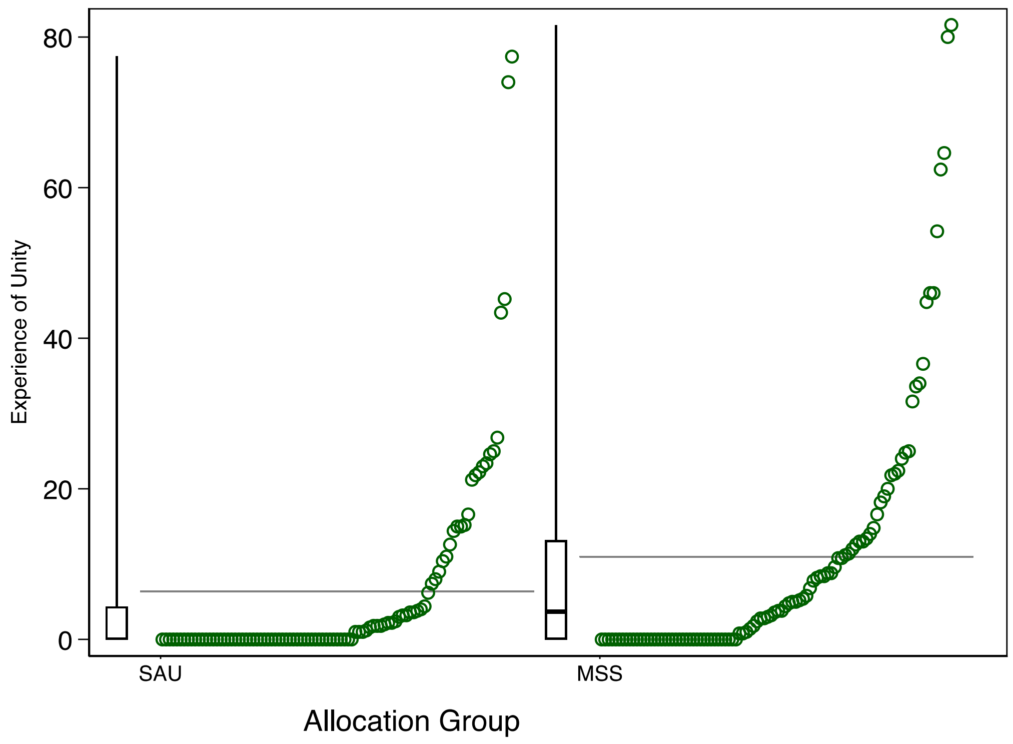

Supplemental Figure S2: Spiritual Experience

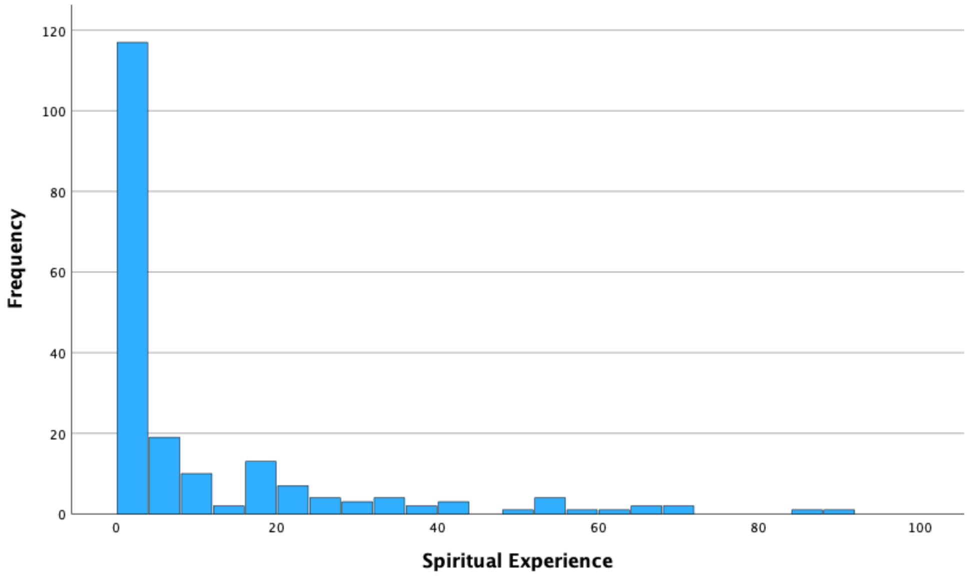

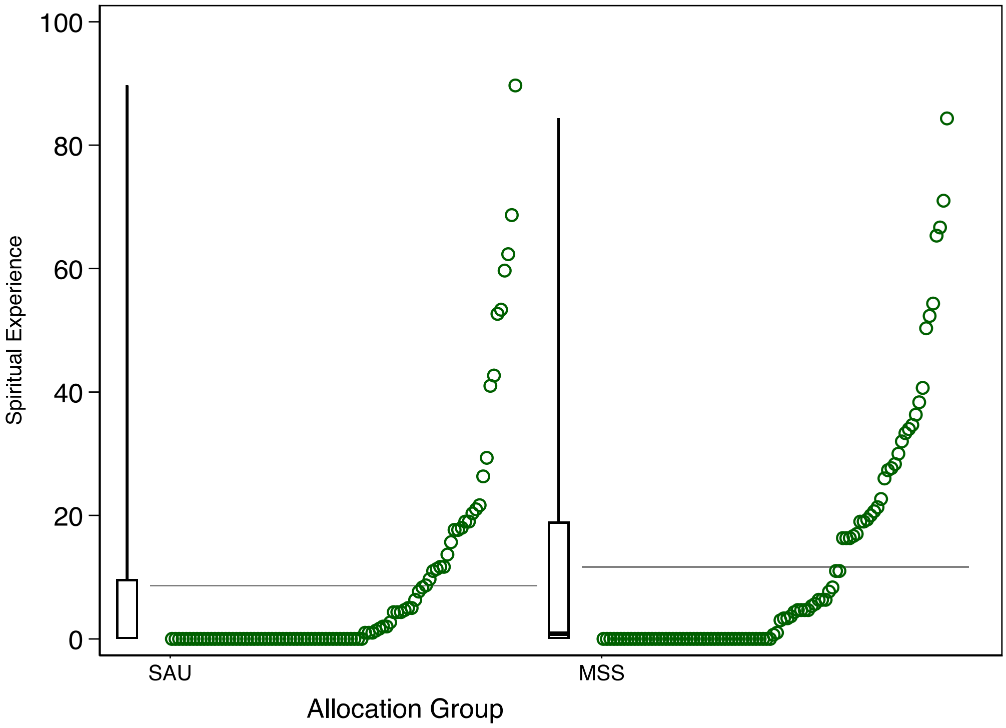

Supplemental Figure S3: Blissful State

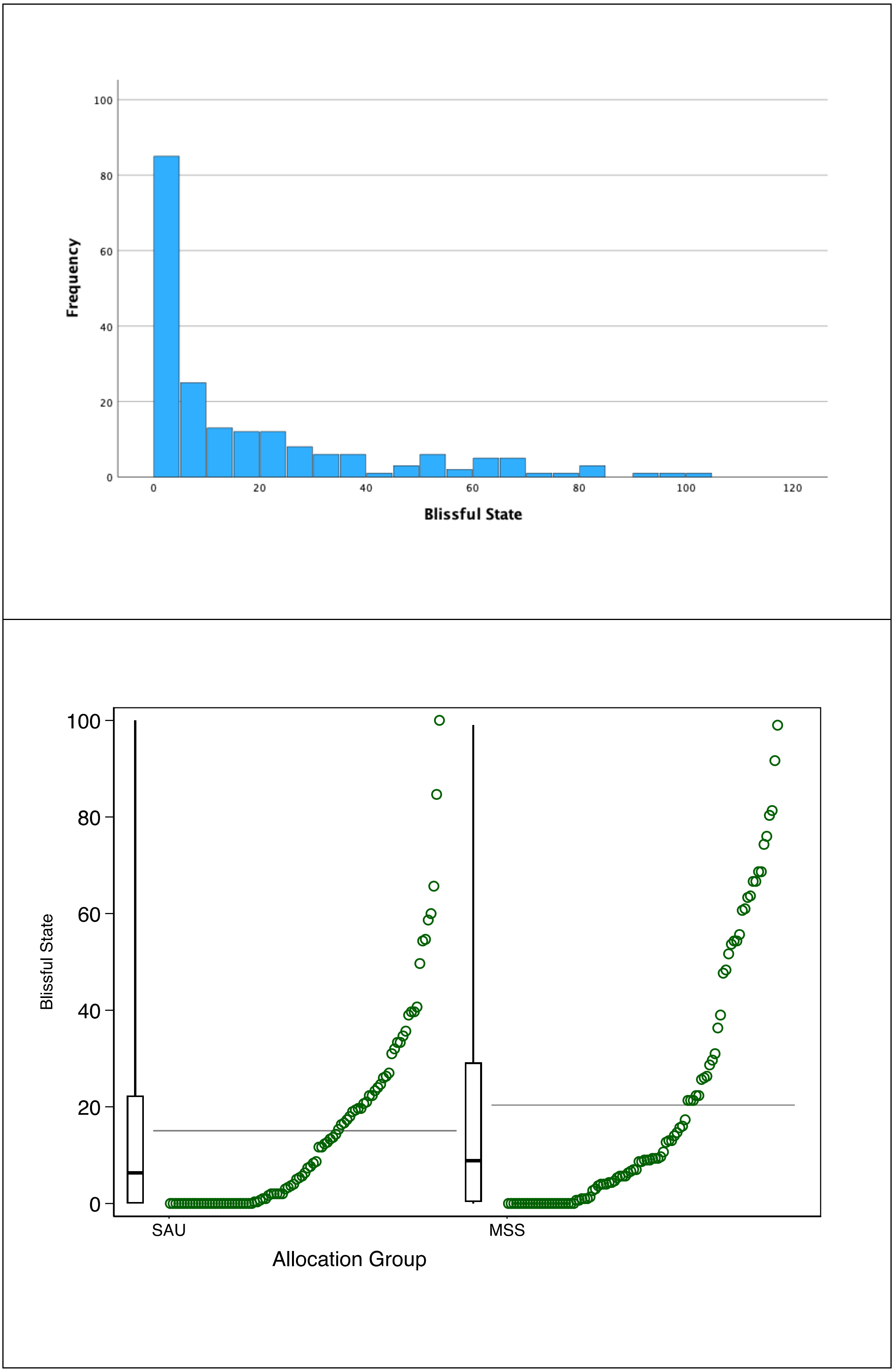

Supplemental Figure S4: Insightfulness

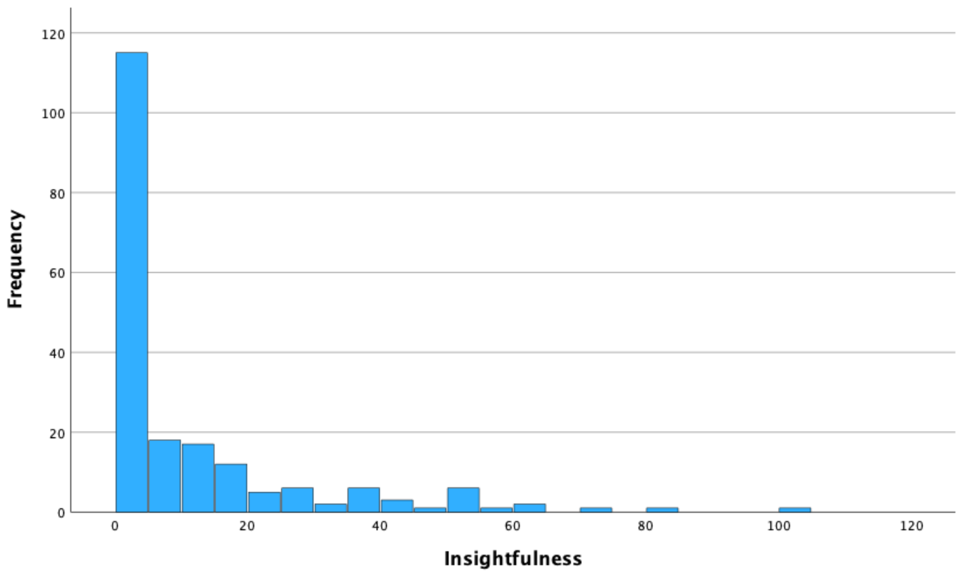

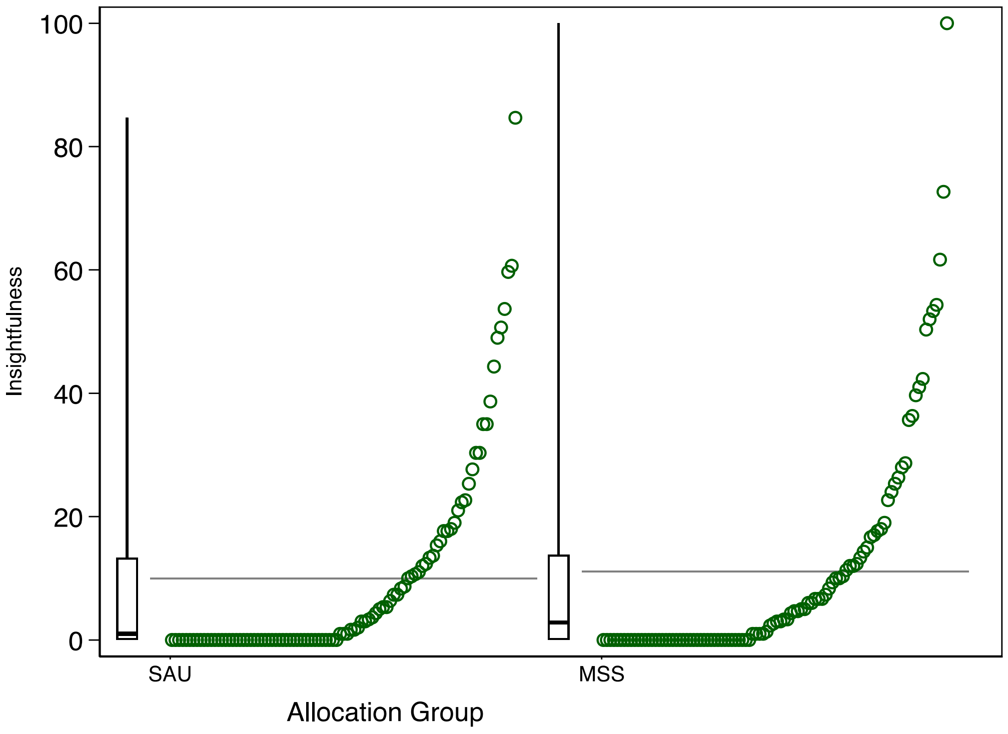

Supplemental Figure S5: Disembodiment

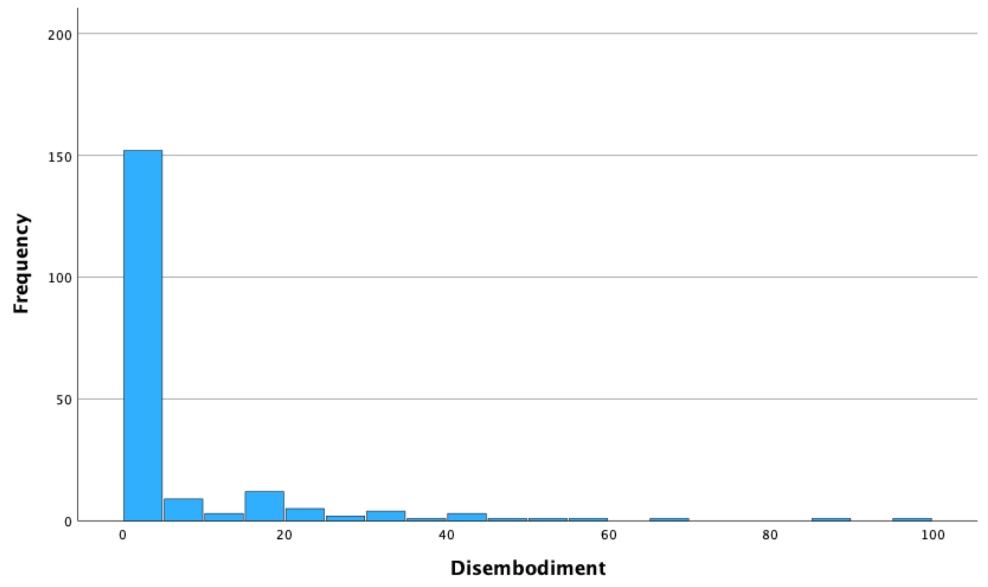

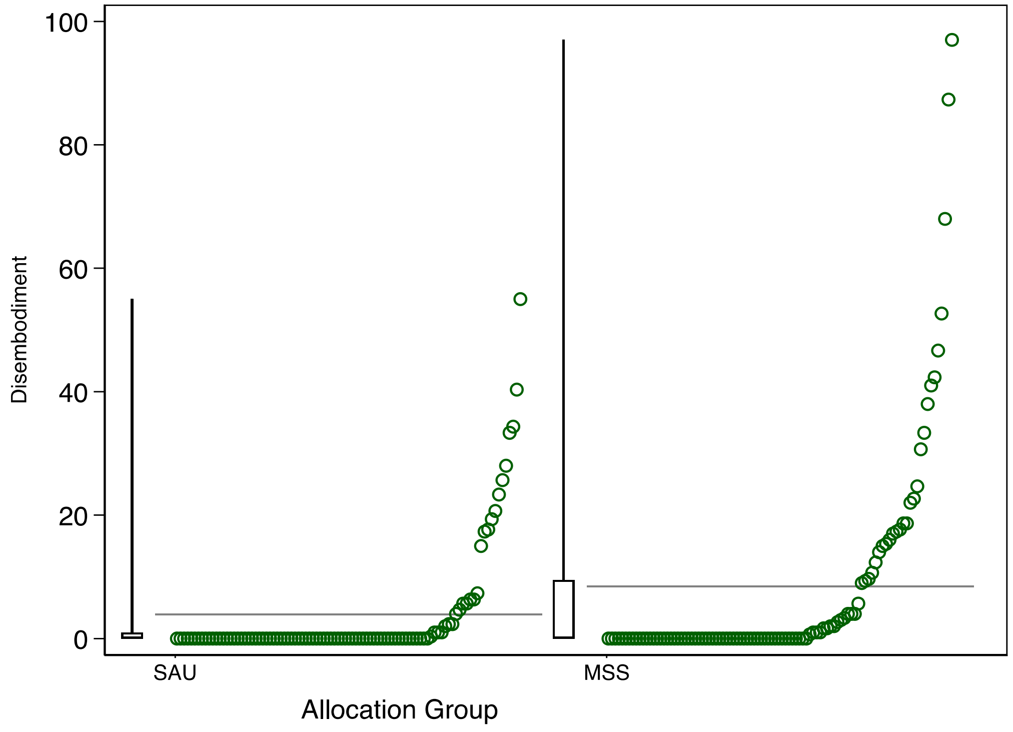

Supplemental Figure S6: Impaired Control and Cognition

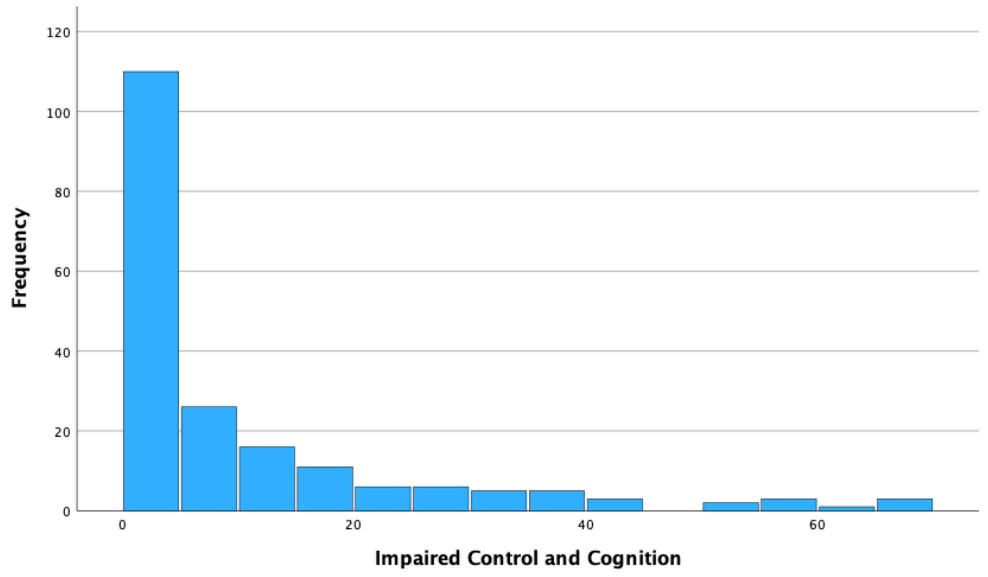

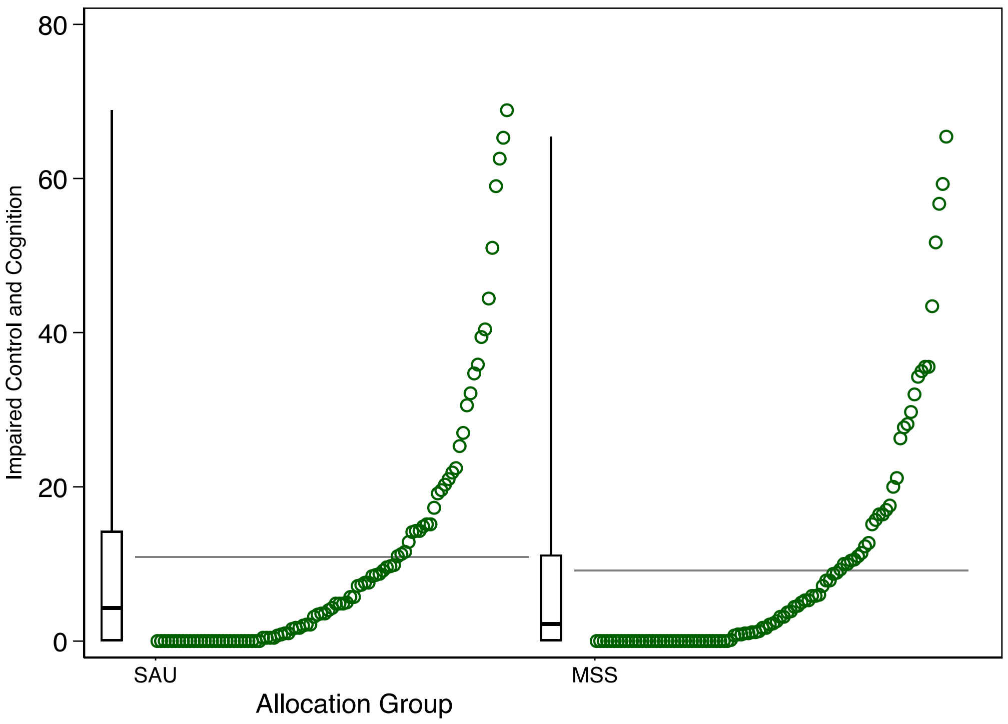

Supplemental Figure S7: Anxiety

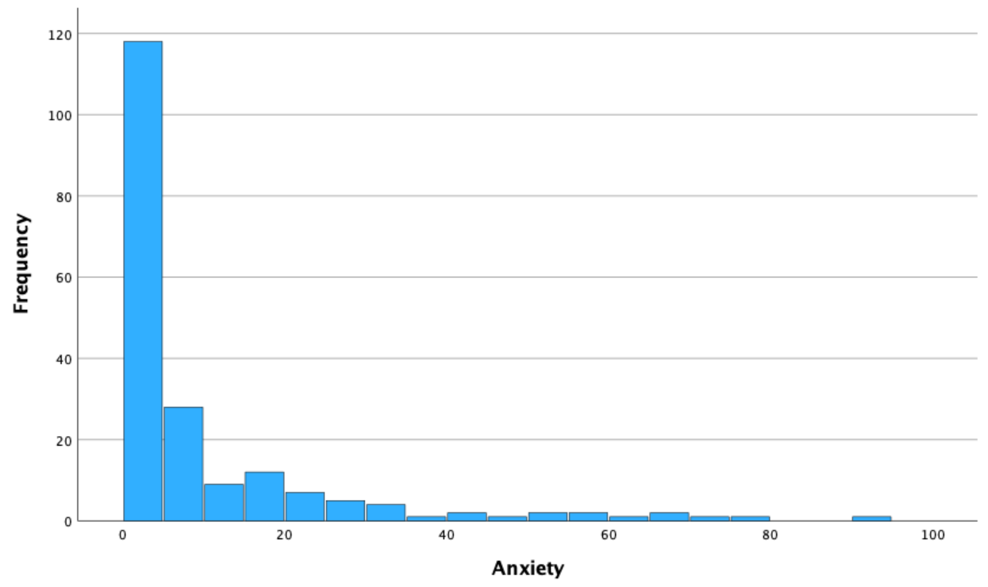

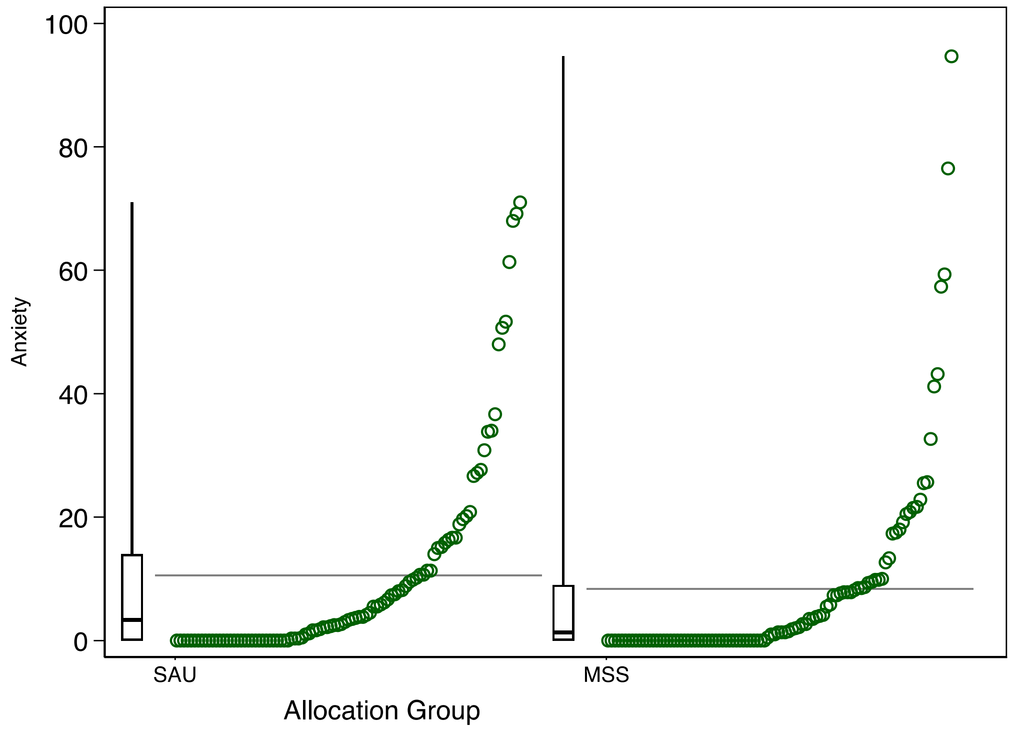

Supplemental Figure S8: Complex Imagery

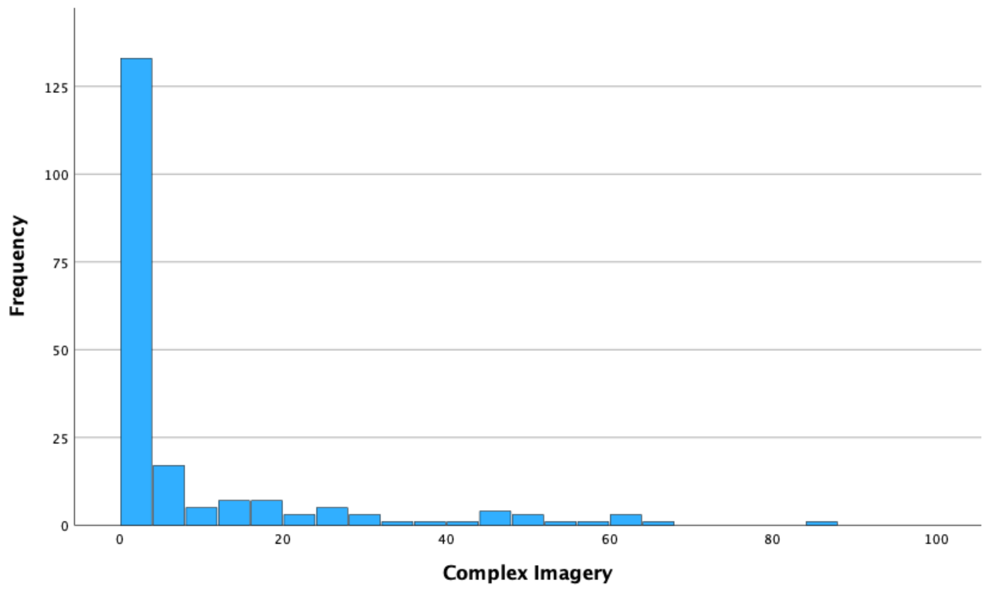

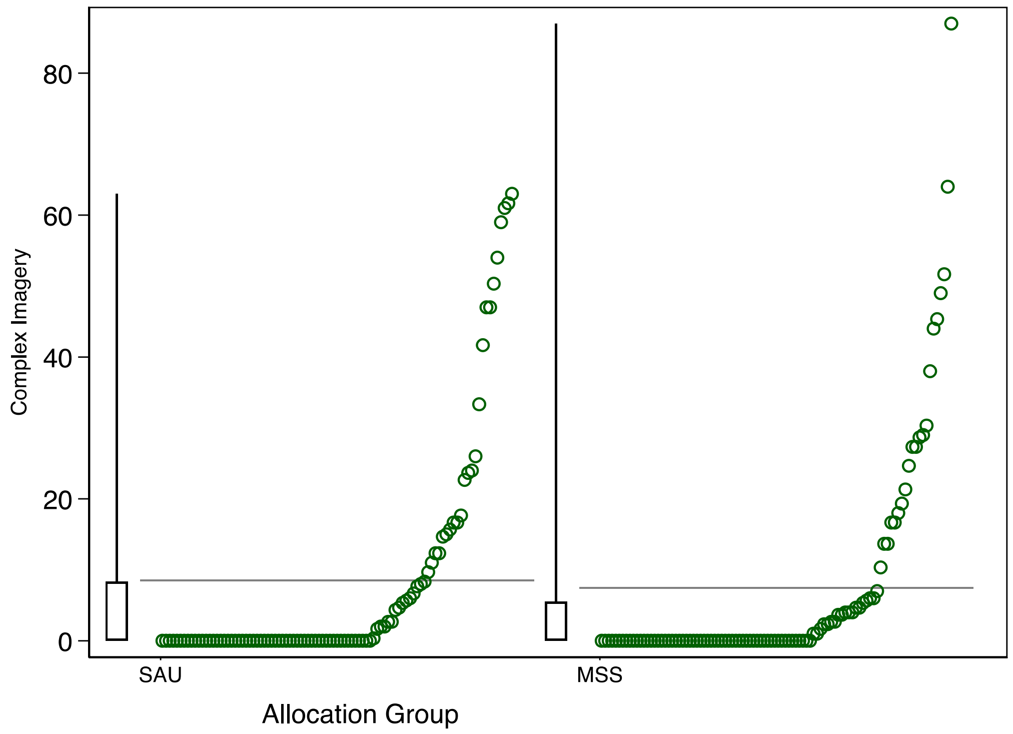

Supplemental Figure S9: Elementary Imagery

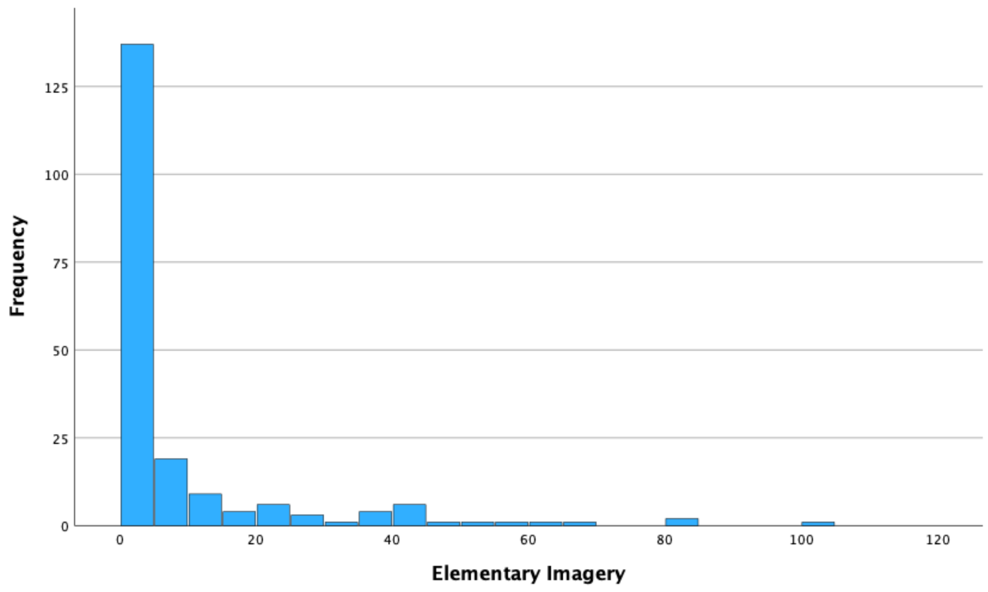

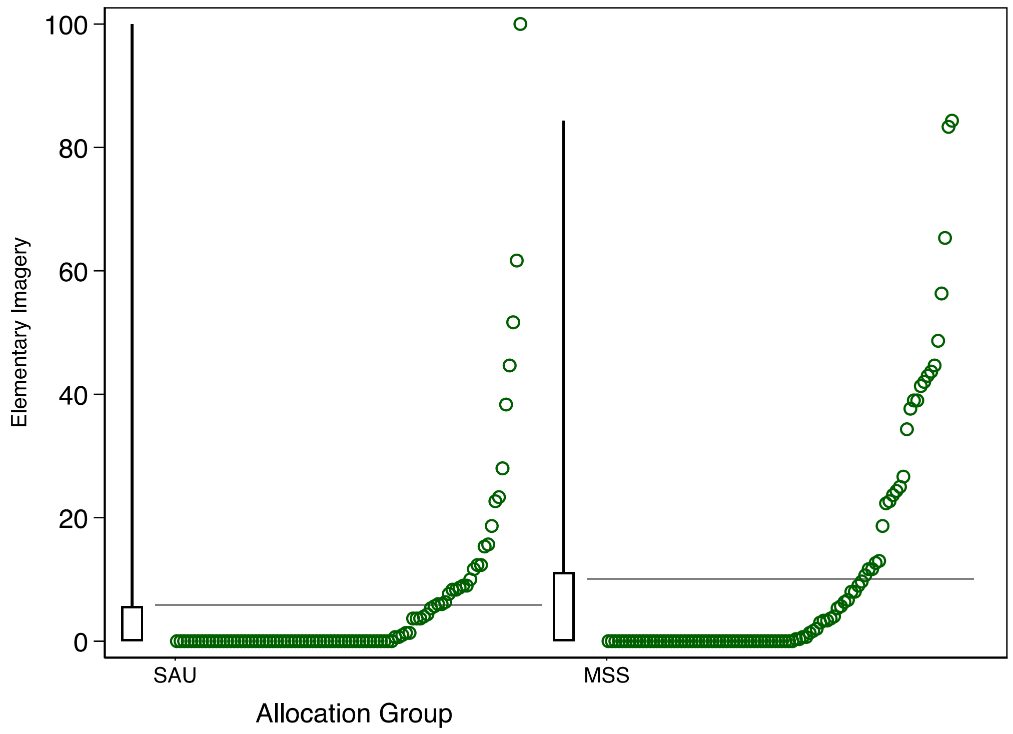

Supplemental Figure S10: Audio-Visual Synesthesiae

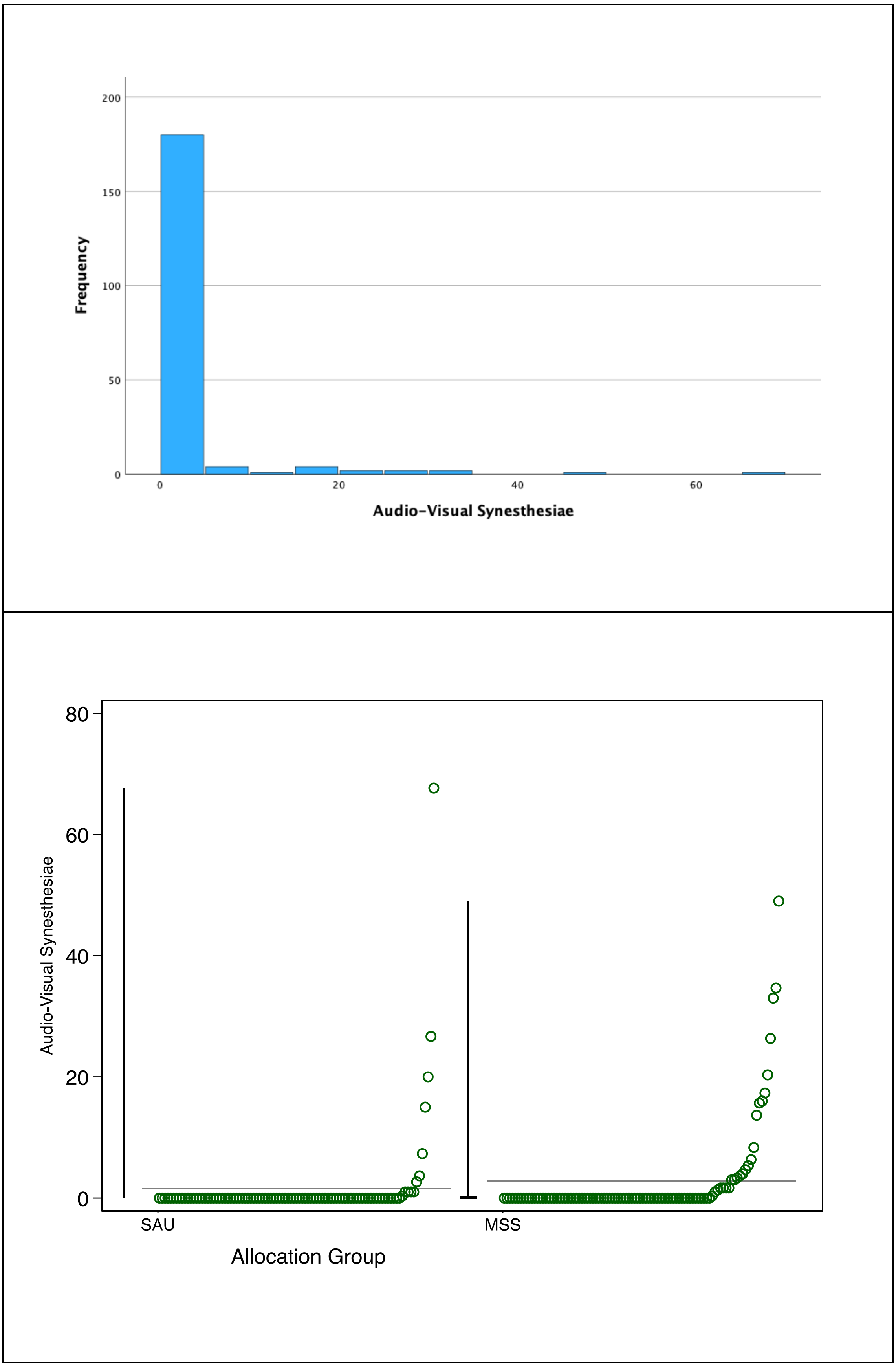

Supplemental Figure S11: Changed Meaning of Percepts

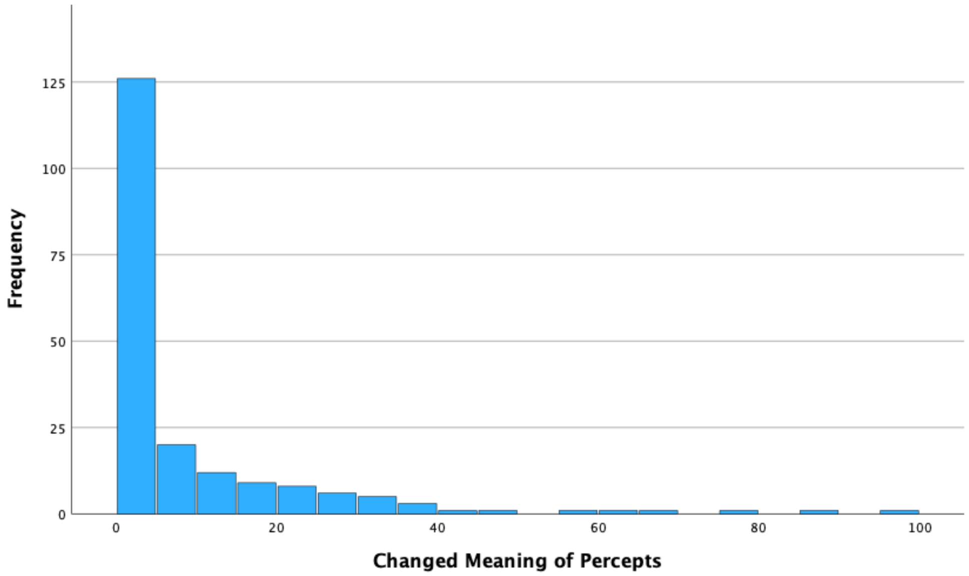

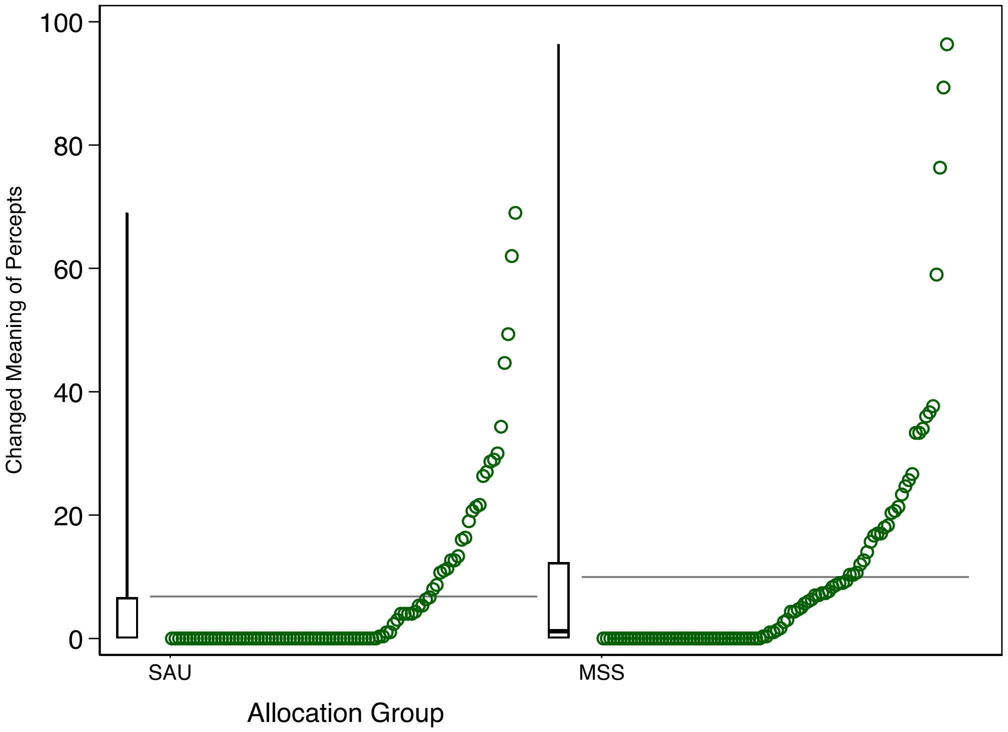

Supplemental Figure S12: relationships between the observed and predicted values for Experience of Unity (allocation group model)

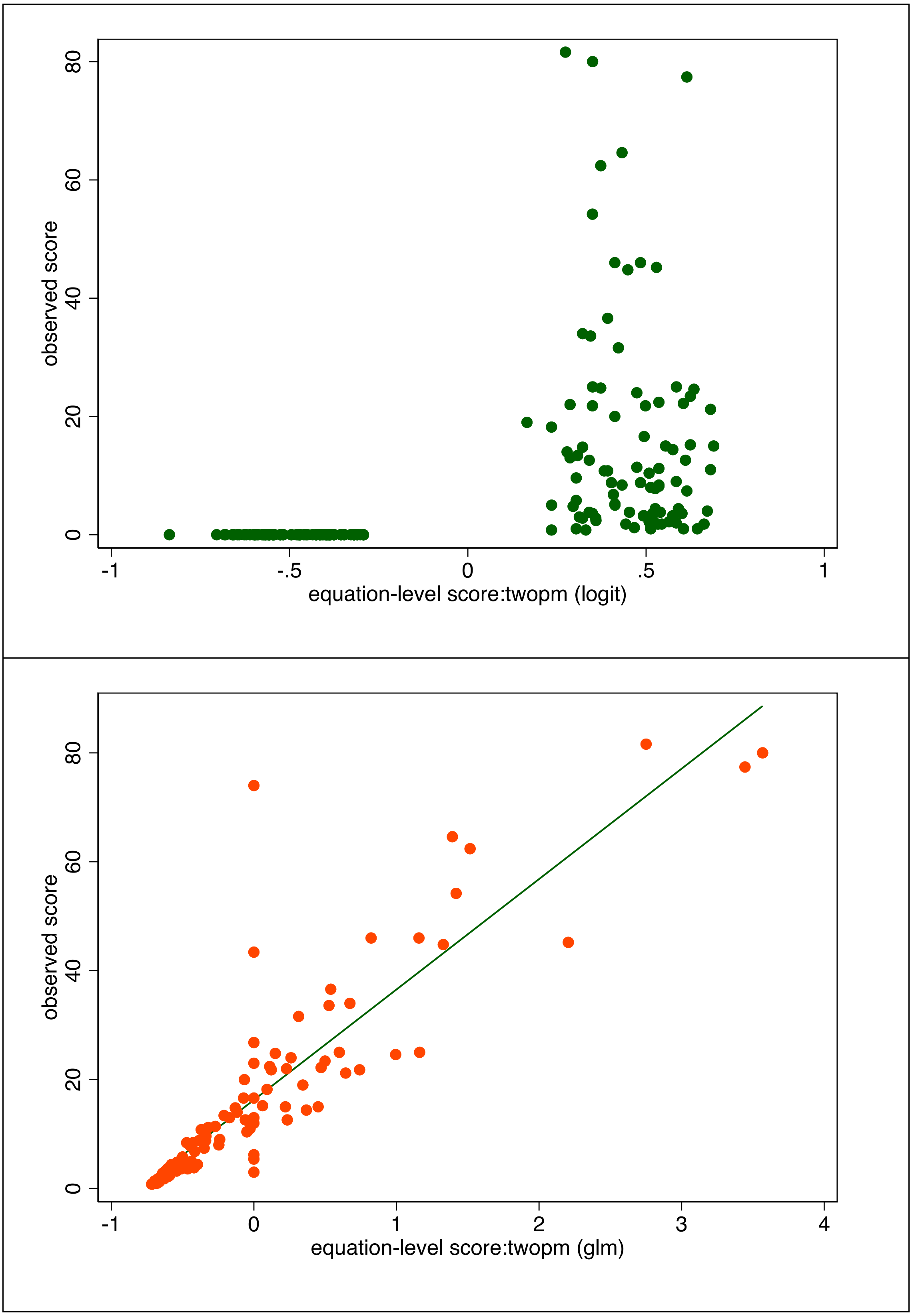

Supplemental Figure S13: relationships between the observed and predicted values for Spiritual Experience (allocation group model)

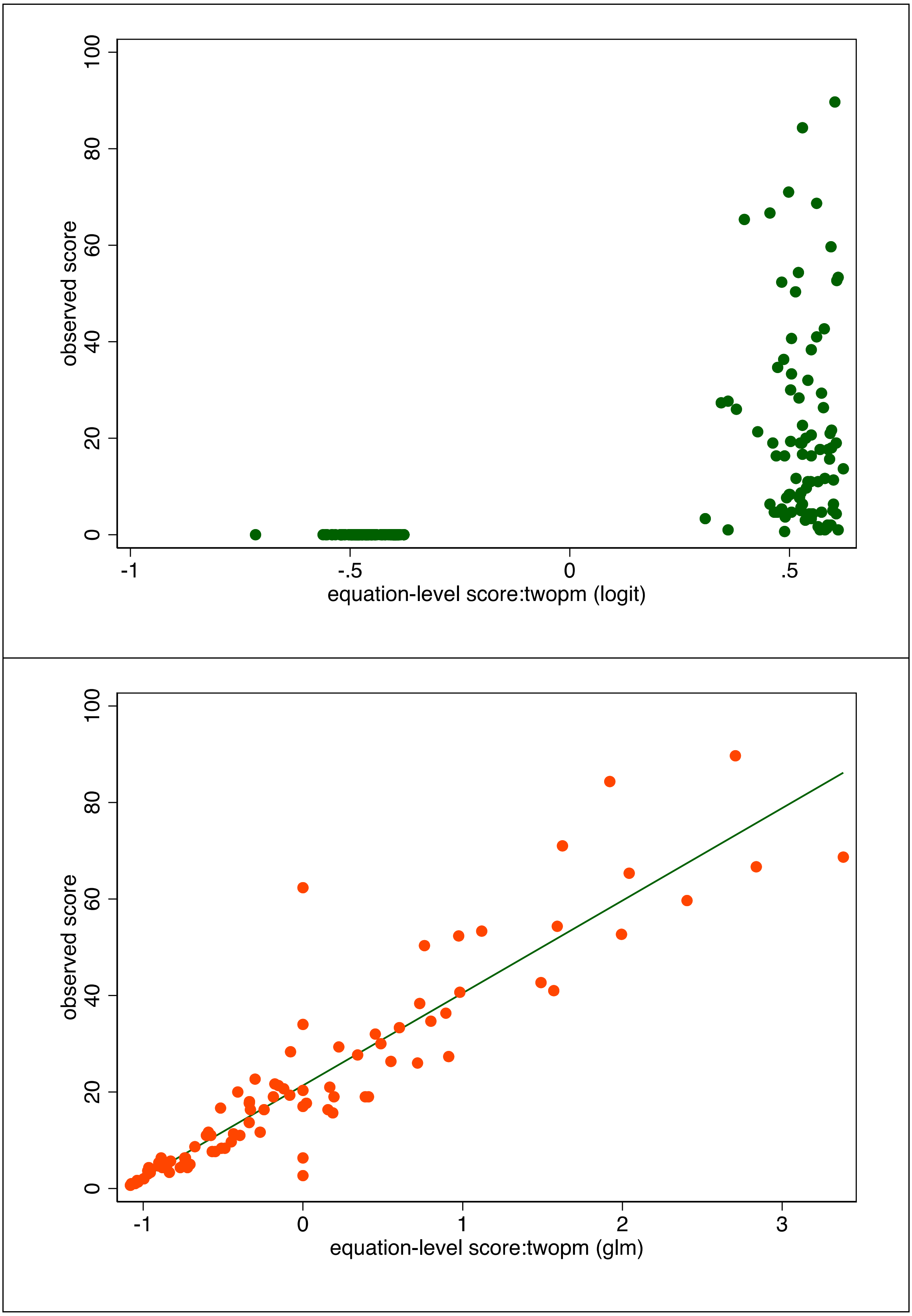

Supplemental Figure S14: relationships between the observed and predicted values for Blissful State (allocation group model)

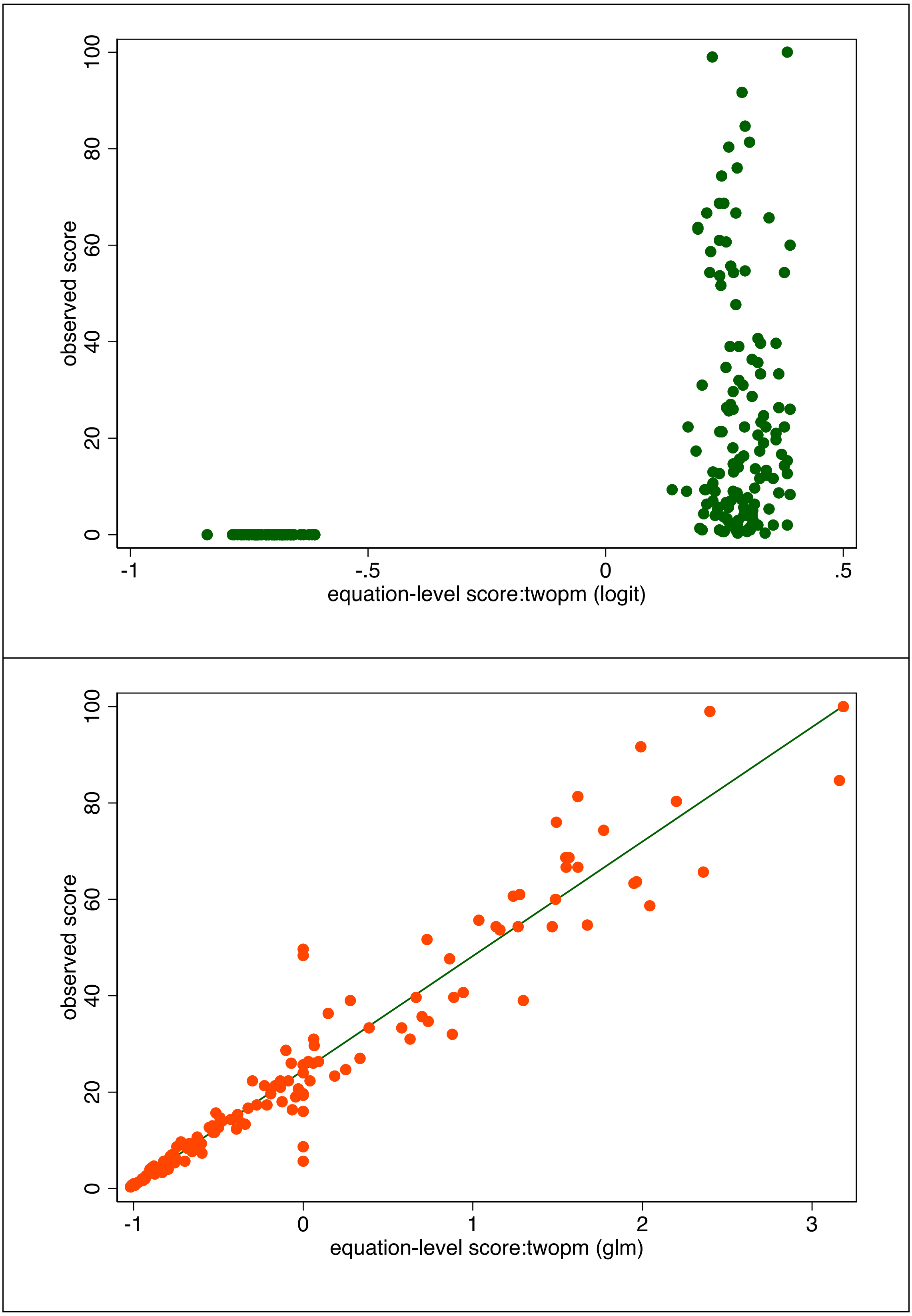

Supplemental Figure S15: relationships between the observed and predicted values for Insightfulness (allocation group model)

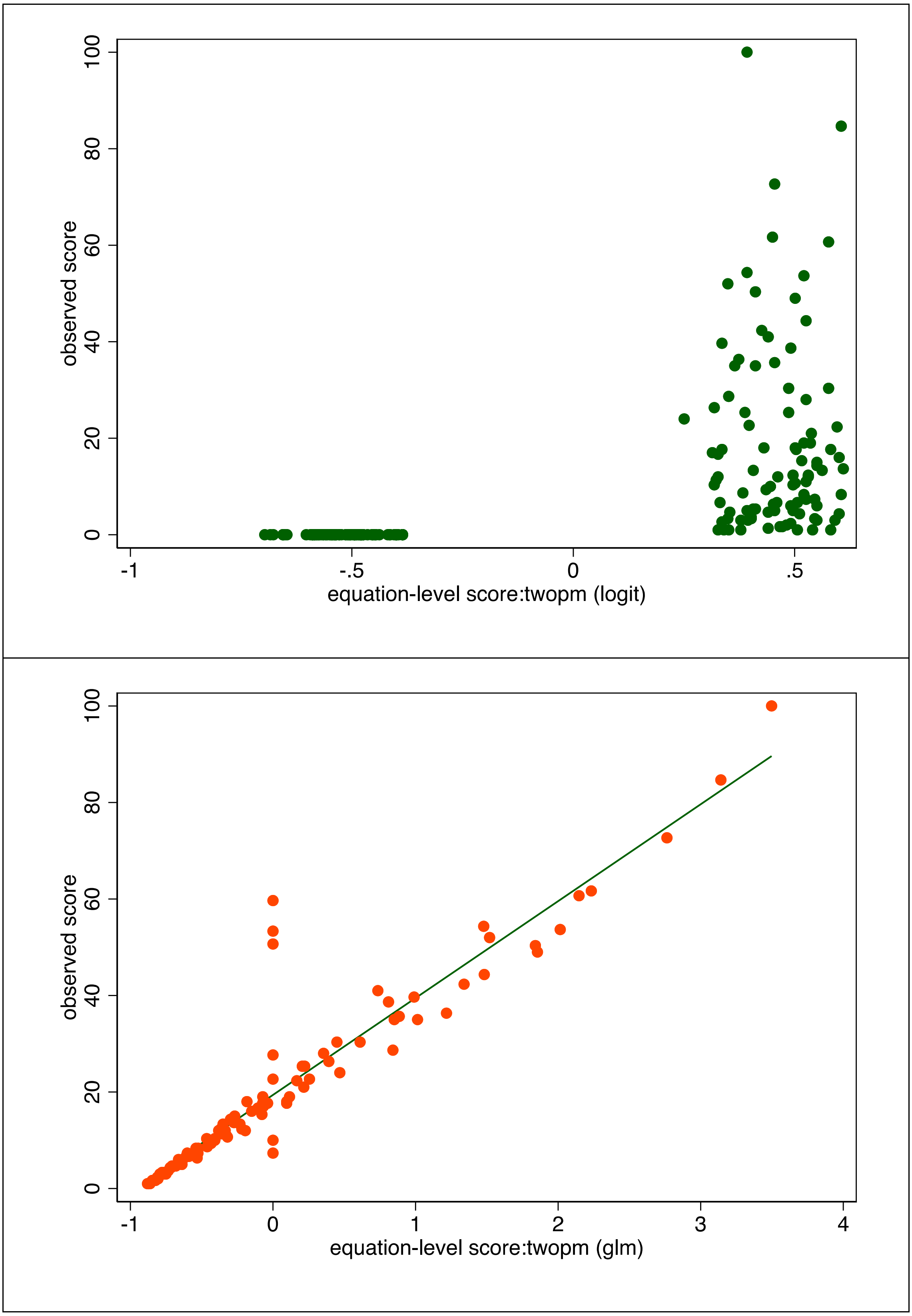

Supplemental Figure S16: relationships between the observed and predicted values for Disembodiment (allocation group model)

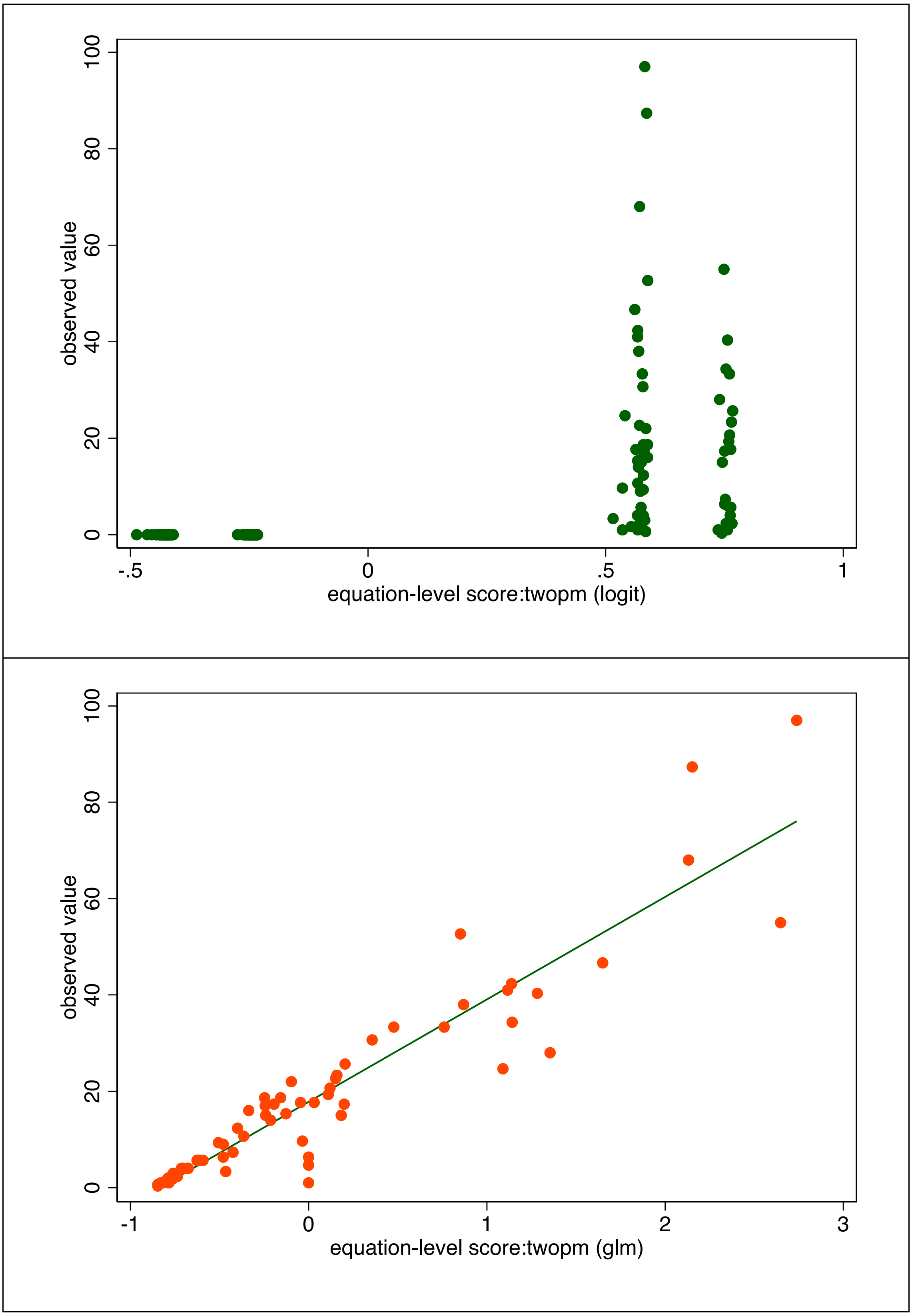

Supplemental Figure S17: relationships between the observed and predicted values for Impaired control & cognition (allocation group model)

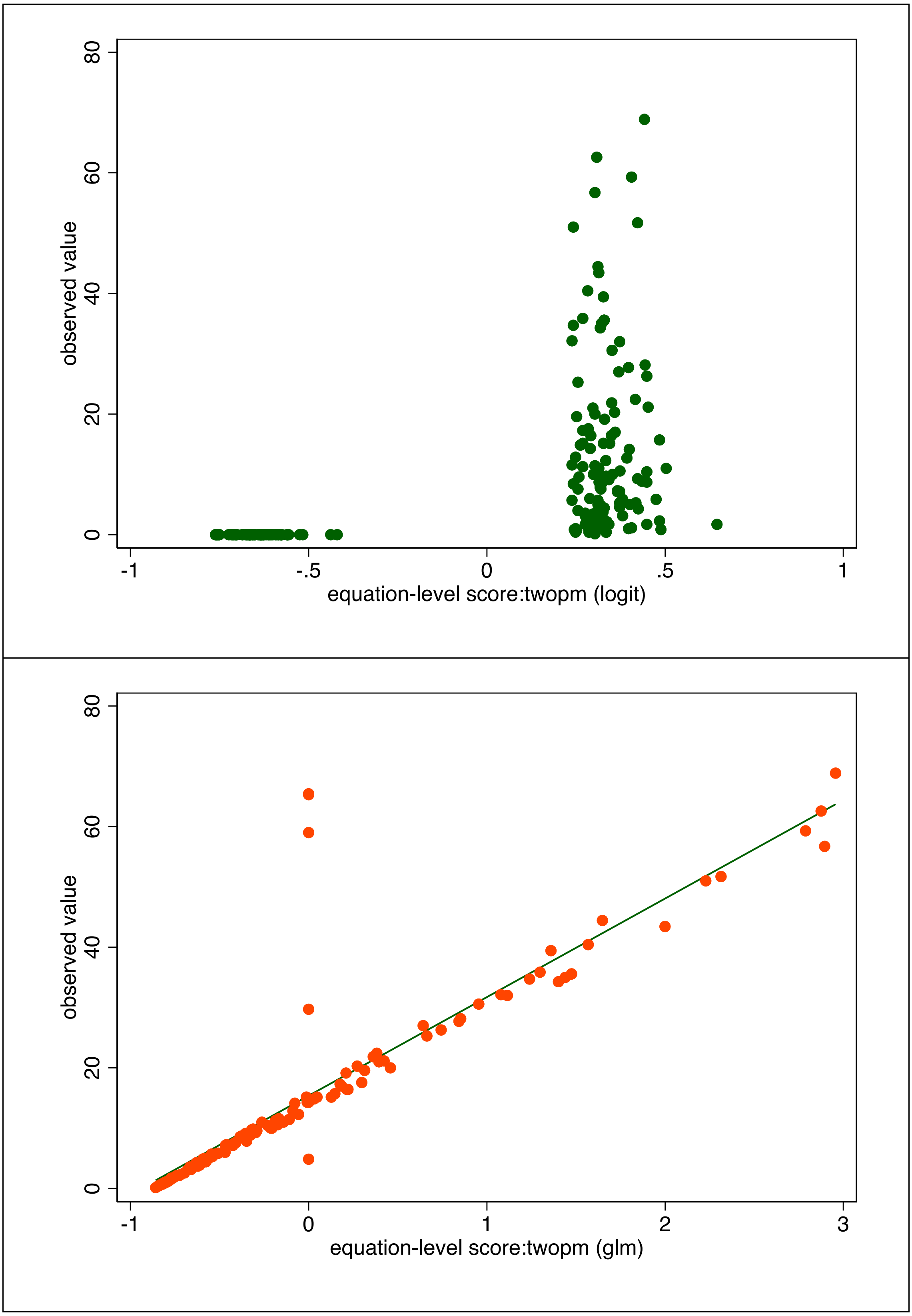

Supplemental Figure S18: relationships between the observed and predicted values for Anxiety (allocation group model)

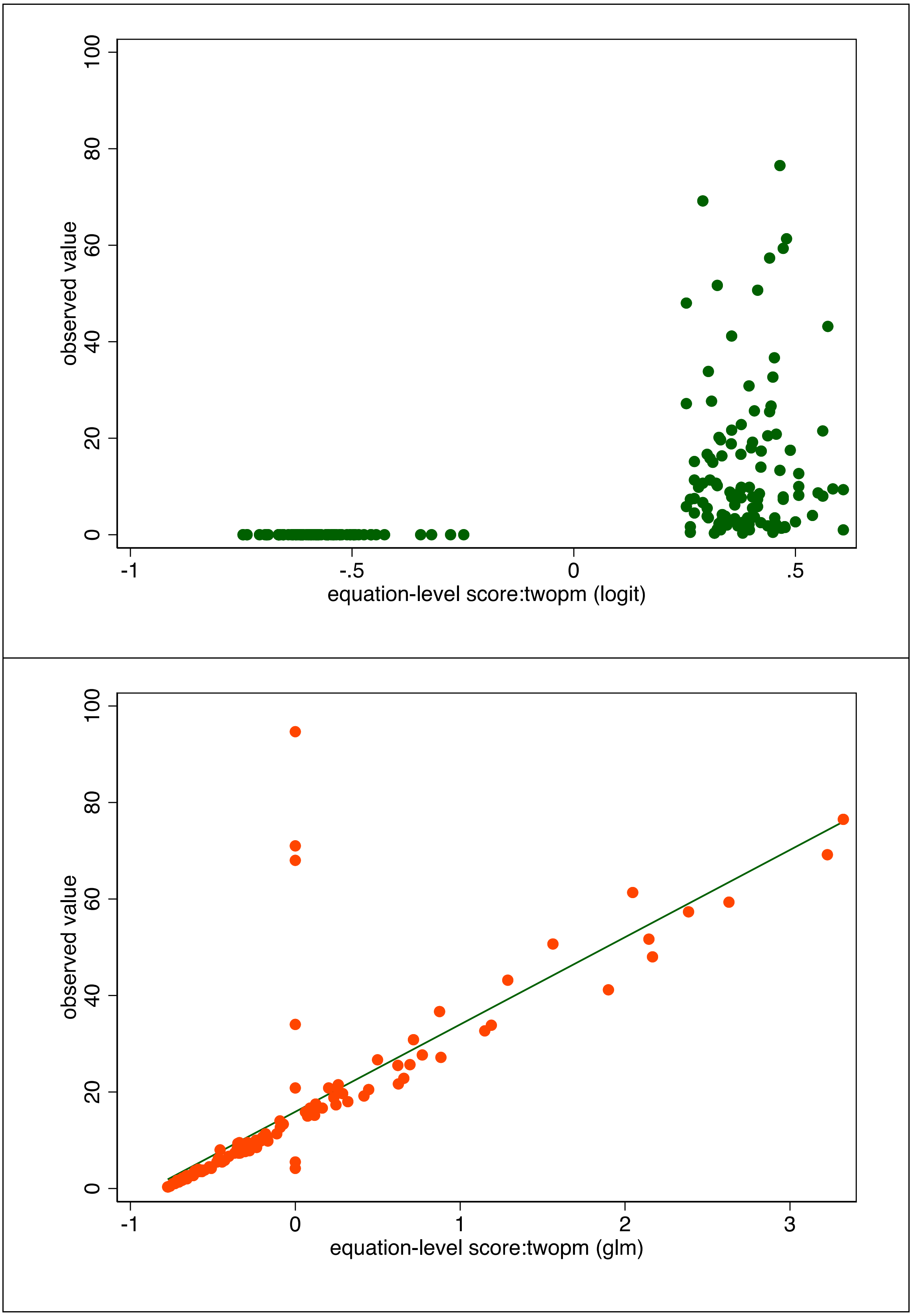

Supplemental Figure S19: relationships between the observed and predicted values for Complex Imagery (allocation group model)

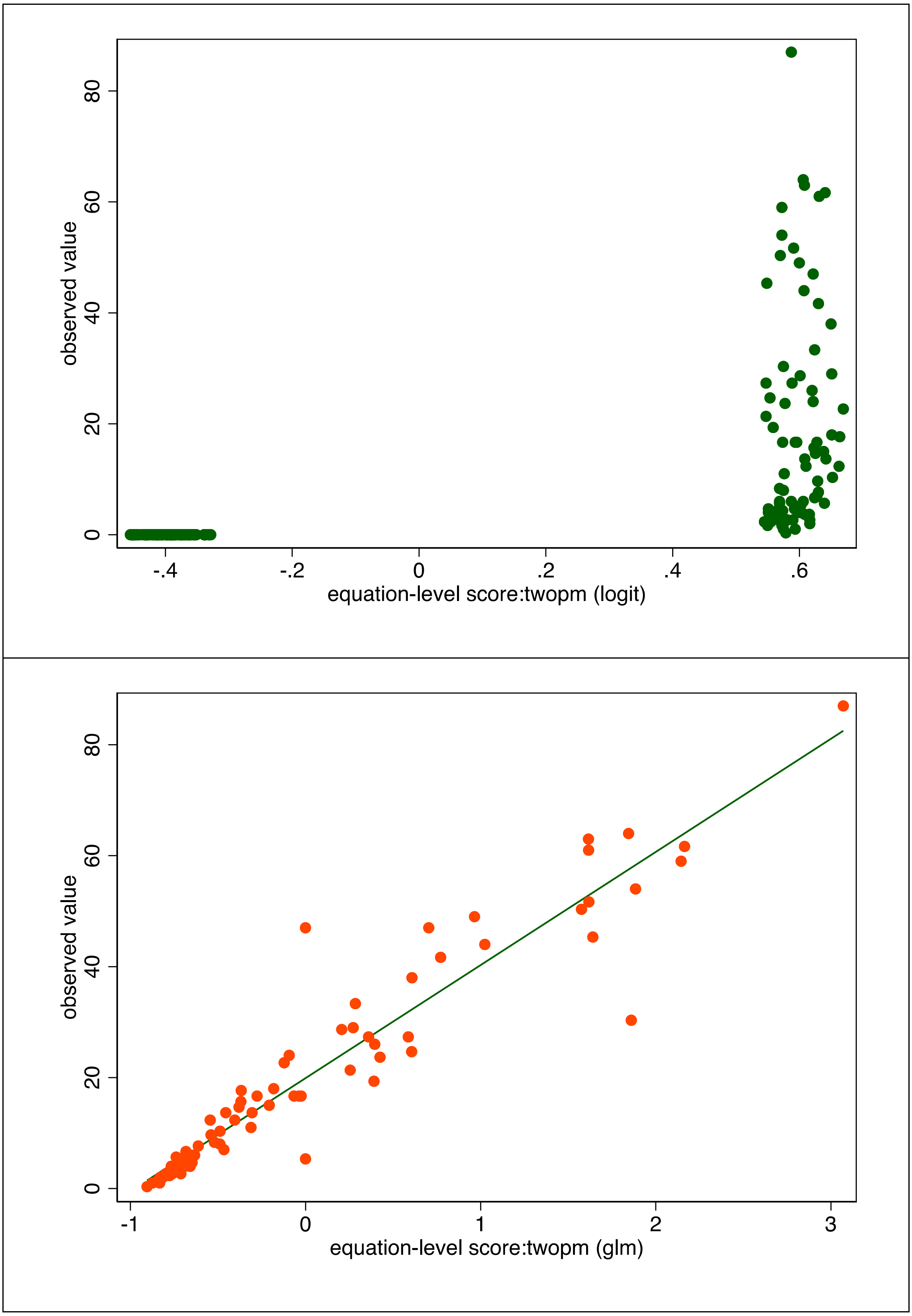

Supplemental Figure S20: relationships between the observed and predicted values for Elementary Imagery (allocation group model)

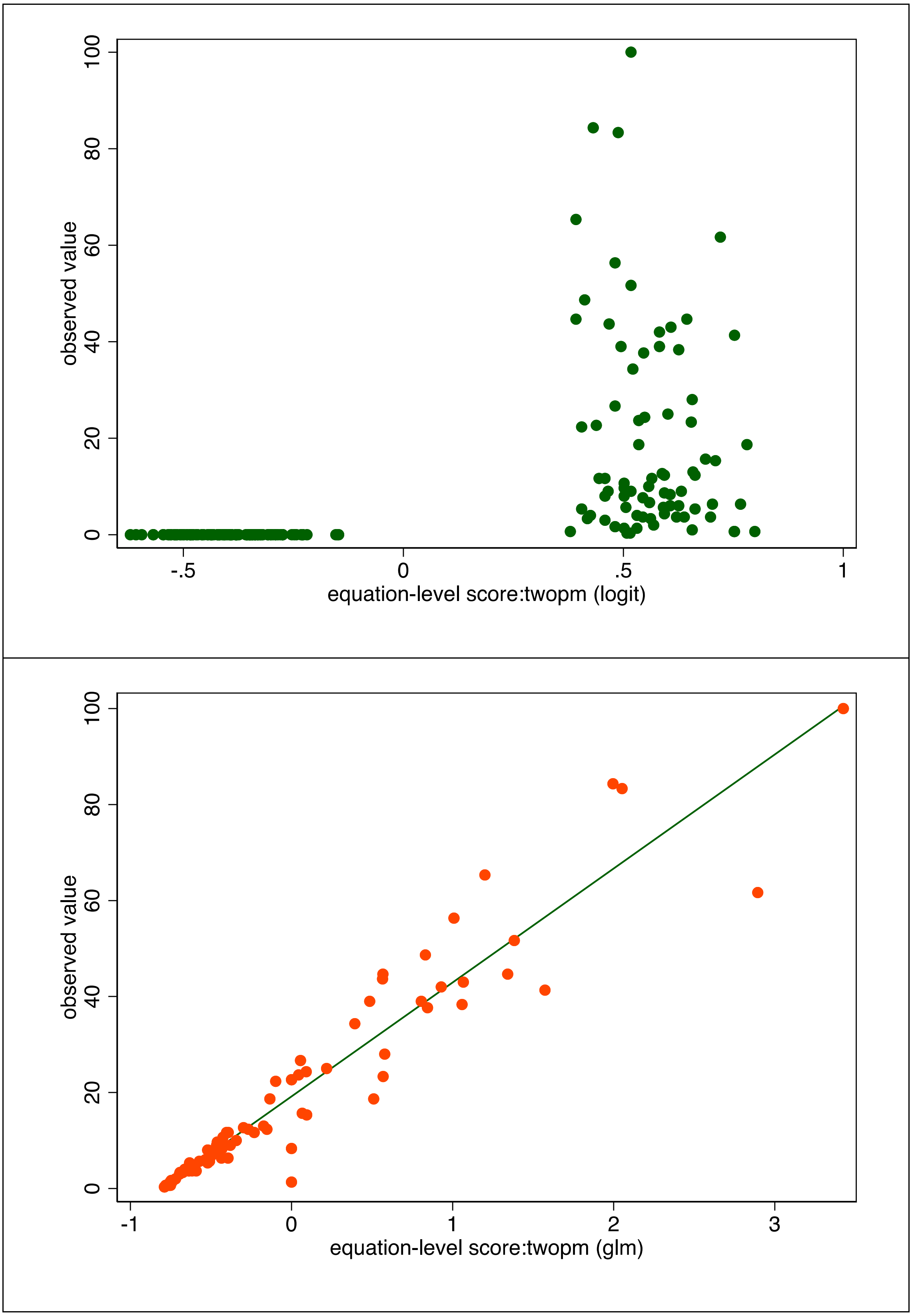

Supplemental Figure S21: relationships between the observed and predicted values for Audiovisual Synesthesiae (allocation group model)

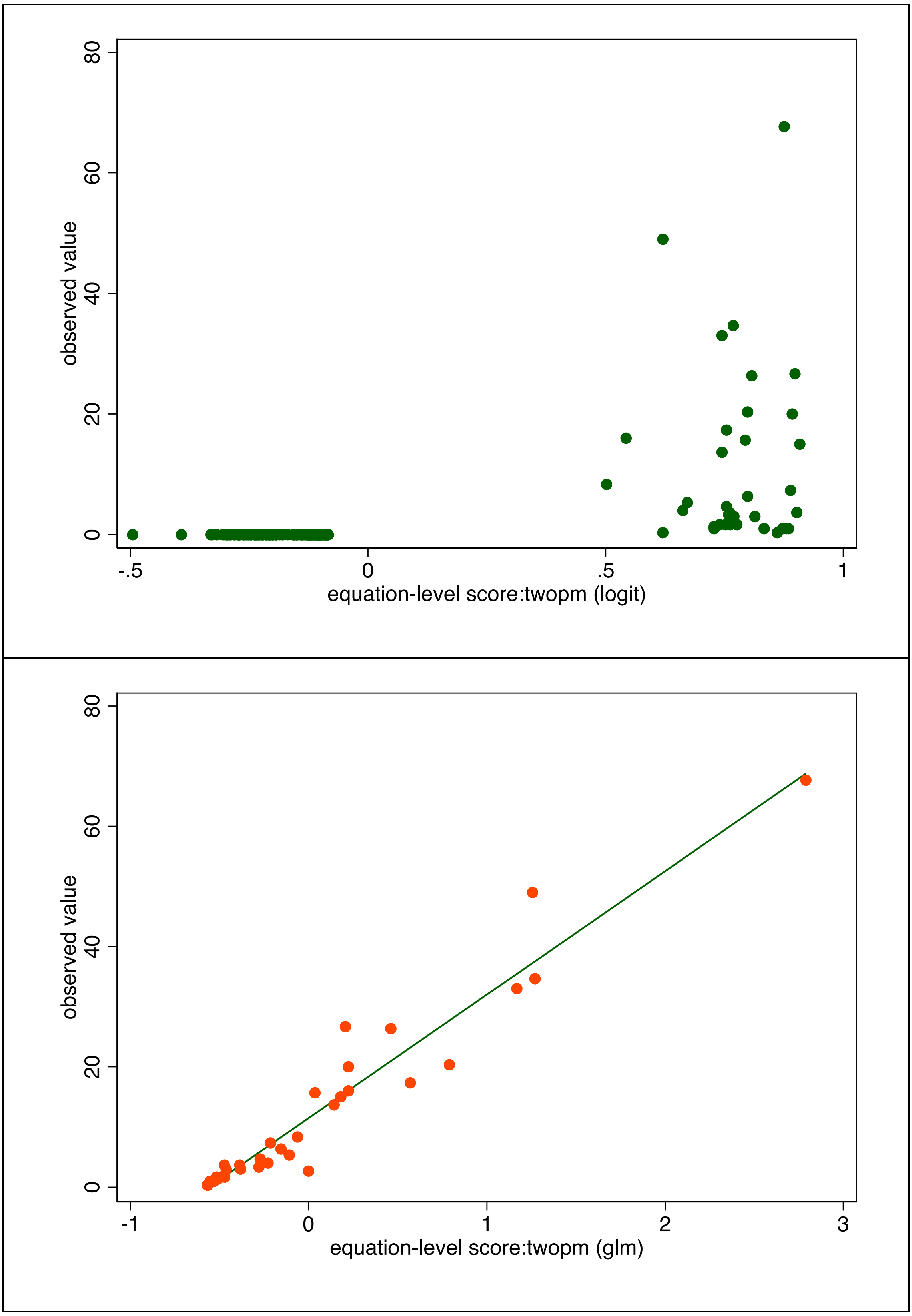

Supplemental Figure S22: relationships between the observed and predicted values for Changed meaning of percepts (allocation group model)

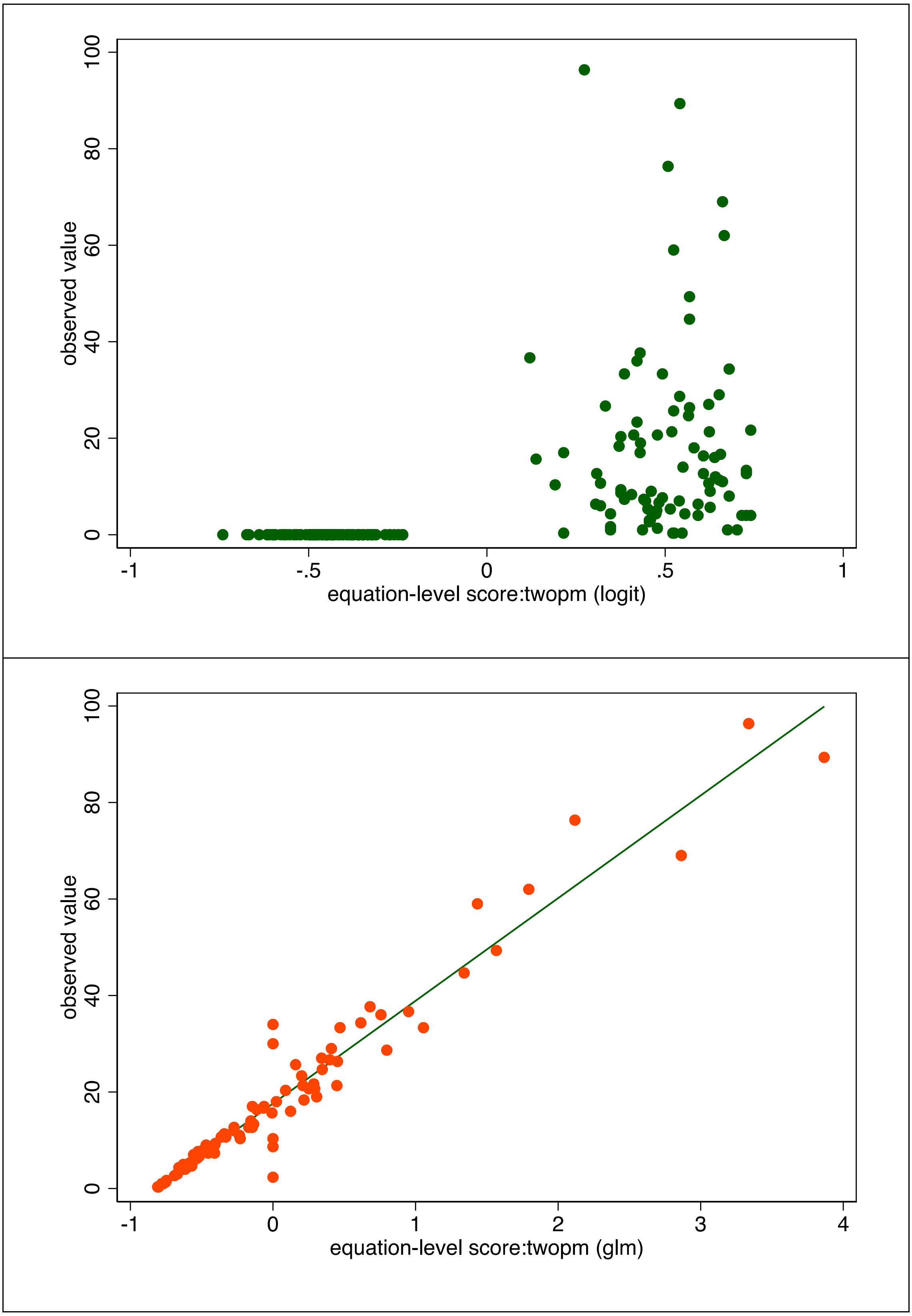

Supplemental Figure S23. Mindfulness meditation practice (hours) during the whole study by group (intervention group above, control group below).

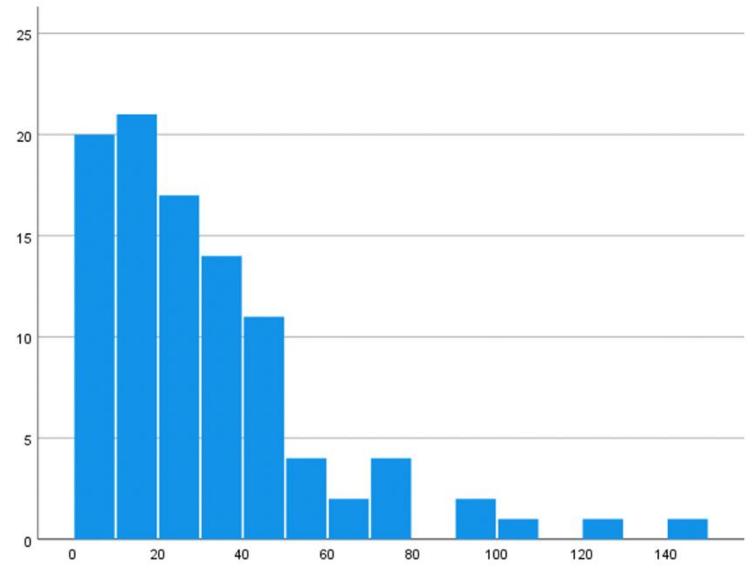

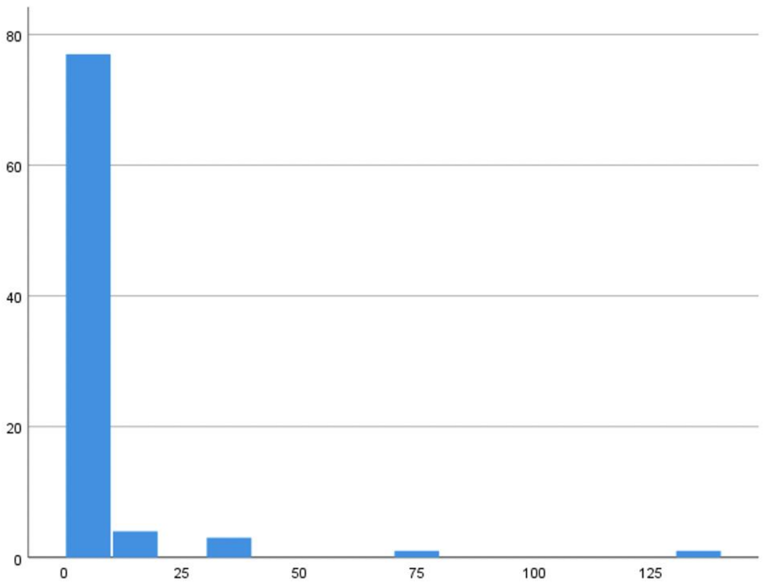

Supplemental Figure S14. Informal mindfulness meditation practice (intervention arm, n = 99).

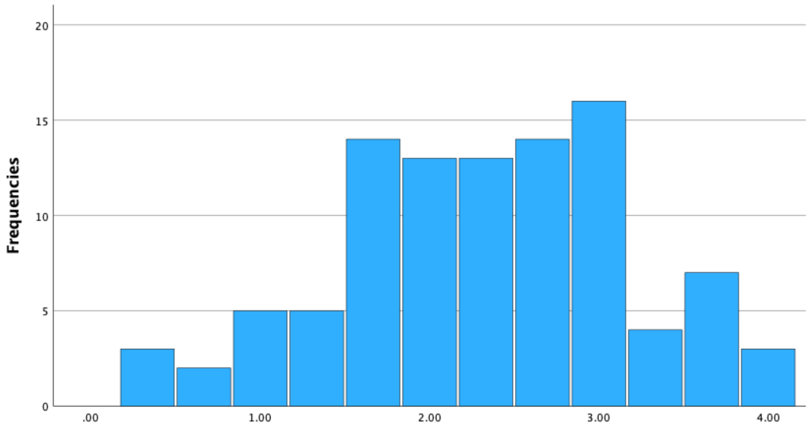

0 = Never, 1 = Rarely, 2 = Sometimes, 3 = Often, 4 = Very often.

Supplemental Figure S25: relationships between the observed and predicted values for Experience of Unity (formal mindfulness practice model)

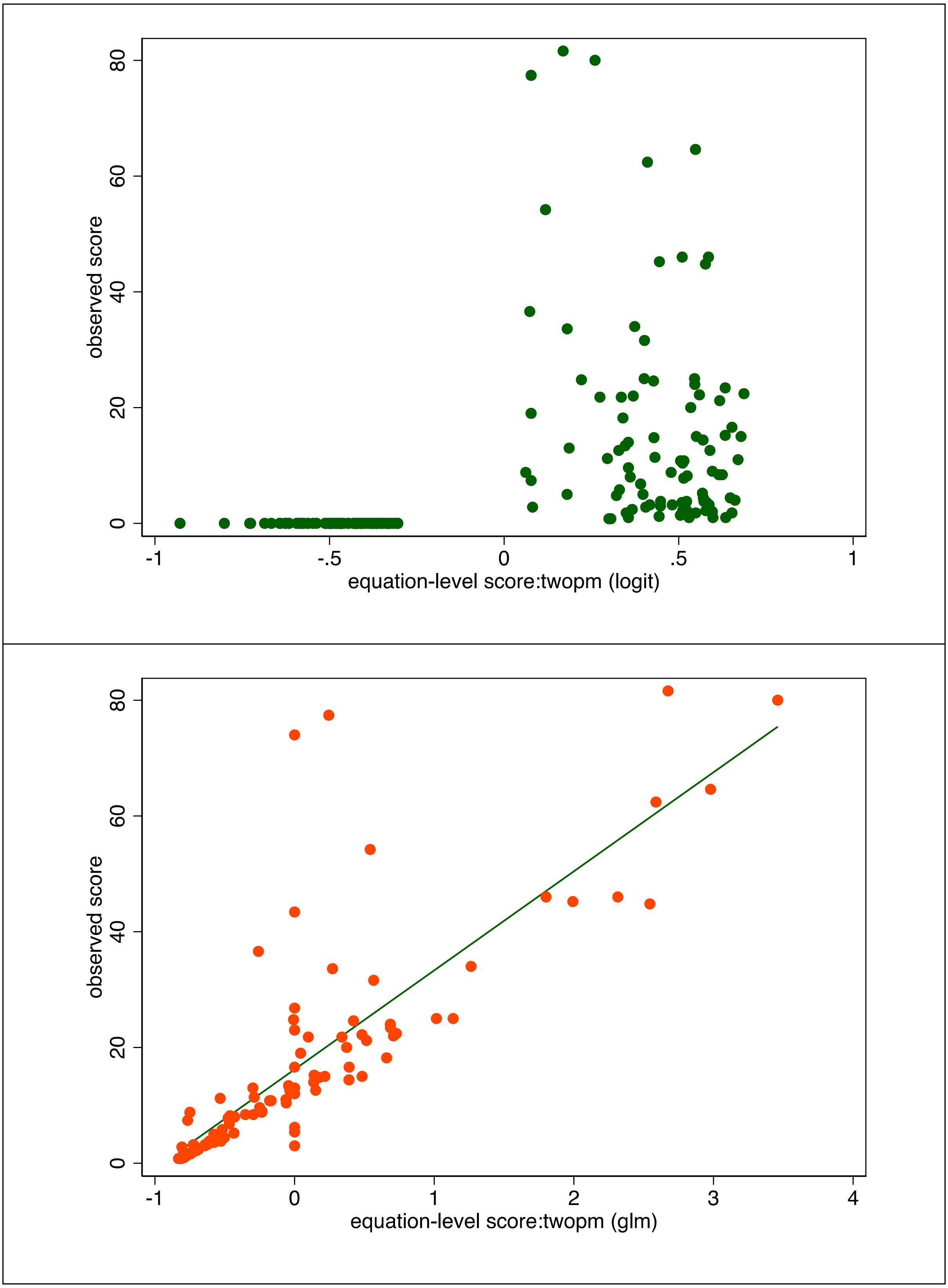

Supplemental Figure S26: relationships between the observed and predicted values for Spiritual Experience (formal mindfulness practice model)

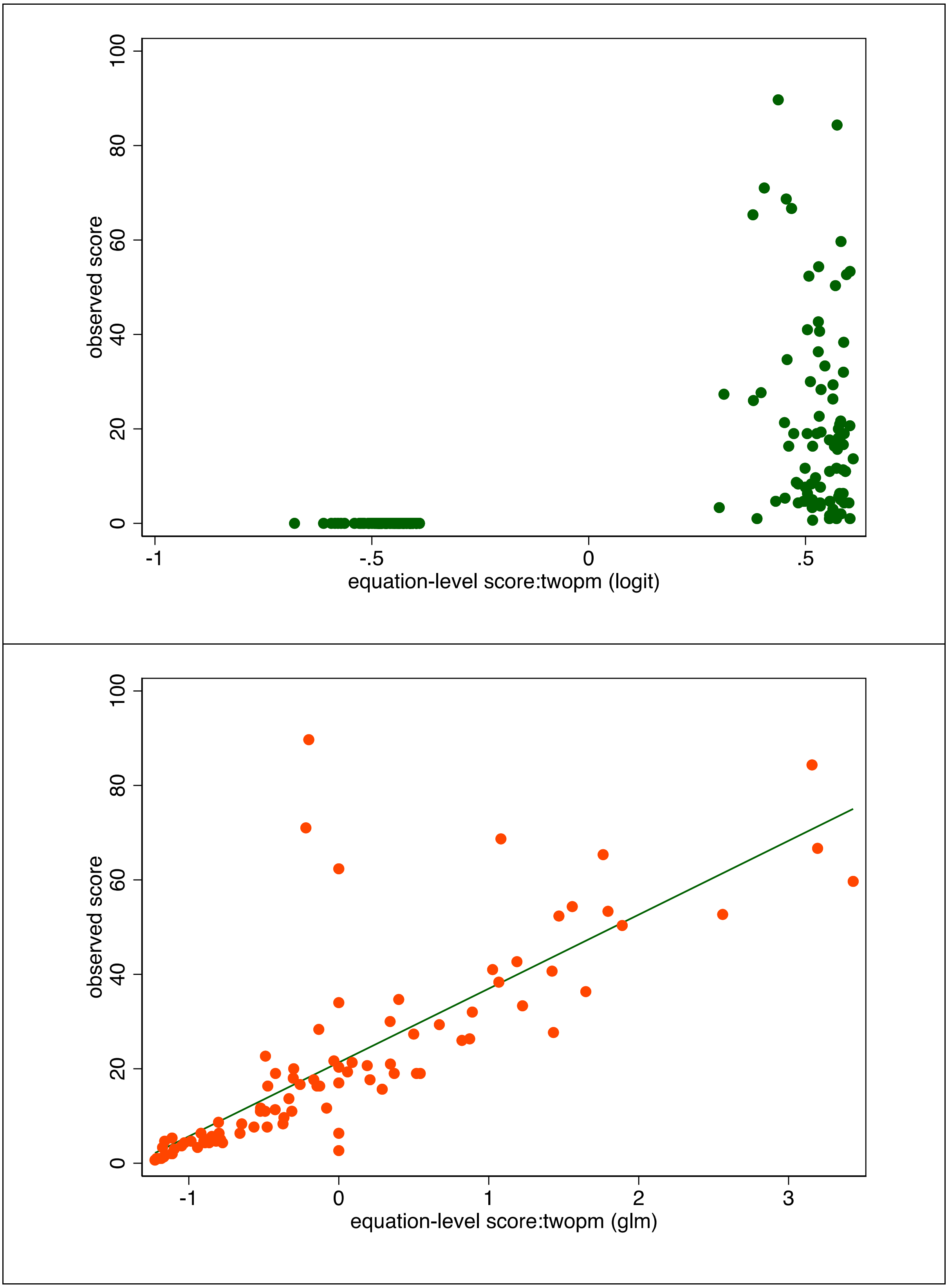

Supplemental Figure S27: relationships between the observed and predicted values for Blissful state (formal mindfulness practice model)

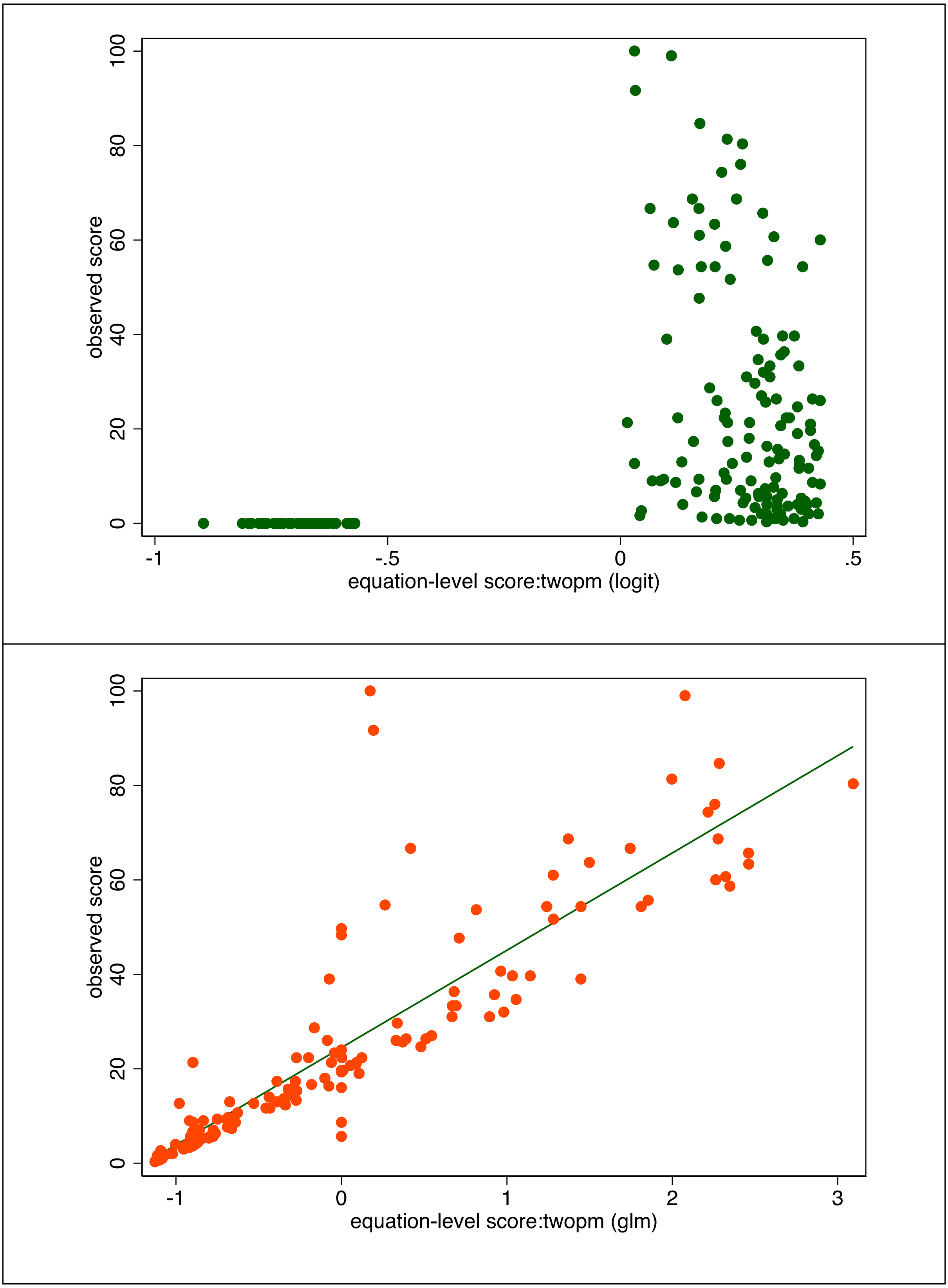

Supplemental Figure S28: relationships between the observed and predicted values for Insightfulness (formal mindfulness practice model)

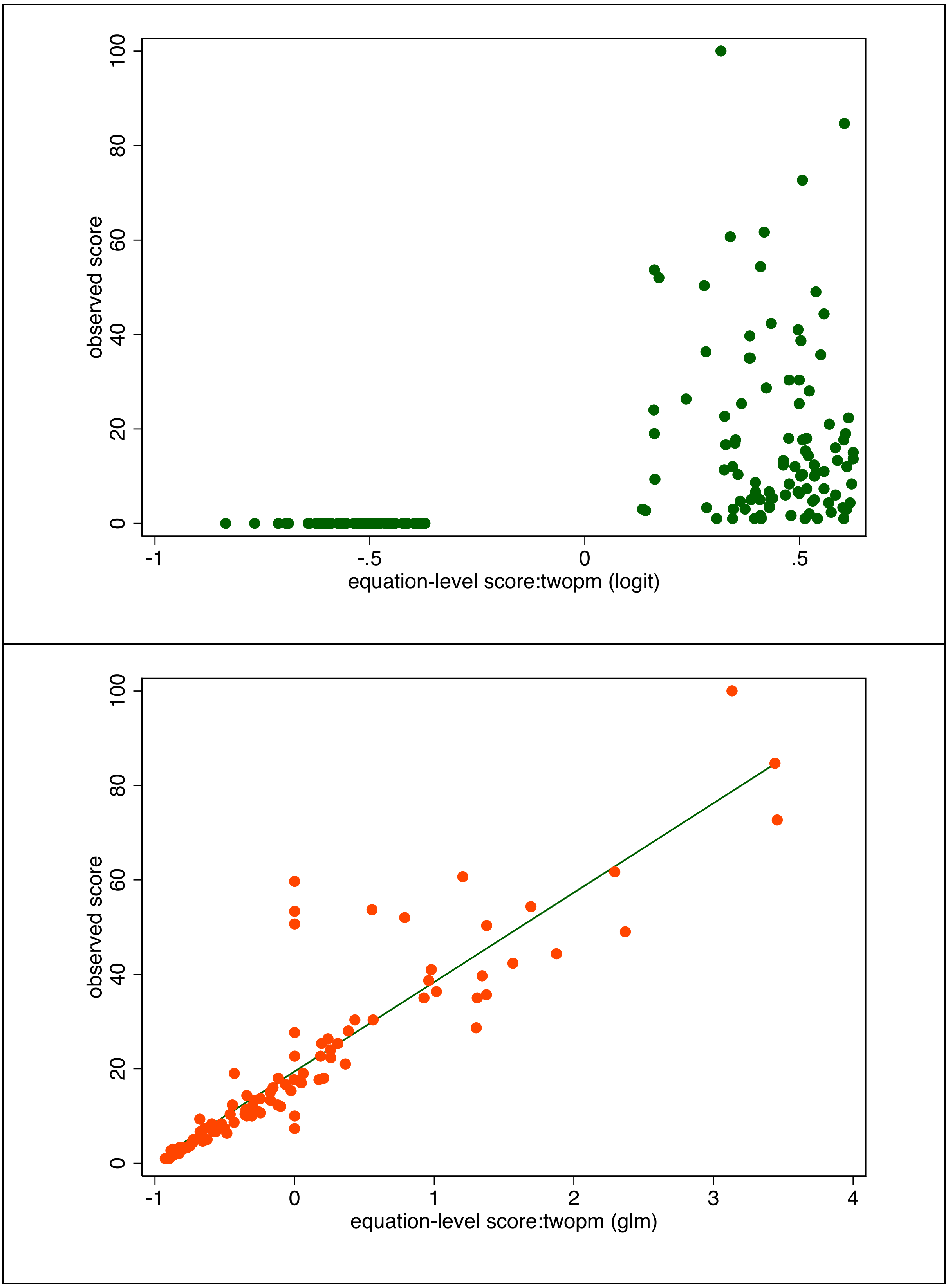

Supplemental Figure S29: relationships between the observed and predicted values for Disembodiment (formal mindfulness practice model)

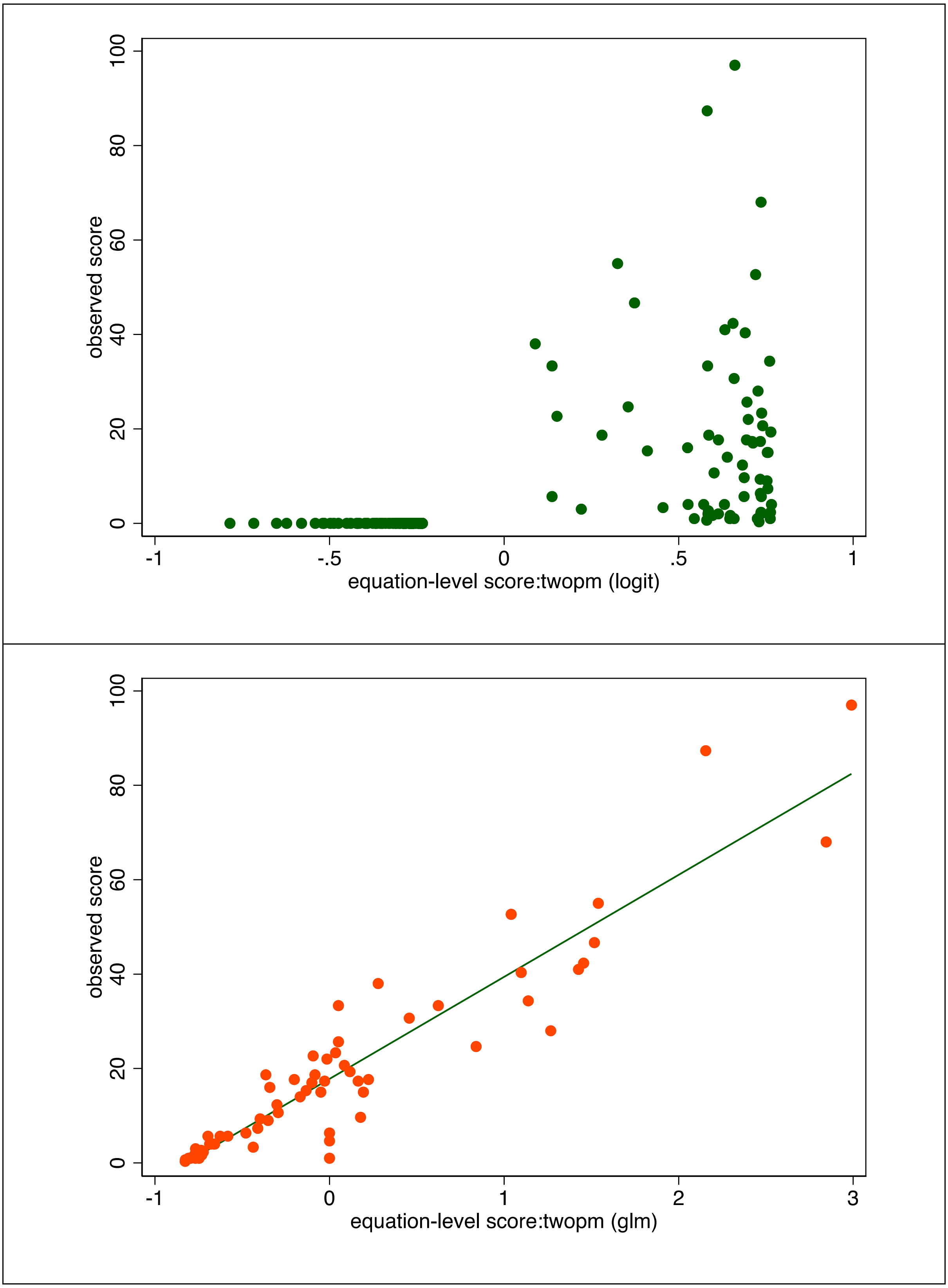

Supplemental Figure S30: relationships between the observed and predicted values for Impaired control & cognition (formal mindfulness practice model)

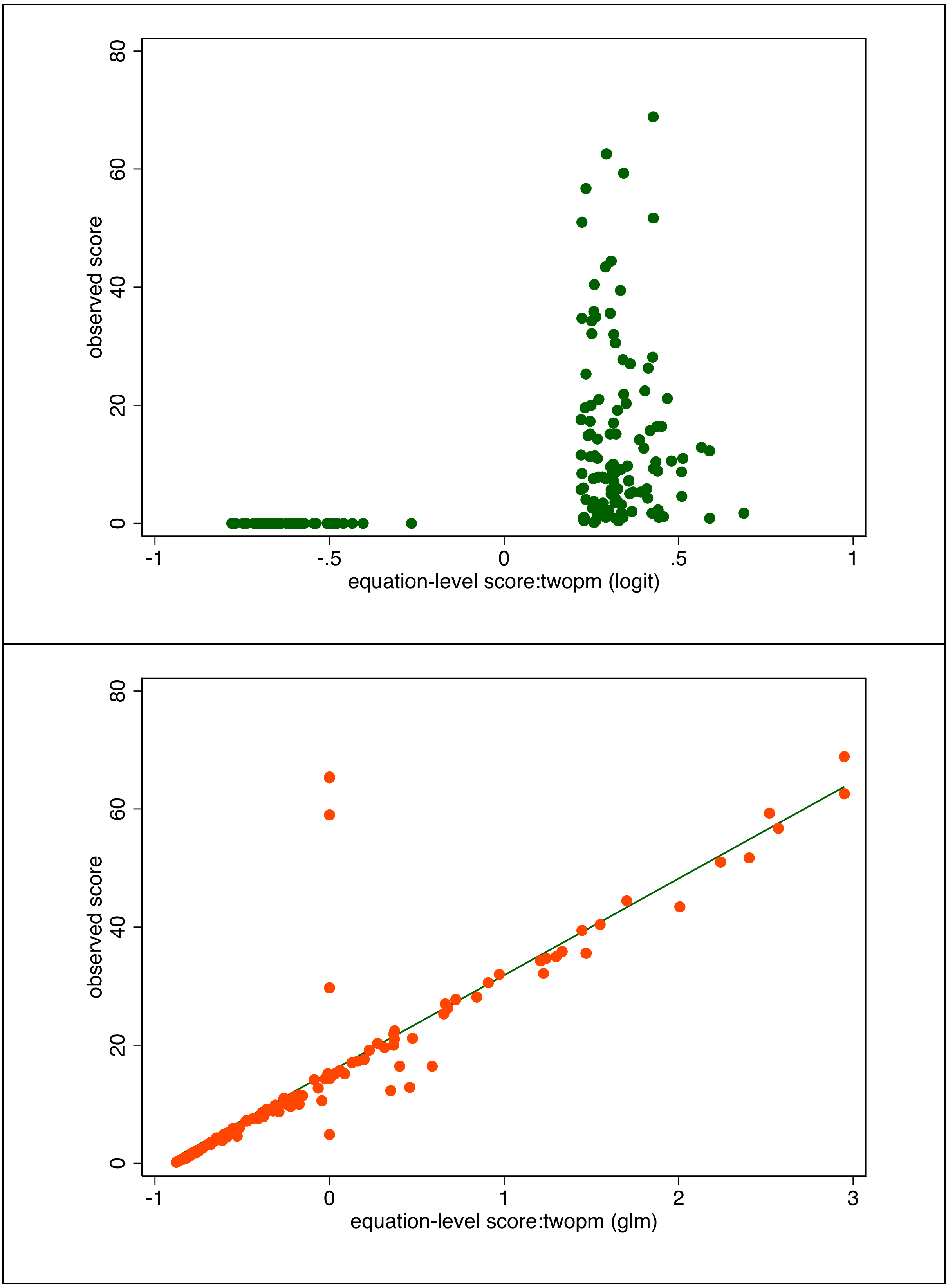

Supplemental Figure S31: relationships between the observed and predicted values for Anxiety (formal mindfulness practice model)

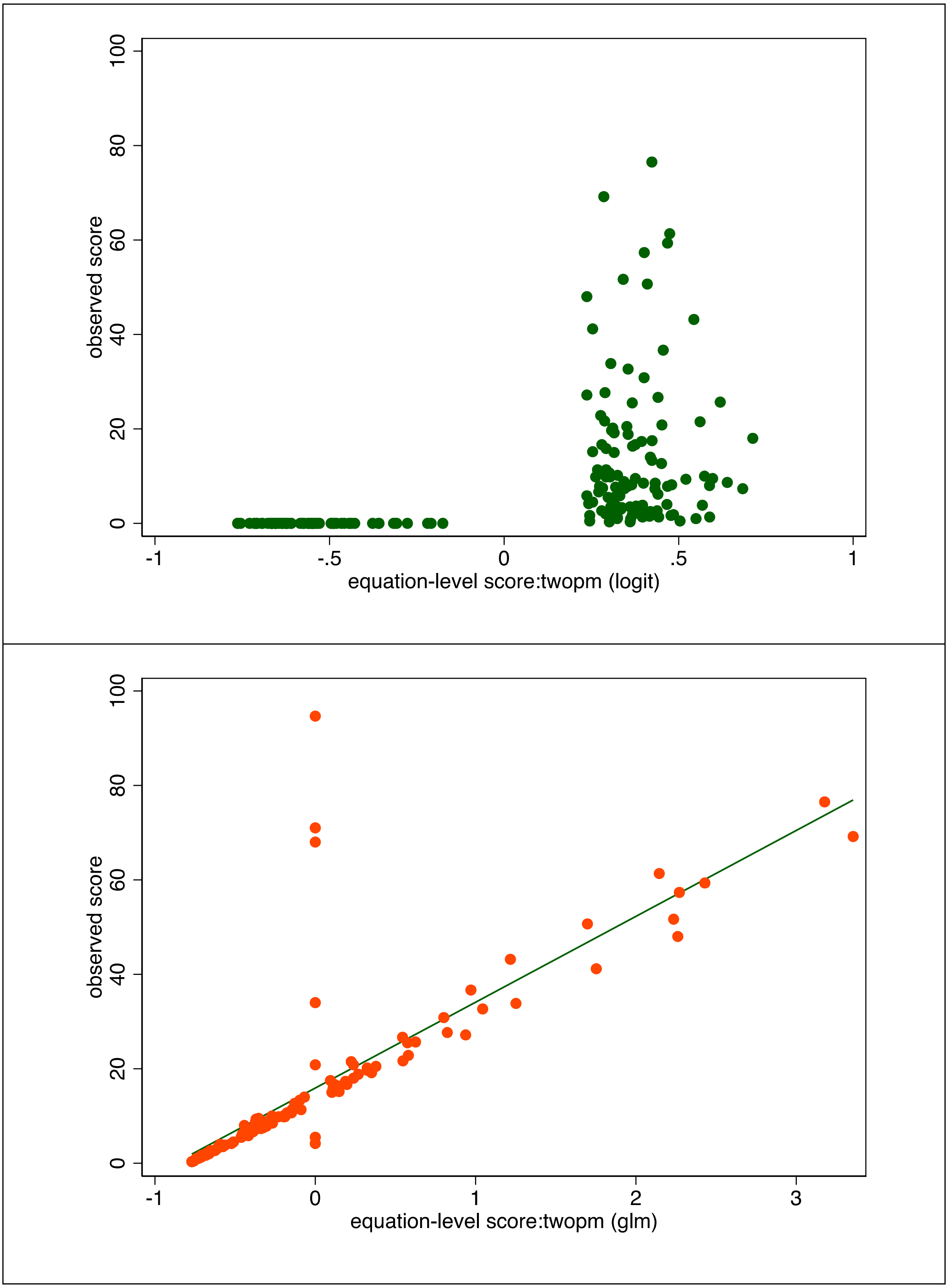

Supplemental Figure S32: relationships between the observed and predicted values for Complex imagery (formal mindfulness practice model)

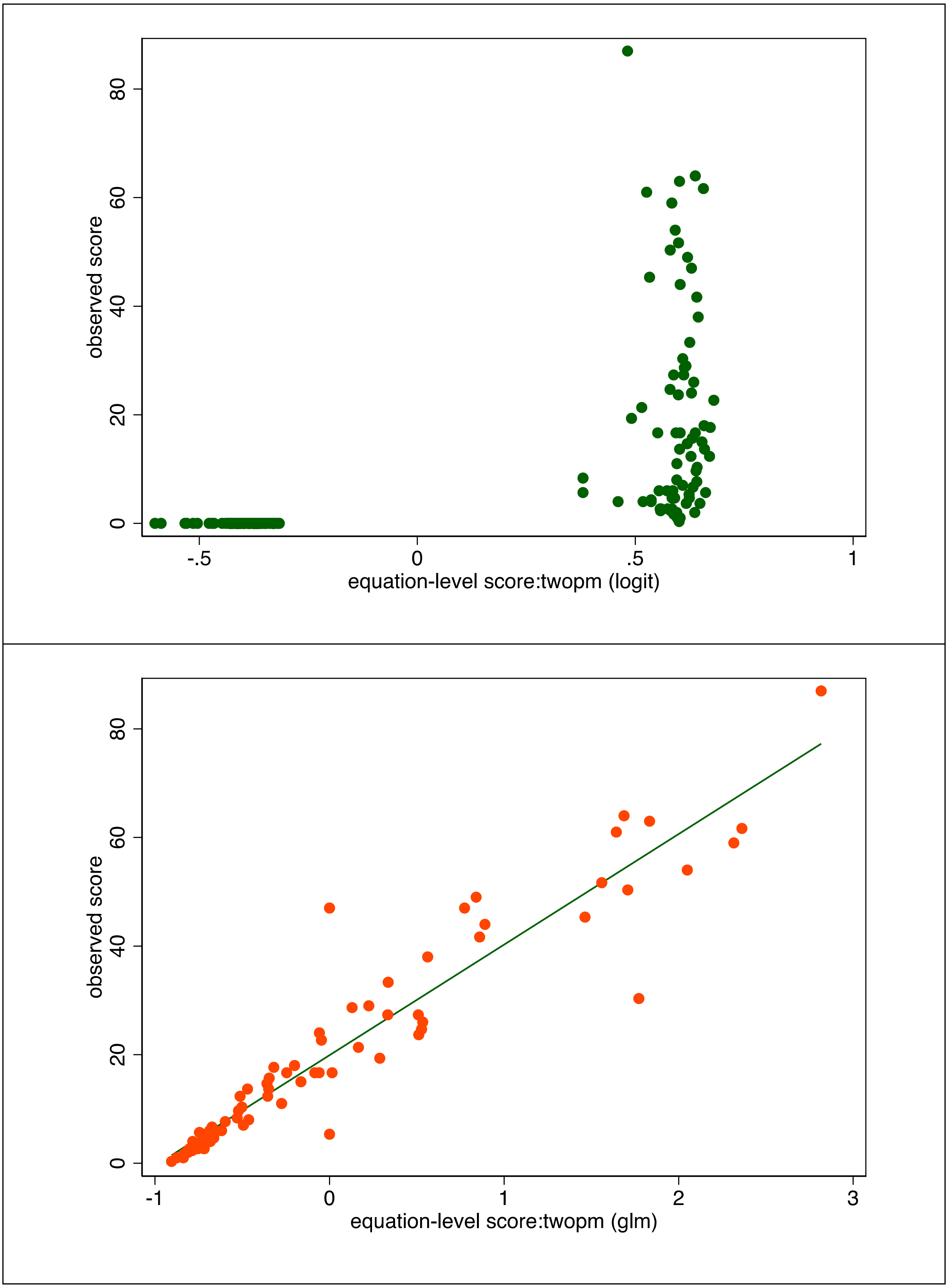

Supplemental Figure S33: relationships between the observed and predicted values for Elementary imagery (formal mindfulness practice model)

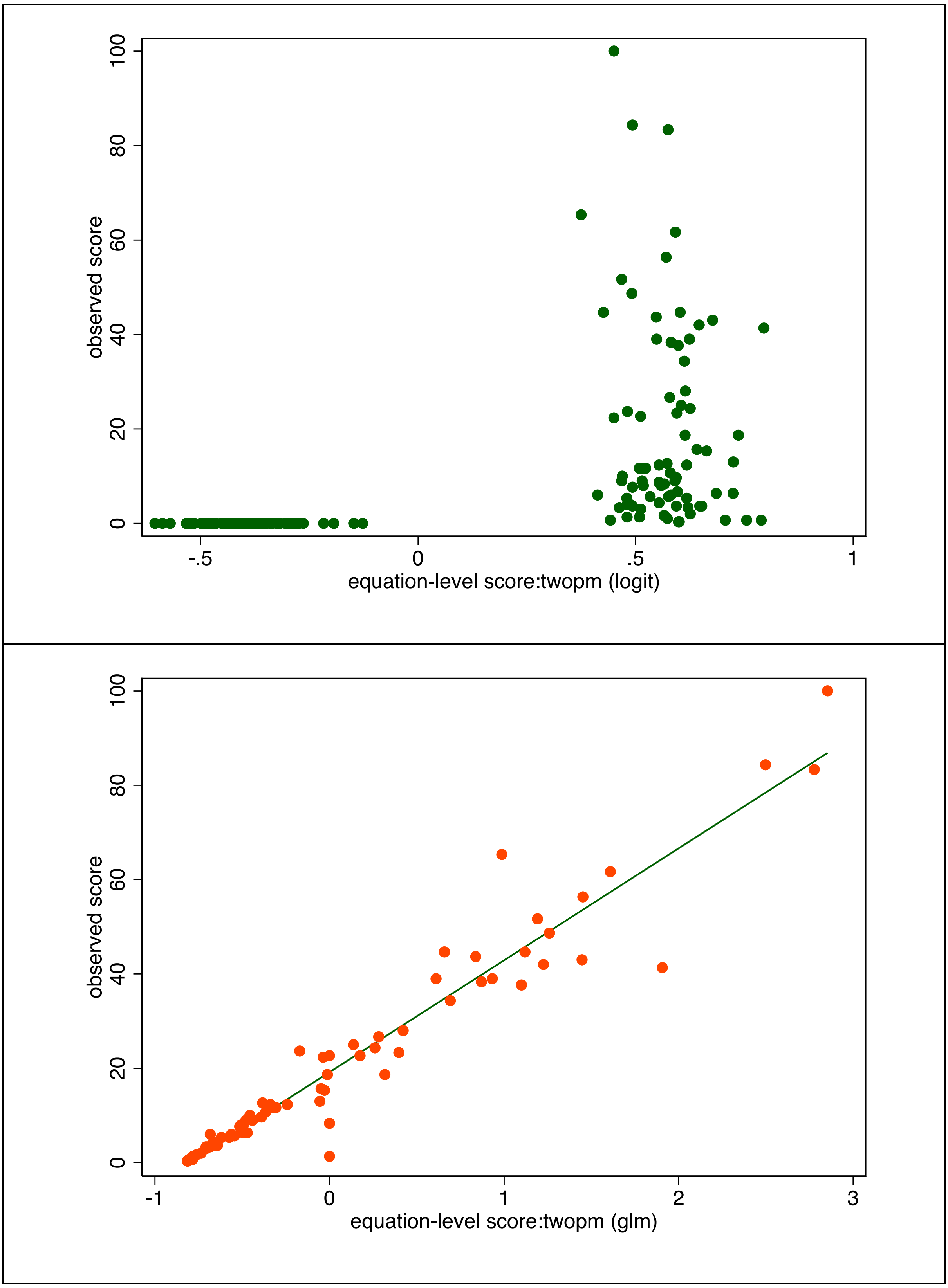

Supplemental Figure S34: relationships between the observed and predicted values for Audiovisual synesthesiae (formal mindfulness practice model)

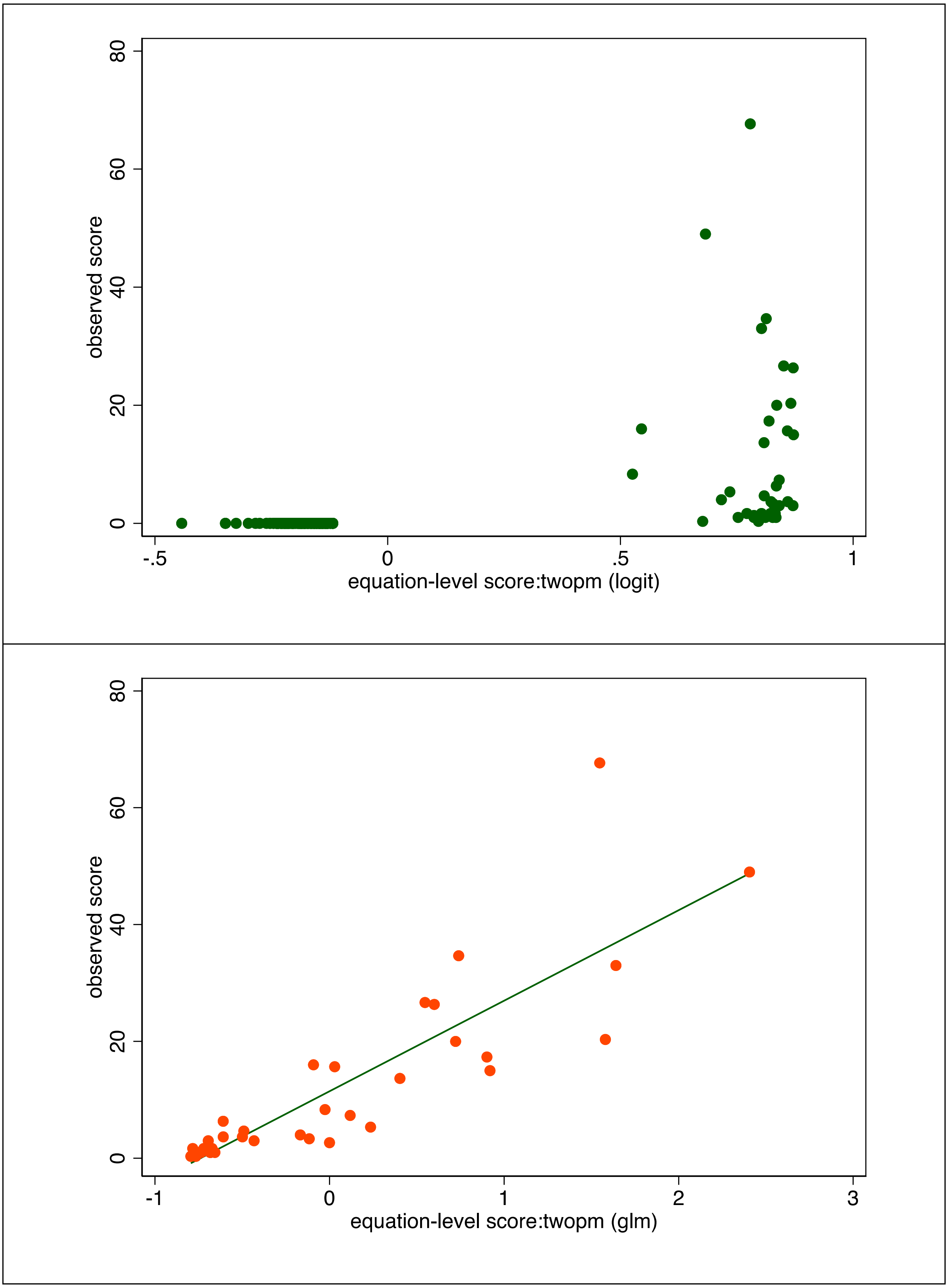

Supplemental Figure S35: relationships between the observed and predicted values for Changed meaning of percepts (formal mindfulness practice model)

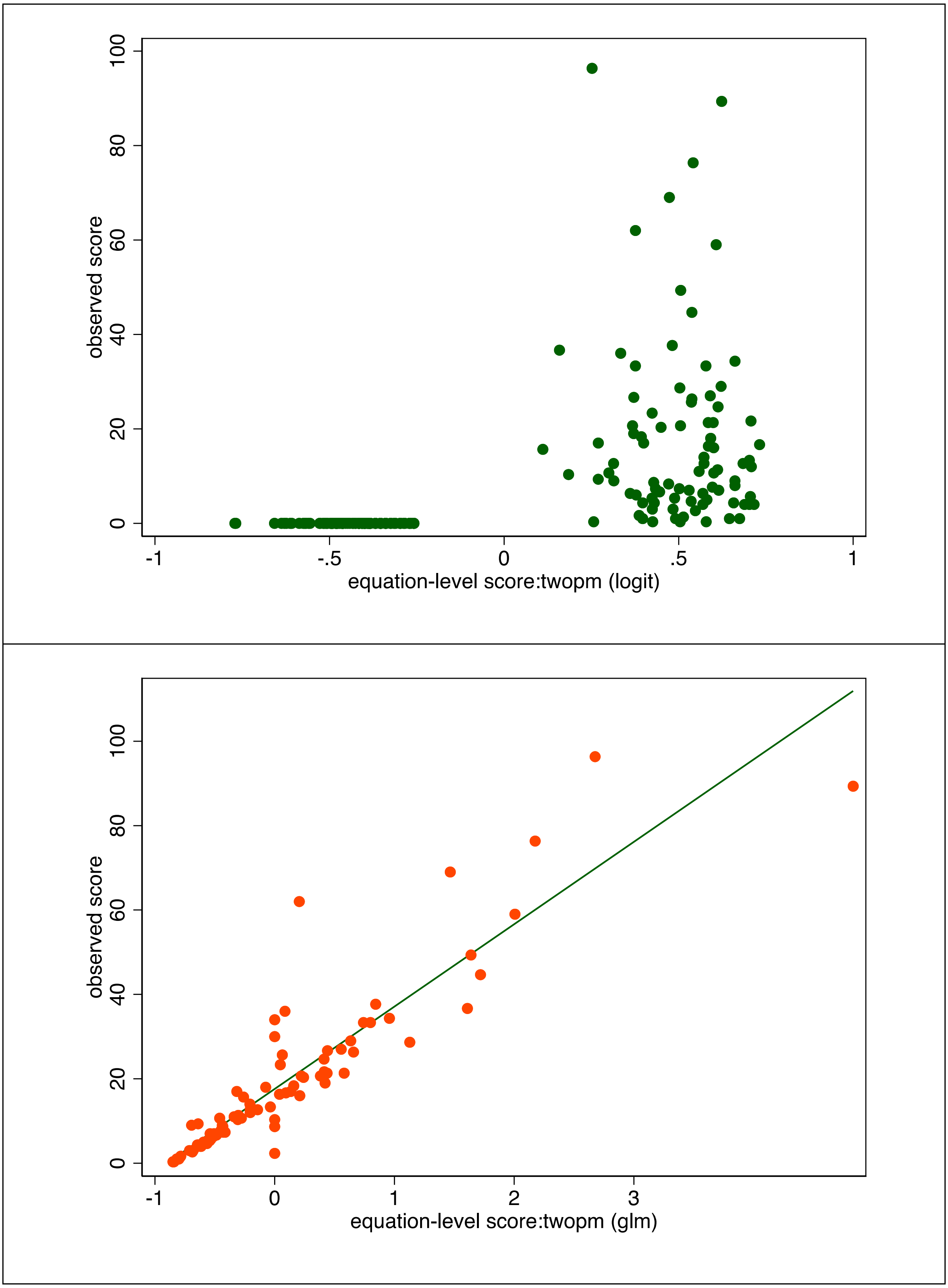

Supplement: S1 Appendix — (PDF) [file pone.0305928.s001.pdf]
