# Supplementary material for: Altered states of consciousness caused by a mindfulness-based programme up to a year later: Results from a randomised controlled trial
Source: PLoS One. 2024 Jul 17;19(7):e0305928. doi: 10.1371/journal.pone.0305928 (PMC11253948; doi:10.1371/journal.pone.0305928)
Supplement: S1 Protocol — (PDF) [file pone.0305928.s003.pdf]

BMJ Open

Protocol for the Mindful Student Study: a randomised controlled trial of the provision of a mindfulness intervention to support university students' well-being and resilience to stress

Julieta Galante,[1,2] Geraldine Dufour,[3] Alice Benton,[4] Emma Howarth,[2] Maris Vainre,[2] Timothy J Croudace,[5] Adam P Wagner,[2,6] Jan Stochl,[1,2] Peter B Jones[1,2]

To cite: Galante J, Dufour G, Benton A, *et al*. Protocol for the Mindful Student Study: a randomised controlled trial of the provision of a mindfulness intervention to support university students' well-being and resilience to stress. *BMJ Open* 2016;**6**: e012300. doi:10.1136/bmjopen-2016-012300

▶ Prepublication history and additional material is available. To view please visit the journal (http://dx.doi.org/10.1136/bmjopen-2016-012300).

Received 14 April 2016
Revised 4 July 2016
Accepted 8 September 2016

For numbered affiliations see end of article.

Correspondence to
Dr Julieta Galante;
mjg231@cam.ac.uk

ABSTRACT

Introduction: Levels of stress in UK university students are high, with an increase in the proportion of students seeking help in recent years. Academic pressure is reported as a major trigger. Mindfulness training has been shown to reduce stress and is popular among students, but its effectiveness in this context needs to be ascertained. In this pragmatic randomised controlled trial, we hypothesise that the provision of a preventative mindfulness intervention in universities could reduce students' psychological distress during the examination period (primary outcome), improve their resilience to stress up to at least 1 year later, reduce their use of mental health support services and improve academic performance.

Methods and analysis: At least 550 University of Cambridge students free from active crises or severe mental illness will be randomised to joining an 8-week mindfulness course or to mental health provision as usual (one-to-one allocation rate). Psychological distress will be measured using the Clinical Outcomes in Routine Evaluation Outcome Measure at baseline, postintervention, examination term and 1-year follow-up. Other outcomes are use of mental health services, inability to sit examinations or special circumstance requests, examination grades, well-being, altruism and coping measured with ecological momentary assessment. Outcome assessment and intention-to-treat primary analysis using linear mixed models adjusted for baseline scores will be blind to intervention allocation. We will also conduct per-protocol, subgroup and secondary outcome analyses. An Independent Data Monitoring and Ethics Committee will be set up. We will systematically monitor for, and react to, possible adverse events. An advisory reference group will comprise student representatives, members of the University Counselling Service and other student welfare staff.

Ethics and dissemination: Approval has been obtained from Cambridge Psychology Research Ethics Committee (PRE.2015.060). Results will be published in peer-reviewed journals. A lay summary will be disseminated to a wider audience including other universities.

Trial registration number: ACTRN12615001160527; pre-results.

Strengths and limitations of this study

- One of the largest randomised controlled trials assessing mindfulness interventions and the largest involving students, to date.
- A pragmatic design evaluating the provision of a service, intended to inform university student welfare policies in the global context of massively increasing participation in higher education.
- Interdisciplinary team and horizontal co-production of research question and study design between researchers and stakeholders.
- Study design assesses the effectiveness of mindfulness (ie, whether it produces the expected results under 'real-world' settings), but does not test its efficacy (ie, whether mindfulness produces the expected results under ideal circumstances, such as perfect course attendance), or determine its specific effects.

INTRODUCTION

Background and rationale

University students show elevated levels of stress. Although mental illness rates among first year students appear to be lower than those of the general population, they surpass general population rates when undergraduates get to their second year.[1] Students report academic pressure as the biggest trigger of their mental health problems.[2] University Counselling Services in the UK have noted the constant increase in the proportion of students seeking help in recent

BMJ Open: first published as 10.1136/bmjopen-2016-012300 on 9 November 2016. Downloaded from http://bmjopen.bmj.com/ on June 13, 2024 by guest. Protected by copyright.

years.[3 4] At the University of Cambridge, 8.5% of the students required access to counselling in 2014. An effective preventative intervention is needed to help students cope better with academic life and develop resilience.

Mindfulness interventions have been shown to reduce stress and prevent depression in clinical and non-clinical populations.[5 6] Secular mindfulness training involves paying attention to the present moment on purpose and non-judgmentally.[7] It is popular among students and increasingly used to support them in the UK.[8] However, there is little evidence on the effectiveness of offering mindfulness training to this population or of any adverse effects. Previous randomised trials assessing mindfulness for supporting university students generally suffer from small sample sizes, lack of follow-up, low methodological quality and poor reporting.[9] The largest good-quality study randomised 288 medical and psychology Norwegian students to mindfulness-based stress reduction or a waitlist and found moderate postintervention effects on psychological distress and subjective well-being.[10] A recent systematic review which meta-analysed nine randomised and non-randomised studies found that mindfulness significantly reduced anxiety among university students (d=0.73; 95% CI 1.00 to 0.45).[11] A good-quality and adequately powered randomised evaluation including the wider spectrum of university students is needed to confirm previous findings, extend the follow-up period and provide a more complete view of the potential impact (positive and negative) of the provision of mindfulness training on university student life. The University of Cambridge Vice-Chancellor's Endowment Fund is supporting such evaluation for use by services, funders and policymakers, as well as to inform the University's own decisions about the provision of mindfulness for students.

Objectives

The proposed study aims to evaluate whether the provision of a mindfulness course to higher education students:

- Helps them to manage stress during the examination period;
- Improves their mental well-being and resilience to stress up to 1 year later;
- Reduces their use of mental health treatment and support services;
- Improves their engagement with student life, including their academic performance.

Our main hypothesis is that the provision of mindfulness training will reduce students' psychological distress during the examination period in comparison with students who have not been offered this provision.

Trial design

The study will be a pragmatic randomised controlled evaluation with two parallel arms and a one-to-one allocation rate testing the superiority of mindfulness training provision to no provision. University of Cambridge students will be randomised to joining a mindfulness course during the term they are starting plus mental health provision as usual (PAU), or to PAU alone. PAU comprises access to individual counsellors, mental health advisors and psychiatrists at the University of Cambridge Counselling Service (UCS), as well as access to welfare staff in the University colleges (this provision varies across colleges, but can include college nurse, counsellor, welfare officer or tutor) and National Health Services (NHS). Those allocated to PAU alone will be offered a mindfulness course 1 year later, providing they are still students at the University.

The mindfulness intervention was offered for two terms before study initiation; this allowed the intervention to become established before evaluating it, and provided feasibility and acceptability data. The present proposal is partly based on the experience during those two terms. Interest in the courses doubled teaching capacity. An opportunistic randomised evaluation was therefore considered reasonable.

METHODS

This protocol was prepared in accordance with SPIRIT 2013 statement.[12] The SPIRIT checklist is available as an online supplementary file. The trial registration process (ACTRN12615001160527) needs clarification. The protocol was submitted to the trial registry in time for prospective registration but an unforeseen delay at their fault led to a final retrospective registration date. This problem was acknowledged by the trial registry and did not increase risk of bias compared with routine prospective registration.

Eligibility criteria

Participant eligibility criteria for this study are unchanged from those used routinely by the UCS for mindfulness courses. They are all self-reported. The inclusion criteria are as follows:

A. Undergraduate and postgraduate University of Cambridge students in any year or course;
B. Who consider they can realistically attend at least seven sessions of the course.

The exclusion criteria are as follows:

A. Currently suffering from severe periods of anxiety or depression;
B. Experiencing severe mental illness such as hypomania or psychotic episodes;
C. Following recent bereavement or major loss;
D. Experiencing any other serious mental or physical health issue that would impact on their ability to engage with the course.

Students will be advised to contact the study team if they are unsure about their eligibility.

Intervention

The 8-week mindfulness course is called 'Mindfulness Skills for Students'. It consists of a secular, group-based skills training programme based on the course book

BMJ Open: first published as 10.1136/bmjopen-2016-012300 on 9 November 2016. Downloaded from http://bmjopen.bmj.com/ on June 13, 2024 by guest. Protected by copyright.

'Mindfulness: A Practical Guide to Finding Peace in a Frantic World',[13] and adapted for university students. This intervention aims to optimise experiences across a range of students and is not specifically developed for those students in the clinical range.

The sessions last for 90 min for the first session, and 75 min for the remaining sessions. There are eight weekly sessions, all run by Dr Elizabeth English, an experienced and certified mindfulness teacher. Each session includes two mindfulness meditations, the first embedding the meditation that the students have practised at home throughout the week; the second, introducing them to the new meditation that they will practice at home in the coming week. There are also periods of reflection and inquiry, helping the students to understand the nature of mindfulness, to deepen their learning and embed it into their everyday lives. A few simple models are used and developed throughout the course, to give the students some theoretical understanding of the concepts developed experientially. As is usual in mindfulness programmes, each session also includes interactive exercises, so that the students share their experience and get to know each other throughout the course, building a sense of safety and community.

Before and after each class, students receive an email from the mindfulness teacher. This reminds them of the themes covered in the previous class, and lets them know the topics coming up in the next class. These emails also include handy tips, poems and video clips. There is also a course handout available in hardcopy at each class that can also be downloaded via a link in the postclass email, which describes the home practice for the coming week. The home practice time varies through the course, starting at 8 min, and increasing to about 15–25 min/week plus ongoing reflection through the day. It includes meditations from the course book's compact disc and other mindfulness practices such as a mindful walk, mindful eating, habit breakers and so on. More practice is possible for those who want it, and students are encouraged not to miss a day, but to rather consider doing less on days when they are busy. A detailed intervention manual is available on request from the corresponding author.

Seven Mindfulness Skills for Students courses run in parallel each term (which only lasts 9 weeks in Cambridge) with up to 30 students each. Students need to choose a session time and day to attend each week but are encouraged to attend as many sessions as they can, so if they cannot make their usual session, they can attend an alternative session within the same week (session hopping). Students are contacted by email when they miss a session to check whether the absence is related to a negative experience with mindfulness and, subsequently, to offer support.

As this will be a pragmatic study, care will be taken not to interfere with or modify routine practices for intervention delivery. Therefore, there will be no ad hoc adherence optimisation procedures. Participants in the control group will be guaranteed a space in the following year's mindfulness course and will be requested to inform the research team should they decide to learn mindfulness elsewhere during the follow-up period.

Outcomes and data collection

Several outcomes will be measured and compared between mindfulness and control groups to assess the effects of the course. The primary outcome will be a self-reported global measure of psychological distress assessed during the examination term, the most stressful period of Cambridge students' academic year. Secondary outcomes are exploratory assessments that may help to describe mindfulness' effects in more focused ways. Outcomes are listed in table 1.

Psychological distress will be measured using the Clinical Outcomes in Routine Evaluation Outcome Measure (CORE-OM), a 34-item generic questionnaire which was designed to assess efficacy and effectiveness across multiple disciplines offering psychological therapies, and has been widely used with UK university students. It is scored on a five-point scale ranging from 0 (not at all) to 4 (most or all the time). The total score range is 0–136; this is usually divided by number of completed items to form a total mean score. CORE-OM has good convergent validity, internal and test–retest reliability and sensitivity to change.[14]

Students' subjective well-being will be assessed using the Warwick-Edinburgh Mental Wellbeing Scale (WEMWBS), a questionnaire that captures a broad conception of well-being. It consists of 14 items, each scored on a five-point scale ranging from 1 (none of the time) to 5 (all of the time). The WEMWBS has good validity, internal consistency and test–retest reliability with a sample of UK students (n=354) and general population (n=2075).[15]

Mental health services use will be assessed by asking students whether during the examination term they have requested help with mental health issues and stress from a range of resources (eg, psychiatrist, Samaritans). Participants will also be asked to what extent such problems may have impacted on their academic performance (eg, To what extent do you have problems affecting your study?) and whether in their view their academic course workload was manageable. Data on inability to sit examinations will be provided by the Student Registry. The UCS will provide the research team with information about which participants used their services and how frequently they were used.

Day-to-day coping during the examination period will be assessed by applying ecological momentary assessment based on the cognitive appraisal theory of coping.[16] Every morning for 2 weeks, six questions will be asked about coping with academic stress on the previous day. These data will also be collected for a week at baseline from the participants recruited in January 2016. Motivational relevance (How motivated did you feel by academic matters yesterday?, How stressed did you feel

BMJ Open: first published as 10.1136/bmjopen-2016-012300 on 9 November 2016. Downloaded from http://bmjopen.bmj.com/ on June 13, 2024 by guest. Protected by copyright.

Table 1 MSS study outcomes

Outcome	Source/measure	Variable type	Collection points
Use of mental health services	Self-reported	Nominal	One-year follow-up
Use of University Counselling Service	Routinely collected	Nominal	Baseline, 1-year follow-up
Perceived impact of problems on academic performance	Self-reported	Ordinal	Examination term
Examination grades and rankings	Routinely collected	Ordinal	Examination term
Special circumstances requests for examinations	Routinely collected	Nominal	Examination term
Inability to sit examinations (intermissions and degrading)	Routinely collected	Nominal	Examination term
Psychological distress	Self-reported: CORE-OM	Treated as interval	Baseline, postintervention, examination term, 1-year follow-up
Well-being	Self-reported: WEMWBS	Treated as interval	Baseline, postintervention, examination term, 1-year follow-up
Altruism	Behavioural	Ordinal	Postintervention, examination term, 1-year follow-up
Coping	Self-reported	Ordinal	Baseline (Lent), examination term
Physical activity	Behavioural (sensor)	Ratio	Baseline (Lent), examination term
Sleep times	Behavioural (sensor)	Ratio	Baseline (Lent), examination term

CORE-OM, Clinical Outcomes in Routine Evaluation Outcome Measure; MSS, Mindful Student Study; WEMWBS, Warwick-Edinburgh Mental Well-being Scale.

by academic matters yesterday?), problem-focused coping potential (Did you study as much as you had planned yesterday?, Did you take as many breaks from study as you had planned yesterday?) and emotion-focused coping potential (How satisfied with yourself are you about the amount you studied yesterday?, How satisfied with yourself are you about the breaks from study you took yesterday?) will be assessed. Participants with an Android smartphone will be able to install a free application ('EasyM', developed by the University of Cambridge Computer Laboratory[17]) that will send notifications and display the questions. Other participants will receive a text message notification with a link to an online survey. In order to see how disrupted students' healthy routines become during the examination period, physical activity and sleep pattern data will be passively collected from Android users by the EasyM app using movement sensors (built-in accelerometer) for 2 weeks.

In view of evidence that mindfulness may stimulate altruism,[18] and that altruistic actions are associated with increased well-being,[19] we are exploring altruistic behaviour differences between groups. A sum of money in the form of Amazon vouchers will be offered to participants after completing each questionnaire (£3 for postintervention and 1-year follow-up questionnaires, £5 for the examination term questionnaire which will measure the primary outcome). A choice will be given as to whether to keep the token or to donate it to a local mental health charity. This will constitute an objective measure of altruism.

Process measures will involve: (1) registering attendance at mindfulness courses (register taken) and asking why sessions were missed (routine practice for UCS); (2) asking students whether they did their mindfulness homework during the course and how much they have practised after the course, including whether they became members of the Mindfulness Society; (3) for students who abandon the study (ie, fail to complete questionnaires or contact us saying they wish to quit the study), information on why they have done so will be requested; (4) participants in the control group will be asked whether they have practised mindfulness elsewhere during the follow-up period.

Apart from the baseline measurements outlined in table 1, the following baseline data will be collected in order to compare the sample with the student population, and to run subgroup analyses: (1) students' prior experience with meditation and mindfulness; (2) demographic data provided by the student registry (eg, disability, ethnicity, socioeconomic classification). All baseline data will be collected before randomisation.

Questionnaires will be web-based. Privacy issues related to accessing student records and collecting data from smartphone sensors were explored in a focus group with students who completed mindfulness courses taught before the trial. Students felt these methods were acceptable.

Sample size

The minimum sample size required was calculated to detect a 0.3 SD change in psychological distress with CORE-OM, the primary outcome. This change constitutes a small difference, but is reasonable for a relatively short mindfulness course, and attractive if this shift happens at a community rather than a clinical level.[20]

A study of a non-clinical sample (746 students from two UK universities plus a community sample of 360 people) found a mean total score of 0.76 points and a SD of 0.59 points.[21] To detect a change of 0.3 SDs at $p<0.05$ with

BMJ Open: first published as 10.1136/bmjopen-2016-012300 on 9 November 2016. Downloaded from http://bmjopen.bmj.com/ on June 13, 2024 by guest. Protected by copyright.

90% power, 550 students (275 per arm) are estimated to be needed, allowing for 20% loss to follow-up as informed by previous studies (eg, Warnecke *et al*[22]).

Recruitment

Students will be recruited in two waves, in October 2015 (beginning of Michaelmas term) and January 2016 (beginning of Lent term). Figure 1 shows the participant timeline.

The evaluation will be advertised widely in the student community at the University of Cambridge. Posters will be put up in the University buildings. Facebook and Twitter study accounts will be used. Colleges will circulate an email presenting the study and inviting students to attend the information sessions at the beginning of both terms. The students' Mindfulness Society has agreed to direct students who approach them with an interest in learning mindfulness to the information sessions. All materials will display a dedicated email address for contacting the study team.

Advertising will focus on letting students know about the study and directing them to a dedicated website or to information sessions that will take place in the first weeks of each term. Both the website and the information sessions will provide prospective participants with detailed information about the study and consent procedures.

Blinding and randomisation procedures

After agreeing to take part, students will be emailed with a link to the online baseline questionnaire. Only those who complete the baseline questionnaire will be randomised. Simple randomisation will be carried out remotely by the survey software (Qualtrics) using computer-generated random numbers. Participants will be informed of their allocation automatically after completing the baseline questionnaire. This way the allocation process will be concealed from researchers.

Participants randomised to the intervention group will be requested to state which of the seven mindfulness session times on offer they would be able to attend. Then, to minimise attrition, an allocation optimisation programme will be run with these data to assign as many students as possible to one of their preferred course times.

Participants cannot be blind to allocation because of the nature of the intervention. However, outcome assessment will be blind because data collection is carried out remotely and automatically. The primary analysis will be carried out by a statistician blind to which arm is the intervention, and other variables/information which could be used to identify intervention arm data. Mindfulness courses will include participants who are not part of the trial (consisting of up to 60 interested students distributed evenly across courses so that 4–5 students out of 30 per course are not part of the study), and the mindfulness teacher will not be told who is and who is not a participant in the study.

Inducements for participation

There will be no inducements for completing the mindfulness courses. However, to promote participant retention and as a token of appreciation for completing all the study questionnaires, a total of £11 will be available to each student across the study in the form of Amazon vouchers as explained above. In addition, there will be a prize draw of 5×£100 Amazon vouchers among those who complete 50% or more smartphone questions plus all the questionnaires. Students who complete 50% or

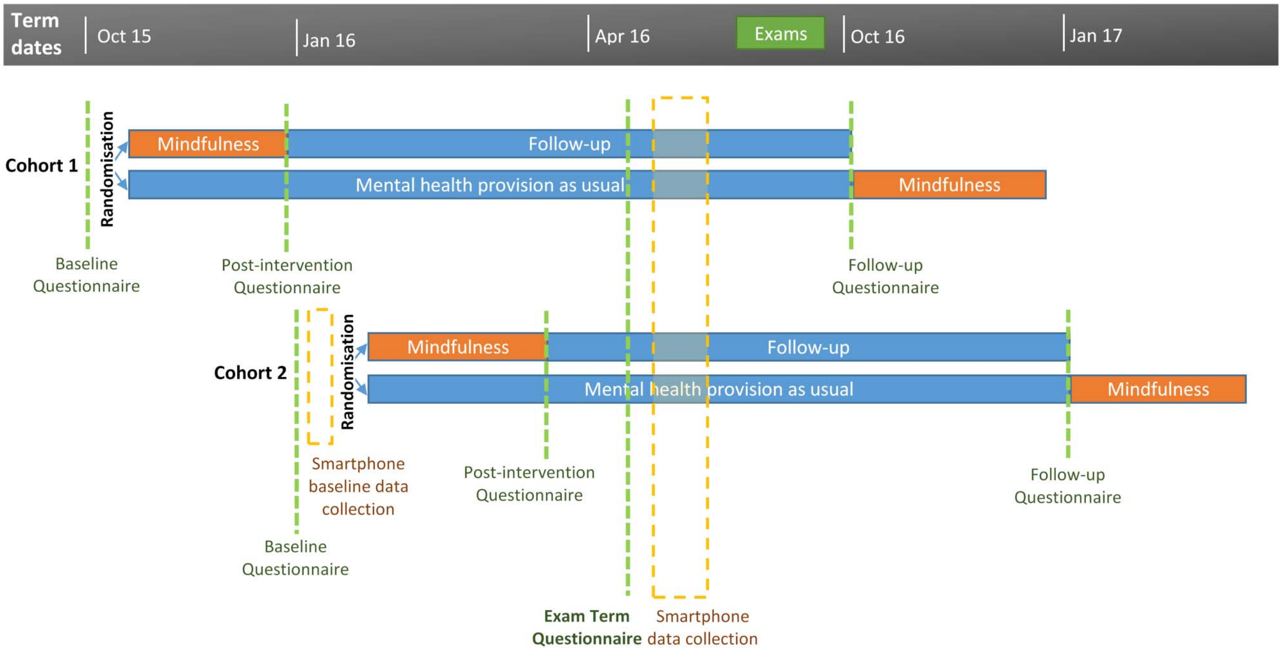

Figure 1 Participant timeline.

BMJ Open: first published as 10.1136/bmjopen-2016-012300 on 9 November 2016. Downloaded from http://bmjopen.bmj.com/ on June 13, 2024 by guest. Protected by copyright.

more smartphone notifications will be offered individual feedback on their coping, sleep and physical activity patterns after the study ends.

Public engagement

Involving stakeholders in the choice of question and design of the research is important to ensure relevance.[23] The study plans presented here were reviewed by a group comprising representatives from the UCS, the Academic Division, student representatives and college tutors. A focus group with students who completed mindfulness courses taught before the trial was consulted about the study plans before submission to the Ethics Committee for approval.

An advisory reference group will be put together comprising student representatives, members of the University Counselling Service and other student welfare staff. They will meet three times a year. Study researchers will attend these meetings and present updates. Reference group terms of reference will be available on request.

Statistical methods

The primary analysis will consist of an intention-to-treat analysis comparing the primary outcome, CORE-OM during the examination period, between arms adjusted for baseline scores, routine demographics and timing of receipt of intervention relative to examinations (as some will have done the course during Michaelmas 2015 and others during Lent 2016). Multiple imputation will be used as long as there are <40% missing data in the corresponding variable to ensure validity of imputations and will be applied only to variables with expected missing completely at random and missing at random patterns (ie, when there are no reasons to think that the pattern may be missing not at random). This imputation will take account of other CORE-OM data points and routinely collected demographics. We will also conduct a per-protocol analysis (minimum dose assumed to be 50% attendance of sessions[24]) excluding individuals in the control group who have engaged in meditation elsewhere during the follow-up period preceding outcome measurement.

Outcomes measured at three time points (CORE-OM, WEMWBS and altruism, measured at postintervention, examination period and 1-year follow-up) will be analysed using a repeated measures design with a treatment by time interaction term to study their trajectories through the academic year and to determine whether differences (ie, intervention effects) were consistent over time. Repeated measures analyses will also be performed with ecological momentary assessment data to study outcome trajectories, pattern changes during the examination period and differences between arms.

CORE-OM and WEMWBS data will be combined to explore the broader spectrum of distress/well-being if taken as a continuum.[25] Subscales of the CORE-OM (subjective well-being, problems symptoms, functioning, risk/harm) will also be explored as secondary outcomes, and results reported with and without correction for multiple testing. Multilevel models will be used to assess academic degrees and academic rankings as any student may sit more than one examination.

The following predefined subgroup analyses will be conducted on the primary outcome by using interaction tests:[26]

- By degree, as most have examinations during the examination term but some do not;
- By year of study, to explore whether results differ for last year students, a different subpopulation as control group final year students will not be offered mindfulness a year later;
- By baseline CORE-OM: those initially worse may drive change;
- By gender: there is evidence of differential impact;[10]
- By amount of home practice during and after course in intervention group participants;
- By prior meditation experience (prior 8-week course or +50 hours spent meditating in the past—an 8-week course translates into 10–50 hours) as only novices may experience a change.

In order to assess how our sample compares against the student population in the UK, demographic and normative well-being/distress data will be obtained from the literature and compared with baseline values in our sample. A comparison of our baseline data with the profile of students attending the University Counselling Service will also be performed where possible to evaluate where our sample lies in the range between community and clinical student samples.

All statistical analyses will be conducted at an α level of $p=0.05$ (two-sided). Linear mixed models will be used for the analyses. Assumptions will be tested and diagnostic plots will be explored to assess model fit. Descriptive statistics for continuous variables will be summarised using mean/SD and median/IQR. Discrete variables will be summarised by proportions.

It is expected that the clustering effect will be negligible: although this is a group intervention, the work is highly personal, all the courses are taught by the same teacher, each course includes students from different colleges and courses, and the 'session hopping' option introduces variability. However, we will compute intraclass correlation by analysing session attendance patterns to see whether there is any clustering effect. If there is one, we will adjust for it using multilevel techniques.

Data monitoring and adverse events

An Independent Data Monitoring and Ethics Committee (IDMEC) will be set up comprising an independent chair familiar with student welfare issues, an independent researcher, a representative from the student body and a representative from the colleges that make up the University. Its role will be to safeguard the interests of trial participants, assess the safety and efficacy of the intervention during the trial, and monitor the overall conduct of the trial. The IDMEC will meet

BMJ Open: first published as 10.1136/bmjopen-2016-012300 on 9 November 2016. Downloaded from http://bmjopen.bmj.com/ on June 13, 2024 by guest. Protected by copyright.

three times a year and make recommendations to the researchers; its terms of reference are available on request and include provision for terminating the trial early. There are no plans for interim analyses, although the IDMEC could request them.

Introductory, 8-week mindfulness courses for people who meet our selection criteria are not known to be associated with adverse events. However, we will systematically monitor for such events and have a duty of care to react when there is an indication of extreme distress or risk in a student. Participants will be encouraged on enrolment to look for signs of their mental or physical health deteriorating, whether or not it is related to the mindfulness course. Emergence of such symptoms will be considered adverse events. Subsequently, during the study, there will be three ways of identifying adverse events:

1. There may be uncomfortable moments during the mindfulness course as participants are requested to turn their attention to whatever thoughts are coming into their minds. They will be taught how to safely deal with these thoughts, but initial experiences can be somewhat distressing. Participants are frequently encouraged to approach the course teacher to discuss any concerns.
2. All participants will complete the CORE-OM questionnaire at baseline, postintervention, during the examination term and at 1-year follow-up. The study team will monitor the risk subscales of CORE-OM each time participants complete it (as stated in the participant information sheet). Studies support using the following cut-off scores as markers of significant risk: 3 or more for the self-harm risk subscale, 3 or more for the harm to others risk subscale, or 5 or more for the suicide risk subscale.[27 28] Scores of 7 or more points in any subscale will be prioritised.
3. All the trial participants will be requested to let the study team know if and why they are planning to leave the study.

In the event of any adverse events emerging, participants will be contacted, strongly encouraged to seek additional help and directed to relevant health services. If a participant fails to respond or refuses to access help without reasonable justification, they will be informed that the research team will try to contact support services (eg, college nurse) without their consent (as stated in the participant information sheet). Events will be recorded on a structured form sent to the IDMEC Chair who will determine whether they could be related to the intervention (ie, adverse reactions[29]) and how to proceed (eg, stopping the trial early).

ETHICS AND DISSEMINATION

Protocol amendments will be prepared by the study researchers in consultation with the IDMEC and the reference group. Ethical approval will be sought. The Trial Registry and the Research Governance Office will be informed.

Consent

After reading the participant information sheet, students will be able to consent online or in person (a copy of the consent form is available as an online supplementary file). The electronic and paper-based information sheets and consent forms will have the same content. They will clearly state eligibility criteria and request students to self-assess whether they meet them. They will also list other mental health support resources (eg, University Counselling Service) within and outside the University for those who do not wish or cannot take part in this study.

Information sessions will be set up in central locations on different days where a member of the research team will distribute participant information sheets. They will give plenty of time for students to read them and ask all the questions they need. They will also be able to take the information sheet with them and come back later or use the website to consent. The mindfulness teacher will be either present at the sessions or reachable by phone and email for students to ask questions about the course.

The online consent will be programmed in Qualtrics and participants' consents will be recorded in a secure database. An 'I agree' button will allow participants to continue answering baseline questionnaires, those who do not consent will not be able to continue answering, so no personal information about them will be recorded online before they consent. If a student reads the participant information on the website and has questions, they will be able to phone and email the research team, or attend the information sessions. They will be emailed a copy of the consent for their records.

Students in their final year have a 50% chance of assignment to the control group and may not be able to receive the mindfulness course in the following year unless they stay on for another degree. This issue was explored in the focus group with students and it did not raise any significant concerns. However, this circumstance will be made clear in the participant information sheet for last year students to make an informed decision on whether to take part in the study. In any case, participants randomised to the control group will not be requested to avoid learning mindfulness elsewhere, and a list of resources to do so will be offered in the study website.

Data management

Identifiable research data will be stored at the Clinical School's Secure Data Hosting Service, only accessible by the data manager (APW), the principal investigator (PBJ) and the trial manager (JG). From here, an anonymised copy blind to which arm will be the intervention arm will be made to be used for the independent statistician (JS) who will conduct the primary analysis. During the conduct of the trial the independent statistician will be excluded from any information that would help identify the arms.

BMJ Open: first published as 10.1136/bmjopen-2016-012300 on 9 November 2016. Downloaded from http://bmjopen.bmj.com/ on June 13, 2024 by guest. Protected by copyright.

Adverse event reports will be personally identifiable but kept strictly confidential. Only some members of the research team (GD, PBJ, MV, JG, APW), the mindfulness teacher and the IDMEC chair will have access to them.

Dissemination policy

Findings will be submitted to high-impact peer-review journals. Publication authorship will be based on the International Committee of Medical Journal Editors' criteria.

We will also send a briefing to other universities and a lay summary to participating students. Further dissemination will take place by developing an online interactive social media presence, taking part in public engagement events, and using media channels.

Author affiliations

[1]Department of Psychiatry, University of Cambridge, Cambridge, UK
[2]NIHR Collaboration for Leadership in Applied Health Research and Care (CLAHRC) East of England, Cambridge, UK
[3]University Counselling Service, University of Cambridge, Cambridge, UK
[4]Academic Division, University of Cambridge, Cambridge, UK
[5]Dundee Centre for Health and Related Research, School of Nursing and Health Sciences, University of Dundee, Dundee, UK
[6]Norwich Medical School, University of East Anglia, Norwich, UK

Acknowledgements The authors would like to thank Elizabeth English for her independent development of the intervention; Tim Dalgleish, Loraine Gelsthorpe and Sandrine Müller for their helpful advice as members of the IDMEC; David Spiegelhalter for his comments on an early draft of the protocol and his help with publicising the study; Neal Lathia for providing the 'Easy M' app; and the developer who designed the allocation optimisation programme.

Contributors GD and AB had the idea for a mindfulness intervention pilot; PBJ, AB and GD applied for funding for a randomised evaluation; JG and PBJ produced an initial draft of the protocol that was revised through discussion with TJC, GD, AB, EH and MV; the analysis plan was devised by JG, PBJ, JS and AW. JG is the lead researcher; PBJ is the guarantor of the study.

Funding This article presents independent research funded by the University of Cambridge Vice-Chancellor's Endowment Fund, the University Counselling Service and the National Institute for Health Research (NIHR) Collaboration for Leadership in Applied Health Research and Care (CLAHRC) East of England, at Cambridgeshire and Peterborough NHS Foundation Trust.

Disclaimer The views expressed are those of the authors and not necessarily those of the University of Cambridge, the NHS, the NIHR or the Department of Health.

Competing interests None declared.

Patient consent Obtained.

Ethics approval Approval was obtained from the Cambridge Psychology Research Ethics Committee on 25/08/2015 (PRE.2015.060).

Provenance and peer review Not commissioned; externally peer reviewed.

Open Access This is an Open Access article distributed in accordance with the terms of the Creative Commons Attribution (CC BY 4.0) license, which permits others to distribute, remix, adapt and build upon this work, for commercial use, provided the original work is properly cited. See: http://creativecommons.org/licenses/by/4.0/

REFERENCES

1. Macaskill A. The mental health of university students in the United Kingdom. *Br J Guidance Couns* 2013;41:426–41.
2. Kerr H. Mental Distress Survey Overview: NUS Services Limited; May 2013. https://www.nus.org.uk/Global/Campaigns/20130517%20Mental%20Distress%20Survey%20%20Overview.pdf.
3. Mair D. The rise and rise of higher education and therapeutic culture. In: Mair D, ed. *Short-term counselling in higher education—context, theory and practice*. Abingdon: Routledge, 2016:7–26.
4. Williams M, Coare P, Marvell R, *et al. Understanding provision for students with mental health problems and intensive support needs*. Institute for Employment Studies and Researching Equity, Access and Partnership, 2015.
5. Gotink RA, Chu P, Busschbach JJ, *et al*. Standardised mindfulness-based interventions in healthcare: an overview of systematic reviews and meta-analyses of RCTs. *PLoS ONE* 2015;10:e0124344.
6. Kendrick T, Peveler R. Guidelines for the management of depression: NICE work? *Br J Psychiatry* 2010;197:345–7.
7. Kabat-Zinn J. *Wherever you go, there you are: mindfulness meditation in everyday life*. New York: Hyperion, 1994.
8. Mindfulness for Students website. Mindfulness courses at UK Universities (27 January 2016). http://mindfulnessforstudents.co.uk/mindfulness-courses-at-uk-universities/
9. Bamber MD, Kraenzle Schneider J. Mindfulness-based meditation to decrease stress and anxiety in college students: a narrative synthesis of the research. *Educ Res Rev* 2016;18:1–32.
10. De Vibe M, Solhaug I, Tyssen R, *et al*. Mindfulness training for stress management: a randomised controlled study of medical and psychology students. *BMC Med Educ* 2013;13:107.
11. Regehr C, Glancy D, Pitts A. Interventions to reduce stress in university students: a review and meta-analysis. *J Affect Disord* 2013;148:1–11.
12. Chan AW, Tetzlaff JM, Altman DG, *et al*. SPIRIT 2013 statement: defining standard protocol items for clinical trials. *Ann Intern Med* 2013;158:200–7.
13. Williams M, Penman D. *Mindfulness: a practical guide to finding peace in a frantic world*. UK: Hachette, 2011.
14. Evans C, Connell J, Barkham M, *et al*. Towards a standardised brief outcome measure: psychometric properties and utility of the CORE-OM. *Br J Psychiatry* 2002;180:51–60.
15. Tennant R, Hiller L, Fishwick R, *et al*. The Warwick-Edinburgh Mental Well-being Scale (WEMWBS): development and UK validation. *Health Qual Life Outcomes* 2007;5:63.
16. Smith CA, Kirby LD. Putting appraisal in context: toward a relational model of appraisal and emotion. *Cogn Emotion* 2009;23:1352–72.
17. Lathia N. EasyM 2016. (10 April 2016). https://play.google.com/store/apps/details?id=com.lathia.easym&hl=en
18. Galante J, Galante I, Bekkers MJ, *et al*. Effect of kindness-based meditation on health and well-being: a systematic review and meta-analysis. *J Consult Clin Psychol* 2014;82:1101–14.
19. Post SG. Altruism, happiness, and health: it's good to be good. *Int J Behav Med* 2005;12:66–77.
20. Huppert FA. A new approach to reducing disorder and improving well-being. *Perspect Psychol Sci* 2009;4:108–11.
21. Core System Group. *CORE system user manual*. Leeds: Core System Group. (15 September 2015). http://www.coreims.co.uk/index.html
22. Warnecke E, Quinn S, Ogden K, *et al*. A randomised controlled trial of the effects of mindfulness practice on medical student stress levels. *Med Educ* 2011;45:381–8.
23. Craig P, Dieppe P, Macintyre S, *et al. Developing and evaluating complex interventions: new guidance*. UK: Medical Research Council, 2008.
24. Gu J, Strauss C, Bond R, *et al*. How do mindfulness-based cognitive therapy and mindfulness-based stress reduction improve mental health and wellbeing? A systematic review and meta-analysis of mediation studies. *Clin Psychol Rev* 2015;37:1–12.
25. Böhnke JR, Croudace TJ. Calibrating well-being, quality of life and common mental disorder items: psychometric epidemiology in public mental health research. *Br J Psychiatry* 2016;209:162–8.
26. Pocock SJ, Assmann SE, Enos LE, *et al*. Subgroup analysis, covariate adjustment and baseline comparisons in clinical trial reporting: current practice and problems. *Stat Med* 2002;21:2917–30.
27. Whewell P, Bonanno D. The Care Programme Approach and risk assessment of borderline personality disorder: clinical validation of the CORE risk sub-scale. *Psychiatr Bull* 2000;24:381–4.
28. Bedford A, Lukic G, Tibbles J. Evaluation of risk by patients' and with clinicians' ratings: a CORE-OM and CORE-A investigation. *Clin Psychol Psychother* 2011;18:244–9.
29. NHS National Institute for Health Research. Clinical Trials Toolkit. 2016. (1 September 2015). http://www.ct-toolkit.ac.uk/

BMJ Open: first published as 10.1136/bmjopen-2016-012300 on 9 November 2016. Downloaded from http://bmjopen.bmj.com/ on June 13, 2024 by guest. Protected by copyright.
